# Novel NHC-Based Au(I) Complexes as Precursors of Highly Pure Au(0) Nuggets under Oxidative Conditions

**DOI:** 10.3390/molecules28052302

**Published:** 2023-03-01

**Authors:** Pau Font, Nikolaos V. Tzouras, Argyro T. Papastavrou, Georgios C. Vougioukalakis, Xavi Ribas

**Affiliations:** 1 Institut de Química Computacional i Catàlisi and Departament de Química, Universitat de Girona, Campus Montilivi, E-17003 Girona, Catalonia, Spain; 2 Department of Chemistry, National and Kapodistrian University of Athens, Panepistimiopolis, 15771 Athens, Greece

**Keywords:** NHC-Au(I) complexes, pendant ligands, iodosylbenzene-type oxidants, oxidized NHC=O compounds, Au(0) nuggets

## Abstract

The Lewis-acidic character and robustness of NHC-Au(I) complexes enable them to catalyze a large number of reactions, and they are enthroned as the catalysts of choice for many transformations among polyunsaturated substrates. More recently, Au(I)/Au(III) catalysis has been explored either by utilizing external oxidants or by seeking oxidative addition processes with catalysts featuring pendant coordinating groups. Herein, we describe the synthesis and characterization of N-heterocyclic carbene (NHC)-based Au(I) complexes, with and without pendant coordinating groups, and their reactivity in the presence of different oxidants. We demonstrate that when using iodosylbenzene-type oxidants, the NHC ligand undergoes oxidation to afford the corresponding NHC=O azolone products concomitantly with quantitative gold recovery in the form of Au(0) nuggets ~0.5 mm in size. The latter were characterized by SEM and EDX-SEM showing purities above 90%. This study shows that NHC-Au complexes can follow decomposition pathways under certain experimental conditions, thus challenging the believed robustness of the NHC-Au bond and providing a novel methodology to produce Au(0) nuggets.

## 1. Introduction

Fundamental comprehension of the intrinsic reactivity of NHC-based gold complexes is needed to evaluate their success in different fields, such as in the biological sector [1,2,3,4,5] or in catalysis. NHC ligands are typically described as strongly σ-donating, which form very stable NHC-metal bonds throughout a catalytic cycle [6,7]. Complexes of transition metals with N-heterocyclic carbene (NHC) ligands are commonly applied as catalysts for a variety of C-C and C-heteroatom bond formations, C-H functionalizations, cross-couplings, atom-economic additions, olefin metathesis, and many other transformations [8,9,10,11,12]. Moreover, there is high variability of the steric and electronic parameters of NHC ligands, which allows effective tuning of the catalyst activity [13].

**Figure 1:**
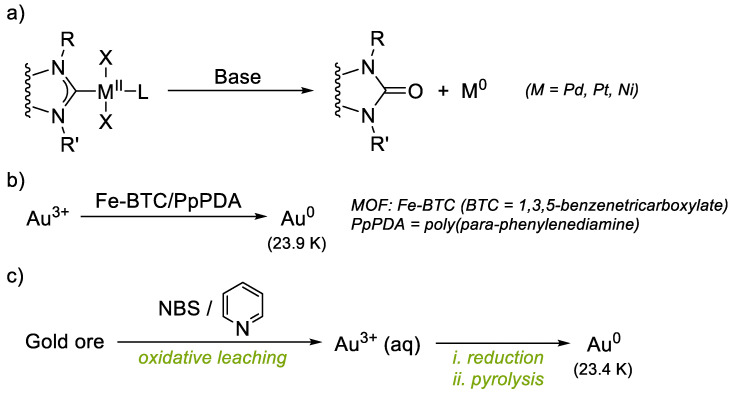
(**a**) Examples of azolone decomposition pathways of NHC-M, and (**b**,**c**) strategies to recover highly pure Au(0) (>23 karats).

Focusing on gold catalysis, a large number of NHC-Au complexes have been successfully used in Lewis acid catalysis for the cyclization and rearrangements of polyunsaturated substrates [14,15]. However, in order to further expand the scope of applications of these complexes, it is necessary to explore experimental conditions where NHC-gold complexes might suffer from instability. For instance, the exploration of Au(I)/Au(III) catalysis using external oxidants or oxidant-free catalysis with ligands featuring pendant coordinating groups has become prominent in the last decade, but the intrinsic decomposition of gold complexes is underexplored. In a related work, Ananikov reported the unusual role of bases in the deactivation of NHC–metal catalysts (metal = Ni(II), Pd(II) and Pt(II)), undergoing metal reduction to M(0) and formation of NHC=O coupling azolone products (Figure 1a) [8]. In their study it is demonstrated that a base-mediated azolone NHC=O coupling reaction is integrated into the catalytic M/NHC systems and metal-free NHC derivatives are present in the catalytic mixture. A proposed mechanism of the revealed transformation includes NHC-OR reductive elimination, as implied by a series of mechanistic studies including ^18^OH^−^ labeling experiments. Similar decomposition pathways were reported on triazolylidene Cu(I) complexes, which decompose into mesoionic oxides in the presence of CsOH [16]. Almost no reports exist for a detailed description of NHC-Au(I) decomposition pathways [17].

Herein, we show how distinct NHC-Au(I) complexes undergo a controlled decomposition pathway towards the formation of the oxidized NHC=O azolone counterpart, together with the formation of naked eye-observable Au(0) nuggets. The NHC-Au(I) complexes studied under oxidative conditions cover various types, from typical IPrAuCl to novel (NHC-hemilabile)Au(I) complexes. Indeed, the ability of related triazolylidene ligands to bear pendant pyridine or pyrimidine groups for Au(I)/Au(III) redox processes has recently been studied [18]. We show that only typical NHC-Au(I) complexes such as IPr-Au-Cl and SIPr-Au-Cl are robust and remain intact after exposure to oxidants, and the gold complexes with NHC-hemilabile ligands or other NHC derivatives undergo the azolone/Au(0) nugget decomposition pathway. The origin of the O-atom in the azolone-products obtained is discussed. Remarkably, a straightforward and reliable chemical methodology to produce Au(0) nuggets from Au complexes is not available, and the main source of gold(0) nuggets are, like natural ores, hypogenic in origin.^19^ Native gold grains are usually small in size (10–500 mm), with a composition of 5 to <30% Ag, typical of hypogene gold, although Ag can be less than 1% depending on the goldfield of origin [19]. Supergene gold biomineralization has also been studied [20,21]. Recently, an Fe-based MOF/polymer composite (Fe-BTC/PpPDA) has been reported as a selective and efficient methodology to extract trace amounts of gold from water up to high purities (23.9 karats (K); >99%) (Figure 1b) [22]. Reduction of Au^3+^ to Au(0), mediated by the MOF-polymer composite, is proposed. On the other hand, recovery of Au(0) from ores or waste electronic materials entails stepwise tedious processes such as reduction with metallic Zn, HCl treatment, centrifugation and pyrolysis (Figure 1c) [23]. The decomposition to Au(0) observed in our systems could be used as a methodology to obtain metallic gold more easily, as we show that Au(0) nuggets of up to ~500 μm can be afforded in one step by treatment of NHC-Au(I) complexes with external oxidants.

## 2. Results and Discussion

### 2.1. Synthesis of Novel NHC-Au(I) Complexes (***1***–***4***)

**Figure 2:**
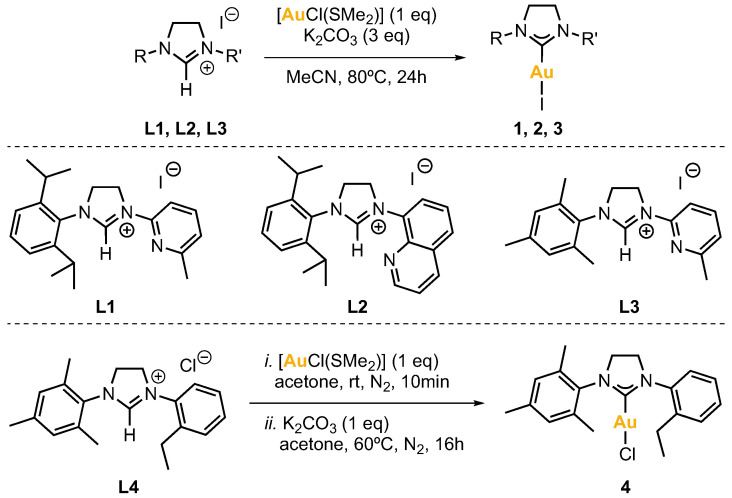
Synthesis of complexes **1**–**4** from the respective imidazolinium salt ligand precursors **L1**–**L4**.

The synthesis of imidazolinium salts **L1**, **L2**, **L3** and **L4** was achieved by standard procedures [24,25]. **L1**–**L3** contain a pendant pyridine or quinoline whereas **L4** contains a non-coordinating arene moiety (Figure 2).

The synthesis of NHC-Au(I) complexes was based on previously reported works [26,27]. Complexes **1**, **2** and **3** were obtained by reacting the corresponding imidazolinium salt **L1**, **L2** or **L3** (1 eq), chloro(dimethylsulfide)gold(I) (1 eq), and potassium carbonate (3 eq), in acetonitrile for 24 h at 80 °C. After this time, the reaction mixture was filtered over Celite^®^ and all volatiles were removed under vacuum. The product was purified by column chromatography. The fractions that contained the product were combined, and the solvent was removed under vacuum to afford the gold(I) complexes as solids. Complex **4** was obtained analogously from imidazolinium salt **L4** (Figure 2).

**Figure 3:**
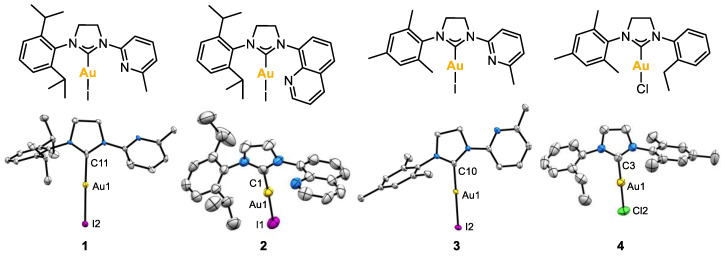
X-ray molecular structures of complexes **1**, **2**, **3** and **4** (ellipsoids set at 50% probability and H atoms removed for clarity). Selected bond distances (Å): for **1**, Au1-C11 2.007(13), Au1-I2 2.5580(10); for **2**, Au1-C1 1.981(9), Au1-I1 2.5467(8); for **3**, Au1-C10 2.000(3), Au1-I2 2.5516(2); for **4**, Au1-C3 1.966(6), Au1-Cl2 2.262(3). Selected angles (°): for **1**, C11-Au1-I2 177.4(4); for **2**, C1-Au1-I1 175.4(2); for **3**, C10-Au1-I2 178.38(8); for **4**, C3-Au1-Cl2 179.12(16).

X-ray molecular structures of complexes **1**, **2**, **3** and **4** were obtained (Figure 3). In complexes **1**–**3,** the pendant coordinating arm did not show any interaction with the metal center, and the linear coordination for the Au(I) center was confirmed in all cases, since the angle formed between the carbenic carbon, the gold center and the halide atom was close to 180° (for **1**, 177.4(4)°; for **2**, 175.4(2)°; for **3**, 178.38(8)°; for **4**, 179.12(16)°). No H-bonding network was observed with the non-coordinating pendant groups.

Complexes **1**–**4** were asymmetrical; therefore, the two methylenes of the imidazolidine ring appeared at different ^1^H NMR chemical shifts: for complexes **1** and **3,** the two -NC*H_2_*- appeared as multiplets at 4.54 and 3.90 ppm, respectively, and for complex **2**, at 4.68 and 4.08 ppm. For complex **4**, the two -NC*H_2_*- appeared at 4.22 and 3.96 ppm. On the other hand, the characteristic ^13^C NMR peak of the C_carbene_-Au appeared at 201.0, 203.7, 192.2 and 195.5 ppm for **1**, **2**, **3** and **4**, respectively.

### 2.2. Reactivity of Novel NHC-Au(I) Complexes (***1***–***4***) with External Oxidants

Complexes **1**–**3** were reacted with external oxidants in order to stabilize the NHC-Au(III) species, taking advantage of the pendant coordinating arms (pyridine-type for **1** and **3**, and quinoline-type for **2**). The external oxidants used were PhI(OAc)_2_, PhI(Cl)_2_, H_2_O_2_, XeF_2_, CH_3_CO_3_H under different solvents and temperatures (Table 1). However, the expected NHC-Au(III) species could not be stabilized in any case, and the mixture underwent a decomposition pathway involving the formation of a) the corresponding NHC=O azolones and b) Au(0) nuggets.

The formation of the NHC=O azolone products **L1^ox^**, **L2^ox^**, **L3^ox^**, **L4^ox^** was monitored by HRMS, and **L2^ox^** and **L3^ox^** were fully characterized by 1D and 2D NMR. In some reactions, the NHC-Au(I) complexes were treated with silver salts (as halide scavengers) and the oxidant. The aim of using a silver salt as additive was to promote the halide abstraction from the gold(I) starting complex and generate a reactive species that, hopefully, would evolve to a gold(III) complex in the presence of an oxidant, expecting the hemilabile N-donor arm to be coordinated to gold in the case of using complexes **1**–**3**. In addition, the halide abstraction could likely induce the formation of head-to-tail dimeric Au(I) species with the C^N ligand bridging two metals [18,28,29]. However, the synthesis of such dimeric species was not the focus of our interest.

When complex **1** was reacted with PhI(OAc)_2_, regardless whether AgOAc was added or not, Au(0) nuggets and azolones **L1^ox^** and **L1^ox^-I** were formed (entries 1,2). The azolones were detected by ESI-MS and, in the case of the reaction in entry 3, the **L1^ox^-I** product was isolated in 53% yield. When the oxidant was changed to H_2_O_2_, the outcome of the reaction in presence of AgOAc was mainly the starting complex **1**, whereas in the absence of AgOAc, **L1^ox^-I** could be detected by ESI-MS as well. **L1^ox^-I** was also detected in the reaction of **1** with PhI(Cl)_2_ at room temperature. Intriguingly, the formation of azolones that underwent a C-H iodination on the most activated position of the heteroaromatic system of the ligand was also observed. In this manner, products **L1^ox^-I** and **L2^ox^-I** were isolated and characterized. Indeed, the 2D NMR experiments revealed that the iodination occurred at the para position to the imidazolinone substituent (see Supplementary Material, Sections S5.7 and S5.9). The selective C-H halogenation at the para position to N-substituted positions and C5-halogenation of 8-aminoquinolines is a reactivity pattern that has already been reported [30,31,32,33,34]. Neither the reactions with H_2_O_2_ nor that with PhI(Cl)_2_ afforded Au(0) nuggets (entries 4–6). A blank experiment was conducted with complex **2**; without oxidant and additives, the complex was recovered after being heated at 100 °C overnight (entry 7). When using PhI(OAc)_2_, Au(0) nuggets were obtained in high yields (56–91%), and **L2^ox^** and **L2^ox^-I** products were detected by ESI-MS in all cases (entries 8–11). The isolation of both **L2^ox^** and **L2^ox^-I** azolones allowed their full characterization. When complex **2** was reacted with CH_3_CO_3_H as oxidant (entry 12), Au(0) nuggets were formed in moderate yield (34%) and NMR revealed the presence of complex **2** and **L2^ox^**.

The reaction of complex **3** with PhI(OAc)_2_ and AgOAc yielded Au(0) quantitatively, and azolone **L3^ox^** was isolated in 60% yield and characterized (entry 13). On the contrary, when the oxidant XeF_2_ was tested with complex **3** at room temperature, no Au(0) nuggets were formed (entry 14). However, the formation of azolone **L3^ox^** was detected by ESI-MS.

Complex **4** was employed to search for contrast with the complexes that contained a hemilabile pendant arm. The reaction with PhI(OAc)_2_ in DCM at room temperature did not provide Au(0) nuggets nor azolone, as the starting complex was recovered (entry 15). However, by heating at 100 °C for 5 h, the mixture decomposed, affording Au(0) nuggets in a 97% yield, and the corresponding **L4^ox^** azolone was detected by ESI-MS (entry 16). When **4** was reacted with PhI(OAc)_2_ in 1,2-DCE at 90 °C, Au(0) nuggets were obtained in a 32% yield (entry 17).

Interestingly, the commercial IPrAuCl was also reacted with PhI(OAc)_2_ under the same experimental conditions as **1**–**4,** and in this case, neither azolone product nor Au(0) nuggets were formed, and the complex was recovered after the reaction time (entries 18–19). Additionally, when the commercial saturated SIPrAuCl analog complex was reacted with PhI(OAc)_2_, a very low 11% conversion to Au(0) nuggets was obtained along with a 6% conversion to the corresponding azolone, as determined by NMR, according to the reported description of the azolone (entries 20,21) [35]. The stability of these well-known complexes is remarkable compared to that of **1**–**4**, showing that when a structural modification of the most typical NHC ligands, such as IPr and SIPr, is performed, a sharp change in reactivity occurs. Additionally, it is worth mentioning that there are examples of unsaturated NHC-Au(I) complexes bearing a hemilabile pyridine moiety that could be oxidized with PhI(Cl)_2_ to the corresponding NHC-Au(III) complexes without observing decomposition [36,37].

**Figure 4:**
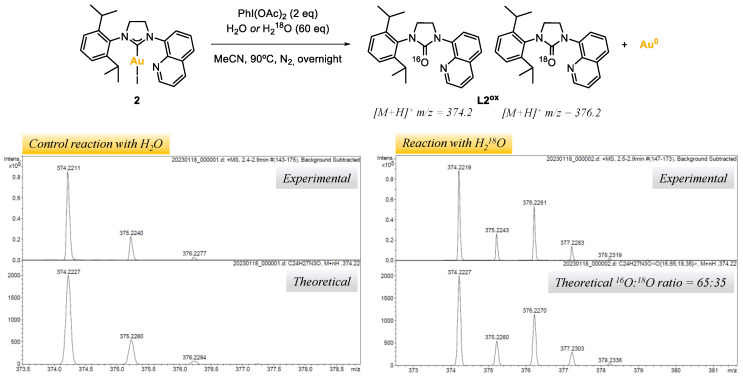
Reaction of complex **2** with PhI(OAc)_2_ (2 eq) and water or ^18^O-labeled water (60 eq), and peaks of **L2^ox^** obtained by ESI-HRMS(+).

The origin of the O-atom in azolones was investigated by reacting complex **2,** with PhI(OAc)_2_ as the oxidant and water as the additive, under nitrogen atmosphere (Table 1, entries 22,23, and Figure 4). In order to track the origin of the O-atom, a control reaction was carried out using distilled H_2_O, and another using 97% labeled ^18^O-water (see Supporting Material, Section S6). The reaction crudes were analyzed by ESI-HRMS. For the control experiment, a major peak at *m/z* = 374.2 was obtained, corresponding to the NHC=^16^O azolone **L2^ox^**, whereas for the reaction with H_2_^18^O two major peaks were obtained at *m/z* = 374.2 and *m/z* = 376.2, corresponding to the NHC=^16^O and NHC=^18^O azolones **L2^ox^**, respectively. According to the isotopic pattern of such peaks, a 35% of ^18^O incorporation was obtained, suggesting that the O-atom in the azolones obtained in the reaction outcomes may come from adventitious water. The low ^18^O incorporation from water suggests that an oxygen-transferring mechanism may exist, and that at some point of the mechanism, water might react with the oxidant to deliver a ^18^O-labeled reactive intermediate. Further investigations would be needed to elucidate the mechanism; at this stage, the alternative possibility of considering acetate to be the O-atom source cannot be ruled out [38].

### 2.3. Characterization and Reactivity of the Au(0) Nuggets

**Figure 5:**
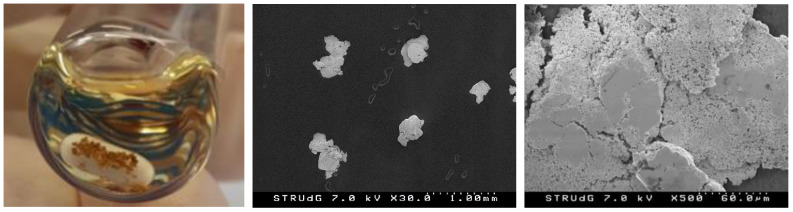
Reaction outcome with Au(0) precipitate (**left**), and SEM images of the isolated Au(0) nuggets at ×30 and ×500 (**middle** and **right**), from the reaction employing complex **1** (1 eq), AgOAc (1 eq) and PhI(OAc)_2_ (2 eq) in 1,2-DCE, at 90 °C overnight, under N_2_ atmosphere in the absence of light (Table 1, entry 1).

On the other hand, the formation of large Au(0) nuggets was naked-eye evident (Figure 5). The metallic gold grains were easily filtered/decanted and represented quantitative recovery (up to 90%) of all the Au(I) into Au(0) nuggets. The Au(0) nuggets were characterized by SEM and SEM-EDX, showing grains of 0.4–0.5 mm diameter mean size and purity up to 90% by EDX (only C and N impurities from the carbon tape support).

The Au(0) nuggets were tested as a heterogeneous catalyst for transformations typically catalyzed by gold complexes, such as Sonogashira, A3 and Glaser couplings [39,40,41,42,43]. None of these attempts resulted in effective coupling, which is in line with the macroscopic size of the Au(0) nuggets and the absence of more reactive Au(0) nanoparticles.

## 3. Materials and Methods

All reagents and solvents were purchased from Sigma Aldrich-Merck (Madrid, Spain), Fischer Scientific (Madrid, Spain), TCI (Windrush, Belgium) or Fluorophen (Glossip, United Kingdom) and were used without further purification. NMR spectra were recorded at 298 K unless otherwise specified, on Bruker spectrometers (Billerica, MA, USA)) operating at 400 MHz (^1^H NMR) and 101 MHz (^13^C{^1^H} NMR), or on a Varian Mercury 200 MHz spectrometer (Palo Alto, CA, USA), and referenced to residual solvent (δ in ppm and J in hertz). High resolution mass spectra (HRMS) were recorded on a Bruker Microsoft-Q IITM or a QTOF maxis Impact (Bruker) spectrometer using ESI source at Servais Tècnics de Recerca, University of Girona, or at the National and Kapodistrian University of Athens. For reactions carried out under inert atmosphere, a N_2_ drybox with O_2_ and H_2_O concentrations <1 ppm was employed, or standard Schlenk techniques were followed. SEM images of the Au(0) nuggets were carried out with a scanning electron microscope FE-SEM Hitachi S-4100 (Hitachi, Chiyoda City, Japan). Digital images were collected and processed by Quarz PCI program. SEM-EDX analysis was performed with a scanning electron microscope Zeiss DSM 960 Germany (EDX Bruker, Quantax Esprit Spectrometer SVE III, Billerica, MA, USA).

**Synthesis of complexes 1**–**3**. For the synthesis of NHC-Au(I) complexes **1**, **2** and **3**, the corresponding imidazolinium salt **L1**, **L2** or **L3** (1 eq), chloro(dimethylsulfide)gold(I) (1 eq), and potassium carbonate (3 eq), were reacted in acetonitrile for 24 h at 80 °C. After this time, the reaction mixture was filtered over Celite^®^ and all volatiles were removed under vacuum. The product was purified by column chromatography. The fractions that contained the product were combined, and the solvent was removed under vacuum to afford the gold(I) complexes as solids.

**Complex 1.** The imidazolinium iodide salt **L1** (102.4 mg, 0.23 mmol, 1.0 eq.), K_2_CO_3_ (126.7 mg, 0.92 mmol, 4.0 eq.) and [AuCl(SMe_2_)] (87.2 mg, 0.30 mmol, 1.3 eq.) were reacted in acetonitrile (1 mL). The product was purified by column chromatography using DCM:hexane (4:1). By slow diffusion of pentane into a concentrated solution of complex **1** in chloroform, pale yellow crystals suitable for X-ray diffraction analysis were obtained (59.3 mg, 40% yield). ^1^H NMR (400 MHz, CDCl_3_) δ 8.58 (d, *J* = 8.3 Hz, 1H, C*H*_py_), 7.63 (t, *J* = 7.8 Hz, 1H, C*H*_py_), 7.43 (t, *J* = 7.8 Hz, 1H, C*H*_Ar_), 7.24 (d, *J* = 7.8 Hz, 2H, C*H*_Ar_), 7.02 (d, *J* = 7.5 Hz, 1H, C*H*_py_), 4.59–4.52 (m, 2H, NC*H*_2_), 3.95–3.87 (m, 2H, NC*H*_2_), 2.97 (hept, *J* = 6.8 Hz, 2H, C*H*(CH_3_)_2_), 2.53 (s, 3H, C*H*_3_), 1.39 (d, *J* = 6.8 Hz, 6H, CH(C*H*_3_)_2_), 1.26 (d, *J* = 6.9 Hz, 6H, CH(C*H*_3_)_2_). ^13^C{^1^H} NMR (101 MHz, CDCl_3_) δ 201.0 (C_carbene_-Au), 157.3 (*C*_py_), 151.9 (*C*_py_), 146.2 (*C*_Ar_, 2C), 138.3 (*C*H_py_), 134.7 (*C*_Ar_), 130.3 (*C*H_Ar_), 124.8 (*C*H_Ar_, 2C), 120.5 (*C*H_py_), 111.4 (*C*H_py_), 53.1 (N*C*H_2_), 48.7 (N*C*H_2_), 28.7 (*C*H(CH_3_)_2_, 2C), 25.0 (CH(*C*H_3_)_2_, 2C), 24.6 (CH(*C*H_3_)_2_, 2C), 24.4 (*C*H_3_). HRMS (ESI+): calcd. for C_21_H_27_AuIN_3_ [M + H]^+^: *m/z* 646.0988; found: *m/z* 646.0984; [M + Na]^+^: *m/z* 668.0807; found: 668.0840; [2M − I]^+^: *m/z* 1163.2780; found: 1163.2804.

**Complex 2.** The imidazolinium iodide salt **L2** (180.0 mg, 0.37 mmol, 1.0 eq.), K_2_CO_3_ (261.2 mg, 1.89 mmol, 5.1 eq.) and [AuCl(SMe_2_)] (139.1 mg, 0.47 mmol, 1.3 eq.) were reacted in acetonitrile (2 mL). After the filtration over Celite^®^, the product was purified by filtering the residue over a pad of silica using DCM. The solvent was removed to obtain a yellow solid. It was washed with hexane and diethyl ether to afford complex **2** as a white solid (105.8 mg, 42% yield). ^1^H NMR (400 MHz, CDCl_3_) δ 8.96 (dd, *J* = 4.2, 1.7 Hz, 1H, C*H*_Quin_), 8.23 (dd, *J* = 8.3, 1.7 Hz, 1H, C*H*_Quin_), 8.10 (dd, *J* = 7.3, 1.4 Hz, 1H, C*H*_Quin_), 7.87 (dd, *J* = 8.2, 1.4 Hz, 1H, C*H*_Quin_), 7.60 (dd, *J* = 8.3, 7.4 Hz, 1H, C*H*_Quin_), 7.50 (dd, *J* = 8.3, 4.2 Hz, 1H, C*H*_Quin_), 7.42 (t, *J* = 7.8 Hz, 1H, C*H*_Ar_), 7.25 (d, *J* = 7.8 Hz, 2H, C*H*_Ar_), 4.72–4.64 (m, 2H, NC*H*_2_), 4.12–4.04 (m, 2H, NC*H*_2_), 3.23 (hept, *J* = 6.9 Hz, 2H, C*H*(CH_3_)_2_), 1.43 (d, *J* = 6.8 Hz, 6H, CH(C*H*_3_)_2_), 1.39 (d, *J* = 6.8 Hz, 6H, CH(C*H*_3_)_2_). **^13^**C{^1^H} NMR (101 MHz, CDCl_3_) δ 203.7 (C_carbene_-Au), 150.4 (*C*H_Quin_), 146.8 (*C*_Ar_, 2C), 144.1 (*C*_Quin_), 137.6 (*C*_Quin_), 136.6 (*C*H_Quin_), 134.6 (*C*_Ar_), 130.0 (*C*H_Ar_), 129.6 (*C*_Quin_), 128.8 (*C*H_Quin_), 128.3 (*C*H_Quin_), 126.4 (*C*H_Quin_), 124.7 (*C*H_Ar_, 2C), 122.0 (*C*H_Quin_), 54.4 (N*C*H_2_), 53.0 (N*C*H_2_), 28.7 (*C*H(CH_3_)_2_, 2C), 25.3 (CH(*C*H_3_)_2_, 2C), 24.6 (CH(*C*H_3_)_2_, 2C). HRMS (ESI+): calcd. for C_24_H_27_AuIN_3_ [M + H]^+^: *m/z* 682.0988; found: *m/z* 682.0974; [2M − I]^+^: *m/z* 1235.2780; found: 1235.2746.

**Complex 3.** The imidazolinium iodide salt **L3** (67.5 mg, 0.17 mmol, 1.0 eq.), K_2_CO_3_ (91.1 mg, 0.66 mmol, 4.0 eq.) and [AuCl(SMe_2_)] (63.8 mg, 0.22 mmol, 1.3 eq.) were reacted in acetonitrile (1 mL). The product was purified by column chromatography using DCM. By slow diffusion of pentane into a concentrated solution of complex **3** in chloroform, crystals suitable for X-ray diffraction analysis were obtained (32.9 mg, 33% yield). ^1^H NMR (400 MHz, CDCl_3_) δ 8.54 (d, *J* = 8.2 Hz, 1H, C*H*_py_), 7.62 (t, *J* = 7.9 Hz, 1H, C*H*_py_), 7.02 (d, *J* = 7.5 Hz, 1H, C*H*_py_), 6.94 (s, 2H, C*H*_Ar_), 4.57–4.48 (m, 2H, NC*H*_2_), 3.94–3.85 (m, 2H, NC*H*_2_), 2.52 (s, 3H, C*H*_3py_), 2.30 (s, 3H, C*H*_3Ar_), 2.27 (s, 6H, C*H*_3Ar_). ^13^C{^1^H} NMR (101 MHz, CDCl_3_) δ 192.2 (C_carbene_-Au), 157.3 (*C*_py_), 151.9 (*C*_py_), 139.3 (*C*_Ar_), 138.3 (*C*H_py_), 135.5 (*C*_Ar_), 135.1 (*C*_Ar_, 2C), 130.0 (*C*H_Ar_, 2C), 120.6 (*C*H_py_), 112.0 (*C*H_py_), 50.3 (N*C*H_2_), 48.8 (N*C*H_2_), 24.3 (*C*H_3py_), 21.2 (*C*H_3Ar_), 18.2 (*C*H_3Ar_, 2C). HRMS (ESI+): calcd. for C_18_H_21_AuIN_3_ [M + Na]^+^: *m/z* 626.0338; found: *m/z* 626.0336; [(C_18_H_21_N_3_)_2_Au]^+^: *m/z* 755.3131; found: 755.3150; [2M − I]^+^: *m/z* 1079.1841; found: 1079.1830.

**Complex 4.** For the synthesis of NHC-Au(I) complex **4**, imidazolinium salt **L4** (60.1 mg, 0.18 mmol, 1.0 eq) and chloro(dimethylsulfide)gold(I) (54.8 mg, 0.19 mmol, 1.0 eq) were mixed in acetone (0.6 mL), under nitrogen atmosphere, and stirred at room temperature for 10 min. Then, potassium carbonate (27.6 mg, 0.20 mmol, 1.1 eq) was added and the mixture was stirred and heated at 60 °C for 16 h. After this time, the reaction mixture was cooled down to room temperature, then the solvent was removed under reduced pressure, and DCM was added. The mixture was filtered over a pad of silica, which was washed with more DCM, and the resulting solution was concentrated to the minimal volume. Next, pentane was added to precipitate the desired product. It was washed with more pentane, and it was dried under vacuum. Complex **4** was obtained as a white solid (75.5 mg, 67% yield). ^1^H NMR (400 MHz, CDCl_3_) δ 7.40–7.24 (m, 4H, C*H*_Ar_), 6.94 (d, *J* = 0.7 Hz, 2H, C*H*_Ar_), 4.11 (t, *J* = 9.6 Hz, 2H, NC*H*_2_), 3.96 (td, *J* = 10.4, 1.7 Hz, 2H, NC*H*_2_), 2.76 (q, *J* = 7.6 Hz, 2H, C*H*_2_CH_3_), 2.31 (s, 6H, C*H*_3_), 2.30 (s, 3H, C*H*_3_), 1.35 (t, *J* = 7.6 Hz, 3H, CH_2_C*H*_3_). ^13^C{^1^H} NMR (101 MHz, CDCl_3_) δ 195.0 (C_carbene_-Au), 141.2 (*C*_Ar_), 139.1 (*C*_Ar_), 138.4 (*C*_Ar_), 135.6 (*C*_Ar_, 2C), 134.8 (*C*_Ar_), 129.9 (*C*H_Ar_, 2C), 129.9 (*C*H_Ar_), 129.5 (*C*H_Ar_), 128.4 (*C*H_Ar_), 127.5 (*C*H_Ar_), 53.3 (N*C*H_2_), 51.0 (N*C*H_2_), 24.4 (*C*H_2_CH_3_), 21.2 (*C*H_3_), 18.1 (*C*H_3_, 2C), 15.0 (CH_2_*C*H_3_). HRMS (ESI+): calcd. for C_20_H_24_AuClN_2_ [M + Na]^+^: *m/z* 547.1186; found: *m/z* 547.1181; [2M + Na]^+^: *m/z* 1071.2479; found: 1071.2438; [2M − Cl]^+^: *m/z* 1013.2899; found: 1013.2865.

**Compound L1^ox^-I.**^1^H NMR (400 MHz, 228K, CDCl_3_) δ 7.91–7.83 (m, 2H, C*H*_py_), 7.38 (t, *J* = 7.7 Hz, 1H, C*H*_Ar_), 7.23 (d, *J* = 7.6 Hz, 2H, C*H*_Ar_), 4.23 (t, *J* = 8.2 Hz, 2H, NC*H*_2_), 3.71 (t, *J* = 8.2 Hz, 2H, NC*H*_2_), 2.98 (hept, *J* = 6.8 Hz, 2H, C*H*(CH_3_)_2_), 2.63 (s, 3H, C*H*_3_), 1.23 (d, *J* = 6.9 Hz, 6H, CH(C*H*_3_)_2_), 1.20 (d, *J* = 6.6 Hz, 6H, CH(C*H*_3_)_2_). ^13^C{^1^H} NMR (101 MHz, CDCl_3_) δ 157.7 (*C*_py_), 156.2 (C=O), 152.1 (*C*_py_), 148.0 (*C*_Ar_, 2C), 147.3 (*C*H_py_), 132.8 (*C*_Ar_), 129.2 (*C*H_Ar_), 124.3 (*C*H_Ar_, 2C), 112.2 (*C*H_py_), 86.2 (*C*_py_-I), 45.7 (N*C*H_2_), 41.7 (N*C*H_2_), 28.9 (*C*H(CH_3_)_2_, 2C), 28.8 (*C*H_3_), 24.6 (CH(*C*H_3_)_2_, 2C), 24.4 (CH(*C*H_3_)_2_, 2C). HRMS (ESI+): calcd for C_21_H_26_IN_3_O [M + H]^+^: *m/z* 464.1193; found: *m/z* 464.1193; [M + Na]^+^: *m/z* 486.1013; found: *m/z* 486.1012; [2M + Na]^+^: *m/z* 949.2133; found: *m/z* 949.2141.

**Compound L2^ox^.**^1^H NMR (400 MHz, CDCl_3_) δ 8.93 (dd, *J* = 4.2, 1.8 Hz, 1H, C*H*_Ar_), 8.17 (dd, *J* = 8.3, 1.8 Hz, 1H, C*H*_Ar_), 7.93 (dd, *J* = 7.5, 1.4 Hz, 1H, C*H*_Ar_), 7.71 (dd, *J* = 8.2, 1.4 Hz, 1H, C*H*_Ar_), 7.56 (dd, *J* = 8.2, 7.5 Hz, 1H, C*H*_Ar_), 7.42 (dd, *J* = 8.3, 4.1 Hz, 1H, C*H*_Ar_), 7.35 (dd, *J* = 8.3, 7.1 Hz, 1H, C*H*_Ar_), 7.23 (d, *J* = 7.7 Hz, 2H, C*H*_Ar_), 4.45–4.40 (m, 2H, NC*H*_2_), 3.90–3.84 (m, 2H, NC*H*_2_), 3.32 (hept, *J* = 6.9 Hz, 2H, C*H*(CH_3_)_2_), 1.32 (d, *J* = 6.9 Hz, 6H, CH(C*H*_3_)_2_), 1.30 (d, *J* = 6.8 Hz, 6H, CH(C*H*_3_)_2_). ^13^C{^1^H} NMR (101 MHz, CDCl_3_) δ 159.5 (C=O), 149.3 (*C*H_Quin_), 148.5 (*C*_Ar_, 2C), 144.3 (*C*_Quin_), 138.3 (*C*_Quin_), 136.5 (*C*H_Quin_), 133.8 (*C*_Ar_), 129.7 (*C*_Quin_), 128.8 (*C*H_Ar_), 127.5 (*C*H_Quin_), 126.7 (*C*H_Quin_), 126.1 (*C*H_Quin_), 124.2 (*C*H_Ar_, 2C), 121.3 (*C*H_Quin_), 47.4 (N*C*H_2_), 47.0 (N*C*H_2_), 28.8 (*C*H(CH_3_)_2_, 2C), 24.8 (CH(*C*H_3_)_2_, 2C), 24.5 (CH(*C*H_3_)_2_, 2C). HRMS (ESI+): calcd. for C_24_H_27_N_3_O [M + H]^+^: *m/z* 374.2227; found: *m/z* 374.2211; [M + Na]^+^: *m/z* 396.2046; found: 396.2036; [2M + Na]^+^: *m/z* 769.4200; found: *m/z* 769.4164.

**Compound L2^ox^-I.**^1^H NMR (400 MHz, CDCl_3_) δ 8.88 (dd, *J* = 4.1, 1.6 Hz, 1H, C*H*_Quin_), 8.39 (dd, *J* = 8.5, 1.6 Hz, 1H, C*H*_Quin_), 8.13 (d, *J* = 8.1 Hz, 1H, C*H*_Quin_), 7.69 (d, *J* = 8.0 Hz, 1H, C*H*_Quin_), 7.49 (dd, *J* = 8.6, 4.1 Hz, 1H, C*H*_Quin_), 7.35 (dd, *J* = 8.3, 7.1 Hz, 1H, C*H*_Ar_), 7.23 (d, *J* = 7.4 Hz, 2H, C*H*_Ar_), 4.46–4.39 (m, 2H, NC*H*_2_), 3.89–3.83 (m, 2H, NC*H*_2_), 3.28 (hept, *J* = 6.9 Hz, 2H, C*H*(CH_3_)_2_), 1.32 (d, *J* = 6.9 Hz, 6H, CH(C*H*_3_)_2_), 1.29 (d, *J* = 6.8 Hz, 6H, CH(C*H*_3_)_2_). ^13^C{^1^H} NMR (101 MHz, CDCl_3_) δ 159.2 (C=O), 149.9 (*C*H_Quin_), 148.4 (*C*_Ar_, 2C), 144.8 (*C*_Quin_), 140.9 (*C*H_Quin_), 139.4 (*C*_Quin_), 137.7 (*C*H_Quin_), 133.5 (*C*_Ar_), 131.2 (*C*_Quin_), 129.0 (*C*H_Ar_), 128.5 (*C*H_Quin_), 124.2 (*C*H_Ar_, 2C), 122.9 (*C*H_Quin_), 95.3 (*C*_Quin_-I), 47.4 (N*C*H_2_), 47.0 (N*C*H_2_), 28.8 (*C*H(CH_3_)_2_, 2C), 24.8 (CH(*C*H_3_)_2_, 2C), 24.5 (CH(*C*H_3_)_2_, 2C). HRMS (ESI+): calcd. for C_24_H_26_IN_3_O [M + H]^+^: *m/z* 500.1193; found: *m/z* 500.1184; [M + Na]^+^: *m/z* 522.1013; found: 522.0999.

**Compound L3^ox^.**^1^H NMR (400 MHz, CDCl_3_) δ 8.11 (d, *J* = 8.4 Hz, 1H, C*H*_py_), 7.51 (t, *J* = 7.9 Hz, 1H, C*H*_py_), 6.93 (s, 2H, C*H*_Ar_), 6.78 (d, *J* = 7.3 Hz, 1H, C*H*_py_), 4.31–4.23 (m, 2H, NC*H*_2_), 3.74–3.67 (m, 2H, NC*H*_2_), 2.47 (s, 3H, C*H*_3py_), 2.29 (s, 3H, C*H*_3Ar_), 2.25 (s, 6H, C*H*_3Ar_). ^13^C{^1^H} NMR (101 MHz, CDCl_3_) δ 156.3 (*C*_py_), 155.9 (C=O), 152.4 (*C*_py_), 138.0 (*C*_Ar_), 137.7 (*C*H_py_), 137.0 (*C*_Ar_, 2C), 133.1 (*C*_Ar_), 129.5 (*C*H_Ar_, 2C), 116.8 (*C*H_py_), 109.9 (*C*H_py_), 43.2 (N*C*H_2_), 42.0 (N*C*H_2_), 24.5 (*C*H_3py_), 21.1 (*C*H_3Ar_), 18.0 (*C*H_3Ar_, 2C). HRMS (ESI+): calcd. for C_18_H_21_N_3_O [M + H]^+^: *m/z* 296.1757; found: *m/z* 296.1774; [M + Na]^+^: *m/z* 318.1577; found: 318.1595; [2M + Na]^+^: *m/z* 613.3261; found: *m/z* 613.3278.

## 4. Conclusions

In summary, novel NHC-Au(I) complexes (**1**–**4**) were synthesized and thoroughly characterized, and their reactivity in front external oxidants was analyzed. In contrast to the stability shown by commercial IPr- and SIPr-Au(I) complexes, complexes **1**–**4** underwent a controlled decomposition pathway to form oxidized NHC=O azolones as the main organic product, and quantitative conversion of all the Au(I) contained in complexes into macroscopic Au(0) nuggets (~0.4–0.5 mm). Azolones and M(0) have previously been described as decomposition products of NHC-metal complexes (M = Pd(II), Pt(II), Ni(II)), and we show herein a singular case of gold transforming this decomposition pathway, representing a good strategy to recover gold nuggets of high purity from soluble gold species (as NHC-Au(I)).

## Figures and Tables

**Table 1:** Reactivity of gold(I) complexes towards the formation of gold(0) nuggets and azolones (**Lx^ox^**).

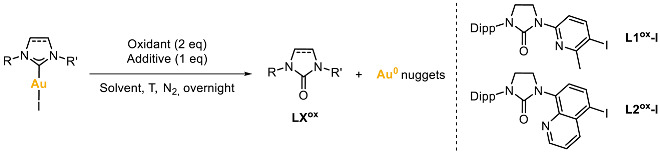							
Entry	Complex	Oxidant	Additive	Solvent	T (°C)	Yield % Au(0)	NHC=O (yield %) *
1	**1**	PhI(OAc)_2_	AgOAc	1,2-DCE	90	75	Detected MS
2	**1**	PhI(OAc)_2_	-	1,2-DCE	90	46	Detected MS
3	**1**	PhI(OAc)_2_	-	MeCN	90	60	**L1^ox^-I** (53)
4	**1**	H_2_O_2_	AgOAc	1,2-DCE	90	0	0
5	**1**	H_2_O_2_	-	1,2-DCE	90	0	Detected MS
6	**1**	PhI(Cl)_2_	-	DCM	rt	0	Detected MS
7	**2**	-	-	1,2-DCE	100	0	0
8	**2**	PhI(OAc)_2_	AgOAc	1,2-DCE	90	85	Detected MS
9	**2**	PhI(OAc)_2_	-	1,2-DCE	90	90	**L2^ox^** (22) and **L2^ox^-I** (17)
10	**2**	PhI(OAc)_2_	AgOAc	DCM	70	91	Detected MS
11	**2**	PhI(OAc)_2_	-	MeCN	90	56	**L2^ox^** (34)
12	**2**	CH_3_CO_3_H	-	1,2-DCE	90	34	Detected NMR
13	**3**	PhI(OAc)_2_	AgOAc	1,2-DCE	90	>99	**L3^ox^** (60)
14	**3**	XeF_2_	-	CDCl_3_	rt	0	Detected MS
15	**4**	PhI(OAc)_2_	-	DCM	rt	0	0
16 ^a^	**4**	PhI(OAc)_2_	-	DCM	100	97	Detected MS
17	**4**	PhI(OAc)_2_	-	1,2-DCE	90	32	Detected MS
18	IPrAuCl	PhI(OAc)_2_	AgOAc	1,2-DCE	90	0	0
19	IPrAuCl	PhI(OAc)_2_	-	1,2-DCE	90	0	0
20	SIPrAuCl	PhI(OAc)	AgOAc	1,2-DCE	90	0	0
21	SIPrAuCl	PhI(OAc)	-	1,2-DCE	90	11	6
22 ^b^	**2**	PhI(OAc)_2_	H_2_O	MeCN	90	88	**L2^ox^** (48)
23 ^b^	**2**	PhI(OAc)_2_	H_2_^18^O	MeCN	90	97	**L2^ox^** (41)

* Isolated yield; ^a^ 5 h of reaction; ^b^ 60 equivalents of additive.

## Data Availability

Data is contained within the article or supplementary material.

## Supplementary Materials

The following supporting information can be downloaded at: https://www.mdpi.com/article/10.3390/molecules28052302/s1, Figure S1–S80, Table S1–S6, Schemes S1–S8. CCDC 2238757 (**1**), 2238754 (**2**), 2238755 (**3**), 2238756, (**4**) contain the supplementary crystallographic data for this paper [44].

## Sample Availability

Samples of the compounds **1**–**4** are available from the authors.
