# Supplementary material for: Novel NHC-Based Au(I) Complexes as Precursors of Highly Pure Au(0) Nuggets under Oxidative Conditions
Source: Molecules. 2023 Mar 1;28(5):2302. doi: 10.3390/molecules28052302 (PMC10005697; doi:10.3390/molecules28052302)
Supplement: Supplementary file 1 [file molecules-28-02302-s001.zip › molecules-2226132-supplementary.pdf]

SUPPORTING INFORMATION

Novel NHC-based Au(I) complexes as precursors of highly pure Au(0) nuggets under oxidative conditions

Pau Font,[†] Nikolaos V. Tzouras,[‡] Argyro T. Papastavrou,[‡] Georgios C. Vougioukalakis,[‡,*] Xavi Ribas[†,*]

Table of Contents

S1. General considerations 3

S2. Synthesis of imidazolinium salts 3

S3. Synthesis of gold(I) complexes 5

S3.1. General procedure for the synthesis of complexes 1, 2 and 3 5

S3.2. Synthesis of complex 4 7

S4. Reactivity and products 8

S5. NMR, HRMS-ESI and IR spectra 10

S5.1. Compound L1 10

S5.2. Compound L3 11

S5.3. Complex 1 13

S5.4. Complex 2 17

S5.5. Complex 3 20

S5.6. Complex 4 23

S5.7. Compound $L1^{ox}$*-I* 27

S5.8. Compound $L2^{ox}$ 32

S5.9. Compound $L2^{ox}$*-I* 35

S5.10. Compound $L3^{ox}$ 38

S6. Experiments using water and ^{18}**O-labeled water as additive** 42

S7. X-Ray structures and crystallographic data 44

S7.1. Complex 1 44

S7.2. Complex 2 45

S7.3. Complex 3 47

S7.4. Complex 4 48

S8. SEM-EDX characterization of Au(0) macroaggregates 50

S8.1. SEM images 50

S8.2. SEM-EDX analysis 53

S9. Heterogeneous Au(0) catalysis attempts 57

S10. References 58

S1. General considerations

All reagents and solvents were purchased from Sigma Aldrich, Fischer Scientific, TCI or Fluorochem and were used without further purification. NMR spectra were recorded at 298K, unless otherwise specified, on Bruker spectrometers operating at 400 MHz (^1H NMR) and 101 MHz ($^{13}C\{^1H\}$ NMR), or on a Varian Mercury 200 MHz spectrometer, and referenced to residual solvents (δ in ppm and J in hertz). High resolution mass spectra (HRMS) were recorded on a Bruker MicroTOF-Q IITM or a QTOF maxis Impact (Bruker) spectrometer using ESI source at Serveis Tècnics de Recerca, University of Girona, or at the National and Kapodistrian University of Athens. For reactions carried out under an inert atmosphere, a N_2 drybox with O_2 and H_2O concentrations <1 ppm was employed, or standard Schleck techniques were followed. SEM images of the Au(0) nuggets were carried out with a scanning electron microscope (FE-SEM Hitachi, Japan, S-4100). Digital images were collected and processed by the Quarz PCI program. SEM-EDX analysis was performed with a scanning electron microscope (Zeiss DSM 960 Germany, EDX Bruker, Quantax Esprit Spectrometer SVE III).

S2. Synthesis of imidazolinium salts

Imidazolinium salts **L2** and **L4** were prepared according to the procedures outlined in the literature.[1,2] Imidazolinium salts **L1** and **L3** were prepared according to the procedures[3] outlined in the literature with the modifications illustrated in Scheme S1.

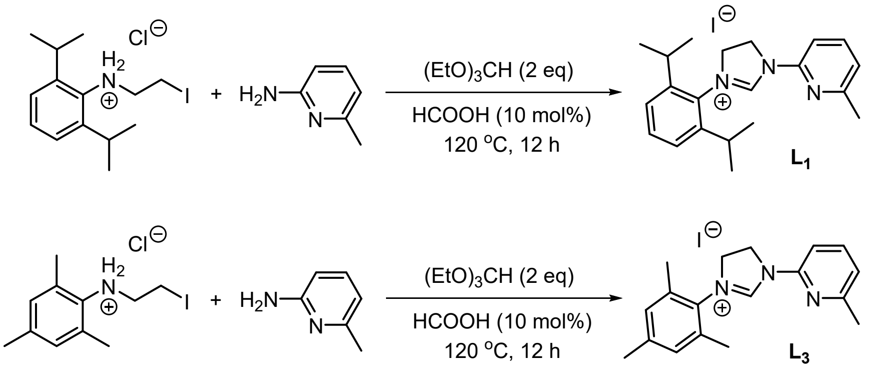

Scheme S1. General reaction conditions for the synthesis of imidazolinium salts **L1** and **L3**.

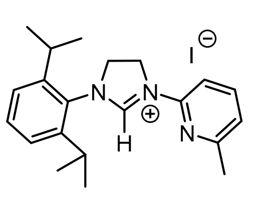

L1. To a mixture of N-(2-iodoethyl)-2,6-diisopropylbenzenaminium chloride (1.4 g, 3.81 mmol, prepared as described in the literature[3]), 6-methylpyridin-2-amine (0.41 g, 3.81 mmol) and triethylorthoformate (1.27 mL, 7.62 mmol) in a flame-dried pressure tube with a screw-top cap and a magnetic stirring bar, a catalytic amount of formic acid (14 μL, 10 mol%) was added via micropipette under argon, and the mixture was heated to 120 ºC in a pre-heated oil bath for 12 h. Toluene was added to the resulting dark mixture, and since no precipitation was observed, the mixture was transferred to a larger vessel and the solvent was removed. The mixture was dried for a prolonged period of time under vacuum on a rotary evaporator, as well as under high vacuum to remove the excess of triethylorthoformate and other volatile impurities

inhibiting precipitation. The resulting viscous red/brown residue was dissolved in a minimal amount of pure acetone and trituration with diethyl ether, leading to the precipitation of a solid. The solid was collected on a frit, washed with toluene (2x5 mL) and diethyl ether (2x5 mL), and then dried under vacuum. This process was repeated once on the supernatant, affording the product as a light brown powder in 22% yield (375 mg, 0.83 mmol). **^{1}H NMR** (200 MHz, $CDCl_3$) δ 9.23 (s, 1H, N(C*H*)N), 7.81 (t, *J* = 7.8 Hz, 1H, CH_{py}), 7.56 – 7.44 (m, 1H, CH_{Ar}), 7.38 (d, *J* = 8.3 Hz, 1H, CH_{py}), 7.32 (d, *J* = 7.5 Hz, 2H, CH_{Ar}), 7.15 (d, *J* = 7.7 Hz, 1H, CH_{py}), 5.20 (t, *J* = 11.1 Hz, 2H, NCH_2), 4.91 – 4.62 (m, 2H, NCH_2), 3.12 – 2.96 (m, 2H, C*H*(CH_3)$_2$), 2.48 (s, 3H, CH_3), 1.36 (d, *J* = 6.8 Hz, 6H, CH(CH_3)$_2$), 1.30 (d, *J* = 6.8 Hz, 6H, CH(CH_3)$_2$). **^{13}C{^{1}H} NMR** (50 MHz, $CDCl_3$) δ 158.7, 153.6, 146.7, 146.2, 140.2, 131.7, 129.7, 125.2, 122.7, 109.3, 55.7 (N*C*H_2), 48.7 (N*C*H_2), 28.9 (*C*H(CH_3)$_2$), 25.2 (CH(*C*H_3)$_2$), 24.4 (CH(*C*H_3)$_2$), 24.2 (*C*H_3). **HRMS (ESI+)**: calcd for $C_{21}H_{28}N_3^+$ $[M]^+$: *m/z* 322.2278; found: *m/z* 322.2299.

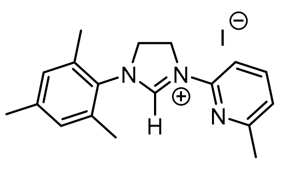

L3. To a mixture of N-(2-iodoethyl)-2,4,6-trimethylbenzenaminium chloride (977 mg, 3.0 mmol, prepared as described in the literature[3]), 6-methylpyridin-2-amine (324 mg, 3.0 mmol) and triethylorthoformate (1.0 mL, 6.0 mmol) in a flame-dried pressure tube with a screw-top cap and a magnetic stirring bar, a catalytic amount of formic acid (10 mol%) was added via micropipette under argon, and the mixture was heated to 120 ºC in a pre-heated oil bath for 12 h. Toluene was added to the resulting mixture and a precipitate formed. The solid was collected on a frit, washed with toluene (2x5 mL) and diethyl ether (2x5 mL) and then dried under vacuum. Gradient column chromatography on silica gel using DCM/MeOH 95/5-90/10 afforded the product as a white powder in an 8% yield (102 mg, 0.25 mmol). **^{1}H NMR** (200 MHz, $CDCl_3$) δ 9.68 (s, 1H, N(C*H*)N), 7.89 – 7.72 (m, 1H, CH_{py}), 7.54 (d, *J* = 8.2 Hz, 1H, CH_{py}), 7.13 (d, *J* = 7.4 Hz, 1H, CH_{py}), 7.01 (s, 2H, CH_{Ar}), 5.34 – 4.87 (m, 2H, NCH_2), 4.75 – 4.55 (m, 2H, NCH_2), 2.50 (s, 3H, CH_3), 2.41 (s, 6H, 2xCH_3), 2.33 (s, 3H, CH_3). **^{13}C{^{1}H} NMR** (50 MHz, $CDCl_3$) δ 158.4, 153.9, 146.5, 140.7, 139.7, 134.7, 130.1, 129.9, 122.2, 108.8, 53.1 (N*C*H_2), 48.1 (N*C*H_2), 24.0 (*C*H_3), 20.9 (*C*H_3), 18.5 (*C*H_3, 2C). **HRMS (ESI+)**: calcd for $C_{21}H_{28}N_3^+$ $[M]^+$: *m/z* 280.1808; found: *m/z* 280.1841.

S3. Synthesis of gold(I) complexes

S3.1. General procedure for the synthesis of complexes 1, 2 and 3

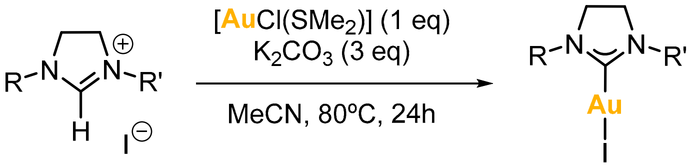

L1 R = Dipp, R' = 2-(6-methylpyridinyl)
L2 R = Dipp, R' = 8-quinolinyl
L3 R = Mes, R' = 2-(6-methylpyridinyl)

Scheme S2. General reaction conditions for the synthesis of complexes **1**, **2** and **3**.

For the synthesis of NHC-Au(I) complexes **1**, **2** and **3**, the corresponding imidazolinium salt **L1**, **L2** or **L3** (1 eq), chloro(dimethylsulfide)gold(I) (1 eq), and potassium carbonate (3 eq), were reacted in acetonitrile for 24 hours at 80ºC. After this time, the reaction mixture was filtered over Celite® and all volatiles were removed under vacuum. The product was purified by column chromatography. The fractions that contained the product were combined, and the solvent was removed under vacuum to afford the gold(I) complexes as solids.

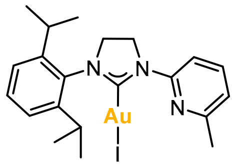

Complex 1. The imidazolinium iodide salt **L1** (102.4 mg, 0.23 mmol, 1.0 eq.), K_2CO_3 (126.7 mg, 0.92 mmol, 4.0 eq.) and [AuCl(SMe$_2$)] (87.2 mg, 0.30 mmol, 1.3 eq.) were reacted in acetonitrile (1 mL). The product was purified by column chromatography using DCM:hexane (4:1). By slow diffusion of pentane into a concentrated solution of complex **1** in chloroform, pale yellow crystals suitable for X-ray diffraction analysis were obtained (59.3 mg, 40% yield). **^{1}H NMR** (400 MHz, $CDCl_3$) δ 8.58 (d, J = 8.3 Hz, 1H, CH_{py}), 7.63 (t, J = 7.8 Hz, 1H, CH_{py}), 7.43 (t, J = 7.8 Hz, 1H, CH_{Ar}), 7.24 (d, J = 7.8 Hz, 2H, CH_{Ar}), 7.02 (d, J = 7.5 Hz, 1H, CH_{py}), 4.59 – 4.52 (m, 2H, NCH_2), 3.95 – 3.87 (m, 2H, NCH_2), 2.97 (hept, J = 6.8 Hz, 2H, CH(CH$_3$)$_2$), 2.53 (s, 3H, CH_3), 1.39 (d, J = 6.8 Hz, 6H, CH(CH_3)$_2$), 1.26 (d, J = 6.9 Hz, 6H, CH(CH_3)$_2$). **^{13}C{^{1}H} NMR** (101 MHz, $CDCl_3$) δ 201.0 ($C_{carbene}$-Au), 157.3 (C_{py}), 151.9 (C_{py}), 146.2 (C_{Ar}, 2C), 138.3 (CH$_{py}$), 134.7 (C_{Ar}), 130.3 (CH$_{Ar}$), 124.8 (CH$_{Ar}$, 2C), 120.5 (CH$_{py}$), 111.4 (CH$_{py}$), 53.1 (NCH$_2$), 48.7 (NCH$_2$), 28.7 (CH(CH$_3$)$_2$, 2C), 25.0 (CH(CH$_3$)$_2$, 2C), 24.6 (CH(CH$_3$)$_2$, 2C), 24.4 (CH$_3$). **HRMS (ESI+)**: calcd for $C_{21}H_{27}AuIN_3$ [M+H]$^+$: *m/z* 646.0988; found: *m/z* 646.0984; [M+Na]$^+$: *m/z* 668.0807; found: 668.0840; [2M-I]$^+$: *m/z* 1163.2780; found: 1163.2804. **IR-FT (ATR)** ṽ (cm^{-1}): 2961, 2923, 2862, 1710, 1676, 1595, 1576, 1493, 1431, 1320, 1274, 1054, 810, 787, 766, 735, 663, 457.

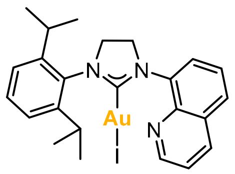

Complex 2. The imidazolinium iodide salt **L2** (180.0 mg, 0.37 mmol, 1.0 eq.), K_2CO_3 (261.2 mg, 1.89 mmol, 5.1 eq.) and [AuCl(SMe_2)] (139.1 mg, 0.47 mmol, 1.3 eq.) were reacted in acetonitrile (2 mL). After the filtration over Celite®, the product was purified by filtering the residue over a pad of silica using DCM. The solvent was removed to obtain a yellow solid. It was washed with hexane and diethyl ether to afford complex **2** as a white solid (105.8 mg, 42% yield). **^{1}H NMR** (400 MHz, $CDCl_3$) δ 8.96 (dd, *J* = 4.2, 1.7 Hz, 1H, CH_{Quin}), 8.23 (dd, *J* = 8.3, 1.7 Hz, 1H, CH_{Quin}), 8.10 (dd, *J* = 7.3, 1.4 Hz, 1H, CH_{Quin}), 7.87 (dd, *J* = 8.2, 1.4 Hz, 1H, CH_{Quin}), 7.60 (dd, *J* = 8.3, 7.4 Hz, 1H, CH_{Quin}), 7.50 (dd, *J* = 8.3, 4.2 Hz, 1H, CH_{Quin}), 7.42 (t, *J* = 7.8 Hz, 1H, CH_{Ar}), 7.25 (d, *J* = 7.8 Hz, 2H, CH_{Ar}), 4.72 – 4.64 (m, 2H, NCH_2), 4.12 – 4.04 (m, 2H, NCH_2), 3.23 (hept, *J* = 6.9 Hz, 2H, $CH(CH_3)_2$), 1.43 (d, *J* = 6.8 Hz, 6H, $CH(CH_3)_2$), 1.39 (d, *J* = 6.8 Hz, 6H, $CH(CH_3)_2$). **^{13}C{^{1}H} NMR** (101 MHz, $CDCl_3$) δ 203.7 ($C_{carbene}$-Au), 150.4 (CH_{Quin}), 146.8 (C_{Ar}, 2C), 144.1 (C_{Quin}), 137.6 (C_{Quin}), 136.6 (CH_{Quin}), 134.6 (C_{Ar}), 130.0 (CH_{Ar}), 129.6 (C_{Quin}), 128.8 (CH_{Quin}), 128.3 (CH_{Quin}), 126.4 (CH_{Quin}), 124.7 (CH_{Ar}, 2C), 122.0 (CH_{Quin}), 54.4 (NCH_2), 53.0 (NCH_2), 28.7 ($CH(CH_3)_2$, 2C), 25.3 ($CH(CH_3)_2$, 2C), 24.6 ($CH(CH_3)_2$, 2C). **HRMS (ESI+)**: calcd for $C_{24}H_{27}AuIN_3$ $[M+H]^+$: *m/z* 682.0988; found: *m/z* 682.0974; $[2M-I]^+$: *m/z* 1235.2780; found: 1235.2746.

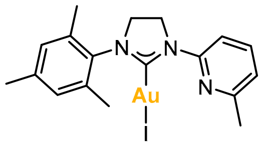

Complex 3. The imidazolinium iodide salt **L3** (67.5 mg, 0.17 mmol, 1.0 eq.), K_2CO_3 (91.1 mg, 0.66 mmol, 4.0 eq.) and [AuCl(SMe_2)] (63.8 mg, 0.22 mmol, 1.3 eq.) were reacted in acetonitrile (1 mL). The product was purified by column chromatography using DCM. By slow diffusion of pentane into a concentrated solution of complex **3** in chloroform, crystals suitable for X-ray diffraction analysis were obtained (32.9 mg, 33% yield). **^{1}H NMR** (400 MHz, $CDCl_3$) δ 8.54 (d, *J* = 8.2 Hz, 1H, CH_{py}), 7.62 (t, *J* = 7.9 Hz, 1H, CH_{py}), 7.02 (d, *J* = 7.5 Hz, 1H, CH_{py}), 6.94 (s, 2H, CH_{Ar}), 4.57 – 4.48 (m, 2H, NCH_2), 3.94 – 3.85 (m, 2H, NCH_2), 2.52 (s, 3H, CH_{3py}), 2.30 (s, 3H, CH_{3Ar}), 2.27 (s, 6H, CH_{3Ar}). **^{13}C{^{1}H} NMR** (101 MHz, $CDCl_3$) δ 192.2 ($C_{carbene}$-Au), 157.3 (C_{py}), 151.9 (C_{py}), 139.3 (C_{Ar}), 138.3 (CH_{py}), 135.5 (C_{Ar}), 135.1 (C_{Ar}, 2C), 130.0 (CH_{Ar}, 2C), 120.6 (CH_{py}), 112.0 (CH_{py}), 50.3 (NCH_2), 48.8 (NCH_2), 24.3 (CH_{3py}), 21.2 (CH_{3Ar}), 18.2 (CH_{3Ar}, 2C). **HRMS (ESI+)**: calcd for $C_{18}H_{21}AuIN_3$ $[M+Na]^+$: *m/z* 626.0338; found: *m/z* 626.0336; $[(C_{18}H_{21}N_3)_2Au]^+$: *m/z* 755.3131; found: 755.3150; $[2M-I]^+$: *m/z* 1079.1841; found: 1079.1830.

S3.2. Synthesis of complex 4

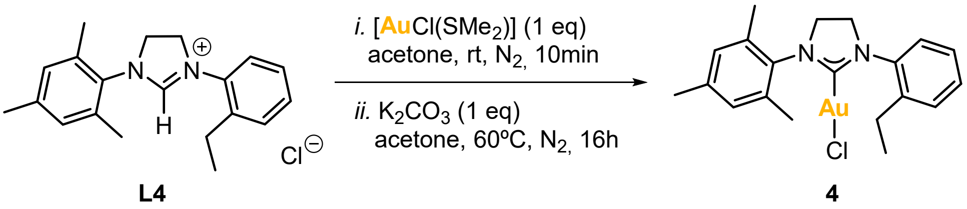

Scheme S3. Synthesis of complex **4**.

For the synthesis of NHC-Au(I) complex **4**, imidazolinium salt **L4** (60.1 mg, 0.18 mmol, 1.0 eq) and chloro(dimethylsulfide)gold(I) (54.8 mg, 0.19 mmol, 1.0 eq) were mixed in acetone (0.6 mL) under nitrogen atmosphere and stirred at room temperature for 10 minutes. Then, potassium carbonate (27.6 mg, 0.20 mmol, 1.1 eq) was added, and the mixture was stirred and heated at 60ºC for 16 hours. After this time, the reaction mixture was cooled down to room temperature; then, the solvent was removed under reduced pressure, and DCM was added. The mixture was filtered over a pad of silica, which was washed with more DCM, and the resulting solution was concentrated to the minimal volume. Next, pentane was added to precipitate the desired product. It was washed with more pentane, and it was dried under vacuum. Complex **4** was obtained as a white solid (75.5 mg, 67% yield). **^{1}H NMR** (400 MHz, $CDCl_3$) δ 7.40 – 7.24 (m, 4H, CH_{Ar}), 6.94 (d, J = 0.7 Hz, 2H, CH_{Ar}), 4.11 (t, J = 9.6 Hz, 2H, NCH_2), 3.96 (td, J = 10.4, 1.7 Hz, 2H, NCH_2), 2.76 (q, J = 7.6 Hz, 2H, C$H_2$$CH_3$), 2.31 (s, 6H, C$H_3$), 2.30 (s, 3H, C$H_3$), 1.35 (t, J = 7.6 Hz, 3H, CH_2CH_3). **^{13}C{^{1}H} NMR** (101 MHz, $CDCl_3$) δ 195.0 ($C_{carbene}$-Au), 141.2 (C_{Ar}), 139.1 (C_{Ar}), 138.4 (C_{Ar}), 135.6 (C_{Ar}, 2C), 134.8 (C_{Ar}), 129.9 (CH_{Ar}, 2C), 129.9 (CH_{Ar}), 129.5 (CH_{Ar}), 128.4 (CH_{Ar}), 127.5 (CH_{Ar}), 53.3 (NCH_2), 51.0 (NCH_2), 24.4 (CH_2CH_3), 21.2 (CH_3), 18.1 (CH_3, 2C), 15.0 (CH_2CH_3). **HRMS (ESI+)**: calcd for $C_{20}H_{24}AuClN_2$ [M+Na]$^+$: *m/z* 547.1186; found: *m/z* 547.1181; [2M+Na]$^+$: *m/z* 1071.2479; found: 1071.2438; [2M-Cl]$^+$: *m/z* 1013.2899; found: 1013.2865. **IR-FT (ATR)** ṽ (cm^{-1}): 2951, 2919, 2863, 1500, 1453, 1437, 1321, 1276, 1194, 1120, 1034, 1018, 852, 844, 769, 739, 658, 606, 580, 572, 549, 517, 442.

S4. Reactivity and products

The gold(I) complexes were reacted with two equivalents of an oxidant and, in some cases, one equivalent of a silver salt was used as additive. The reactivity towards the formation of Au(0) nuggets and azolones is displayed in Table S1. All reactions were run in a sealed vial under nitrogen atmosphere, and were heated and stirred overnight in the absence of light.

Table S1. Reactivity of gold(I) complexes towards the formation of Au(0) nuggets and azolones.

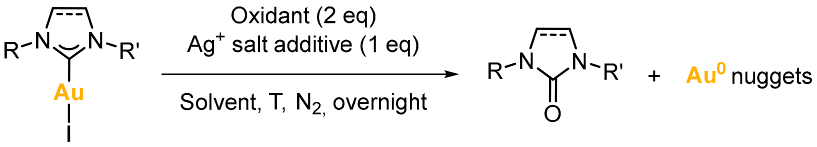

Entry	Complex	Oxidant	Additive	Solvent	T (°C)	Yield to Au(0)	NHC=O (yield %)*
1	**1**	$PhI(OAc)_2$	AgOAc	1,2-DCE	90	75	Detected MS
2	**1**	$PhI(OAc)_2$	-	1,2-DCE	90	46	Detected MS
3	**1**	$PhI(OAc)_2$	-	MeCN	90	60	**L1^{ox}-I** (53)
4	**1**	H_2O_2	AgOAc	1,2-DCE	90	0	0
5	**1**	H_2O_2	-	1,2-DCE	90	0	Detected MS
6	**1**	$PhICl_2$	-	DCM	rt	0	Detected MS
7	**2**	-	-	1,2-DCE	100	0	0
8	**2**	$PhI(OAc)_2$	AgOAc	1,2-DCE	90	85	Detected MS
9	**2**	$PhI(OAc)_2$	-	1,2-DCE	90	90	**L2^{ox}** (22)<br>**L2^{ox}-I** (17)
10	**2**	$PhI(OAc)_2$	AgOAc	DCM	70	91	Detected MS
11	**2**	$PhI(OAc)_2$	-	MeCN	90	56	**L2^{ox}** (34)
12	**2**	CH_3CO_3H	-	1,2-DCE	90	34	Detected NMR
13	**3**	$PhI(OAc)_2$	AgOAc	1,2-DCE	90	>99	**L3^{ox}** (60)
14	**3**	XeF_2	-	$CDCl_3$	rt	0	Detected MS
15	**4**	$PhI(OAc)_2$	-	DCM	rt	0	0
16	**4**[a]	$PhI(OAc)_2$	-	DCM	100	97	Detected MS
17	**4**	$PhI(OAc)_2$	-	1,2-DCE	90	32	Detected MS
18	(IPr)AuCl	$PhI(OAc)_2$	AgOAc	1,2-DCE	90	0	0
19	(IPr)AuCl	$PhI(OAc)_2$	-	1,2-DCE	90	0	0
20	(SIPr)AuCl	$PhI(OAc)_2$	AgOAc	1,2-DCE	90	0	0
21	(SIPr)AuCl	$PhI(OAc)_2$	-	1,2-DCE	90	11	6

*Isolated yield. [a]5h of reaction.

The azolones that were isolated from the reaction crudes were characterized by NMR and HRMS as described below.

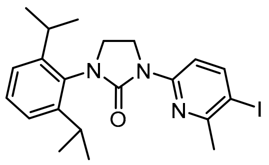

Compound L1^{ox}-I. ^{1}H NMR (400 MHz, 228K, $CDCl_3$) δ 7.91 – 7.83 (m, 2H, CH_{py}), 7.38 (t, J = 7.7 Hz, 1H, CH_{Ar}), 7.23 (d, J = 7.6 Hz, 2H, CH_{Ar}), 4.23 (t, J = 8.2 Hz, 2H, NCH_2), 3.71 (t, J = 8.2 Hz, 2H, NCH_2), 2.98 (hept, J = 6.8 Hz, 2H, $CH(CH_3)_2$), 2.63 (s, 3H, CH_3), 1.23 (d, J = 6.9 Hz, 6H, $CH(CH_3)_2$), 1.20 (d, J = 6.6 Hz, 6H, $CH(CH_3)_2$). **^{13}C{^{1}H} NMR** (101 MHz, $CDCl_3$) δ 157.7 (C_{py}), 156.2 (C=O), 152.1 (C_{py}), 148.0 (C_{Ar}, 2C), 147.3 (CH_{py}), 132.8 (C_{Ar}), 129.2 (CH_{Ar}), 124.3 (CH_{Ar}, 2C), 112.2

(*C*H$_{py}$), 86.2 (*C*$_{py}$-I), 45.7 (N*C*H$_2$), 41.7 (N*C*H$_2$), 28.9 (*C*H(CH$_3$)$_2$, 2C), 28.8 (*C*H$_3$), 24.6 (CH(*C*H$_3$)$_2$, 2C), 24.4 (CH(*C*H$_3$)$_2$, 2C). **HRMS (ESI+)**: calcd for $C_{21}H_{26}IN_3O$ $[M+H]^+$: *m/z* 464.1193; found: *m/z* 464.1193; $[M+Na]^+$: *m/z* 486.1013; found: *m/z* 486.1012; $[2M+Na]^+$: *m/z* 949.2133; found: *m/z* 949.2141.

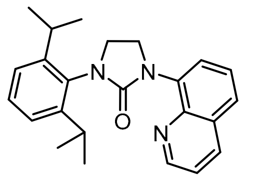

Compound L2^{ox}. ^{1}H NMR (400 MHz, $CDCl_3$) δ 8.93 (dd, *J* = 4.2, 1.8 Hz, 1H, C*H*$_{Ar}$), 8.17 (dd, *J* = 8.3, 1.8 Hz, 1H, C*H*$_{Ar}$), 7.93 (dd, *J* = 7.5, 1.4 Hz, 1H, C*H*$_{Ar}$), 7.71 (dd, *J* = 8.2, 1.4 Hz, 1H, C*H*$_{Ar}$), 7.56 (dd, *J* = 8.2, 7.5 Hz, 1H, C*H*$_{Ar}$), 7.42 (dd, *J* = 8.3, 4.1 Hz, 1H, C*H*$_{Ar}$), 7.35 (dd, *J* = 8.3, 7.1 Hz, 1H, C*H*$_{Ar}$), 7.23 (d, *J* = 7.7 Hz, 2H, C*H*$_{Ar}$), 4.45 – 4.40 (m, 2H, NC*H*$_2$), 3.90 – 3.84 (m, 2H, NC*H*$_2$), 3.32 (hept, *J* = 6.9 Hz, 2H, C*H*(CH$_3$)$_2$), 1.32 (d, *J* = 6.9 Hz, 6H, CH(C*H*$_3$)$_2$), 1.30 (d, *J* = 6.8 Hz, 6H, CH(C*H*$_3$)$_2$). **^{13}C{^{1}H} NMR** (101 MHz, $CDCl_3$) δ 159.5 (C=O), 149.3 (*C*H$_{Quin}$), 148.5 (*C*$_{Ar}$, 2C), 144.3 (*C*$_{Quin}$), 138.3 (*C*$_{Quin}$), 136.5 (*C*H$_{Quin}$), 133.8 (*C*$_{Ar}$), 129.7 (*C*$_{Quin}$), 128.8 (*C*H$_{Ar}$), 127.5 (*C*H$_{Quin}$), 126.7 (*C*H$_{Quin}$), 126.1 (*C*H$_{Quin}$), 124.2 (*C*H$_{Ar}$, 2C), 121.3 (*C*H$_{Quin}$), 47.4 (N*C*H$_2$), 47.0 (N*C*H$_2$), 28.8 (*C*H(CH$_3$)$_2$, 2C), 24.8 (CH(*C*H$_3$)$_2$, 2C), 24.5 (CH(*C*H$_3$)$_2$, 2C). **HRMS (ESI+)**: calcd for $C_{24}H_{27}N_3O$ $[M+H]^+$: *m/z* 374.2227; found: *m/z* 374.2211; $[M+Na]^+$: *m/z* 396.2046; found: 396.2036; $[2M+Na]^+$: *m/z* 769.4200; found: *m/z* 769.4164.

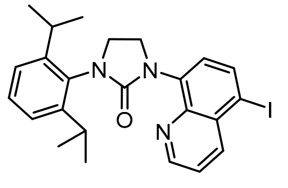

Compound L2^{ox}-I. ^{1}H NMR (400 MHz, $CDCl_3$) δ 8.88 (dd, *J* = 4.1, 1.6 Hz, 1H, C*H*$_{Quin}$), 8.39 (dd, *J* = 8.5, 1.6 Hz, 1H, C*H*$_{Quin}$), 8.13 (d, *J* = 8.1 Hz, 1H, C*H*$_{Quin}$), 7.69 (d, *J* = 8.0 Hz, 1H, C*H*$_{Quin}$), 7.49 (dd, *J* = 8.6, 4.1 Hz, 1H, C*H*$_{Quin}$), 7.35 (dd, *J* = 8.3, 7.1 Hz, 1H, C*H*$_{Ar}$), 7.23 (d, *J* = 7.4 Hz, 2H, C*H*$_{Ar}$), 4.46 – 4.39 (m, 2H, NC*H*$_2$), 3.89 – 3.83 (m, 2H, NC*H*$_2$), 3.28 (hept, *J* = 6.9 Hz, 2H, C*H*(CH$_3$)$_2$), 1.32 (d, *J* = 6.9 Hz, 6H, CH(C*H*$_3$)$_2$), 1.29 (d, *J* = 6.8 Hz, 6H, CH(C*H*$_3$)$_2$). **^{13}C{^{1}H} NMR** (101 MHz, $CDCl_3$) δ 159.2 (C=O), 149.9 (*C*H$_{Quin}$), 148.4 (*C*$_{Ar}$, 2C), 144.8 (*C*$_{Quin}$), 140.9 (*C*H$_{Quin}$), 139.4 (*C*$_{Quin}$), 137.7 (*C*H$_{Quin}$), 133.5 (*C*$_{Ar}$), 131.2 (*C*$_{Quin}$), 129.0 (*C*H$_{Ar}$), 128.5 (*C*H$_{Quin}$), 124.2 (*C*H$_{Ar}$, 2C), 122.9 (*C*H$_{Quin}$), 95.3 (*C*$_{Quin}$-I), 47.4 (N*C*H$_2$), 47.0 (N*C*H$_2$), 28.8 (*C*H(CH$_3$)$_2$, 2C), 24.8 (CH(*C*H$_3$)$_2$, 2C), 24.5 (CH(*C*H$_3$)$_2$, 2C). **HRMS (ESI+)**: calcd for $C_{24}H_{26}IN_3O$ $[M+H]^+$: *m/z* 500.1193; found: *m/z* 500.1184; $[M+Na]^+$: *m/z* 522.1013; found: 522.0999.

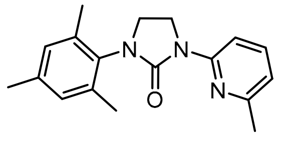

Compound L3^{ox}. ^{1}H NMR (400 MHz, $CDCl_3$) δ 8.11 (d, *J* = 8.4 Hz, 1H, C*H*$_{py}$), 7.51 (t, *J* = 7.9 Hz, 1H, C*H*$_{py}$), 6.93 (s, 2H, C*H*$_{Ar}$), 6.78 (d, *J* = 7.3 Hz, 1H, C*H*$_{py}$), 4.31 – 4.23 (m, 2H, NC*H*$_2$), 3.74 – 3.67 (m, 2H, NC*H*$_2$), 2.47 (s, 3H, C*H*$_{3py}$), 2.29 (s, 3H, C*H*$_{3Ar}$), 2.25 (s, 6H, C*H*$_{3Ar}$). **^{13}C{^{1}H} NMR** (101 MHz, $CDCl_3$) δ 156.3 (*C*$_{py}$), 155.9 (C=O), 152.4 (*C*$_{py}$), 138.0 (*C*$_{Ar}$), 137.7 (*C*H$_{py}$), 137.0 (*C*$_{Ar}$, 2C), 133.1 (*C*$_{Ar}$), 129.5 (*C*H$_{Ar}$, 2C), 116.8 (*C*H$_{py}$), 109.9 (*C*H$_{py}$), 43.2 (N*C*H$_2$), 42.0 (N*C*H$_2$), 24.5 (*C*H$_{3py}$), 21.1 (*C*H$_{3Ar}$), 18.0 (*C*H$_{3Ar}$, 2C). **HRMS (ESI+)**: calcd for $C_{18}H_{21}N_3O$ $[M+H]^+$: *m/z* 296.1757; found: *m/z* 296.1774; $[M+Na]^+$: *m/z* 318.1577; found: 318.1595; $[2M+Na]^+$: *m/z* 613.3261; found: *m/z* 613.3278.

S5. NMR, HRMS-ESI and IR spectra

S5.1. Compound L1

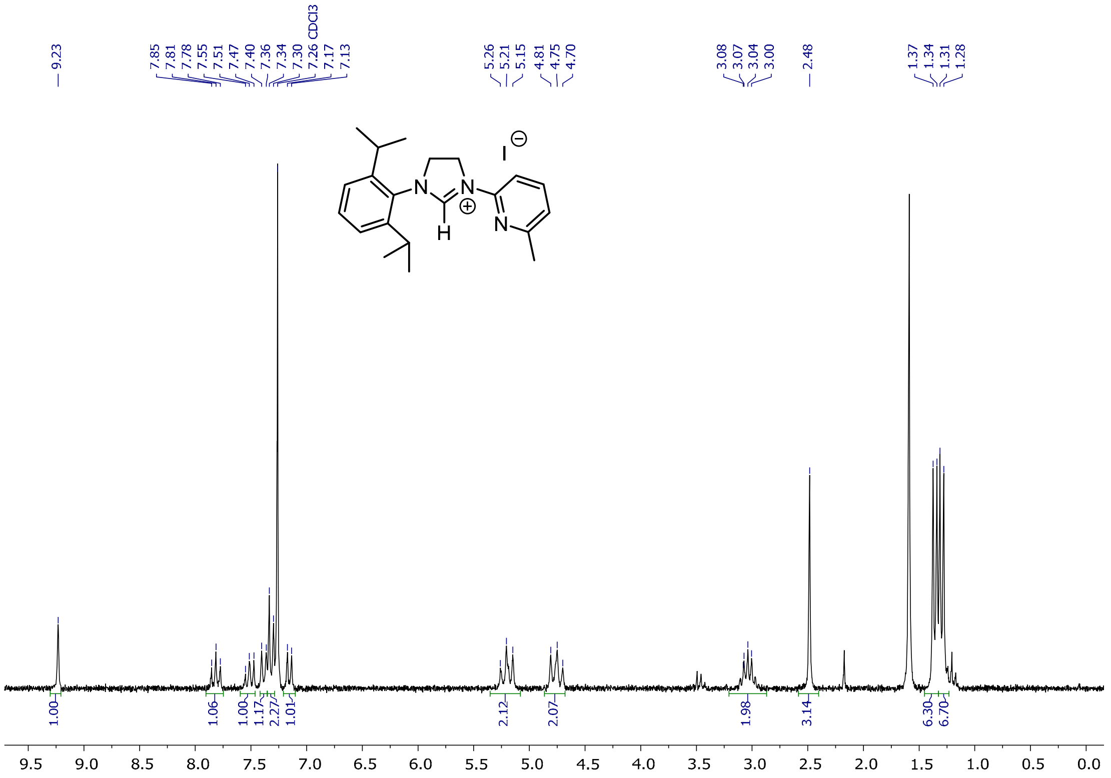

Figure S1. ^{1}H NMR (200MHz, 298K) of **L1** in $CDCl_3$.

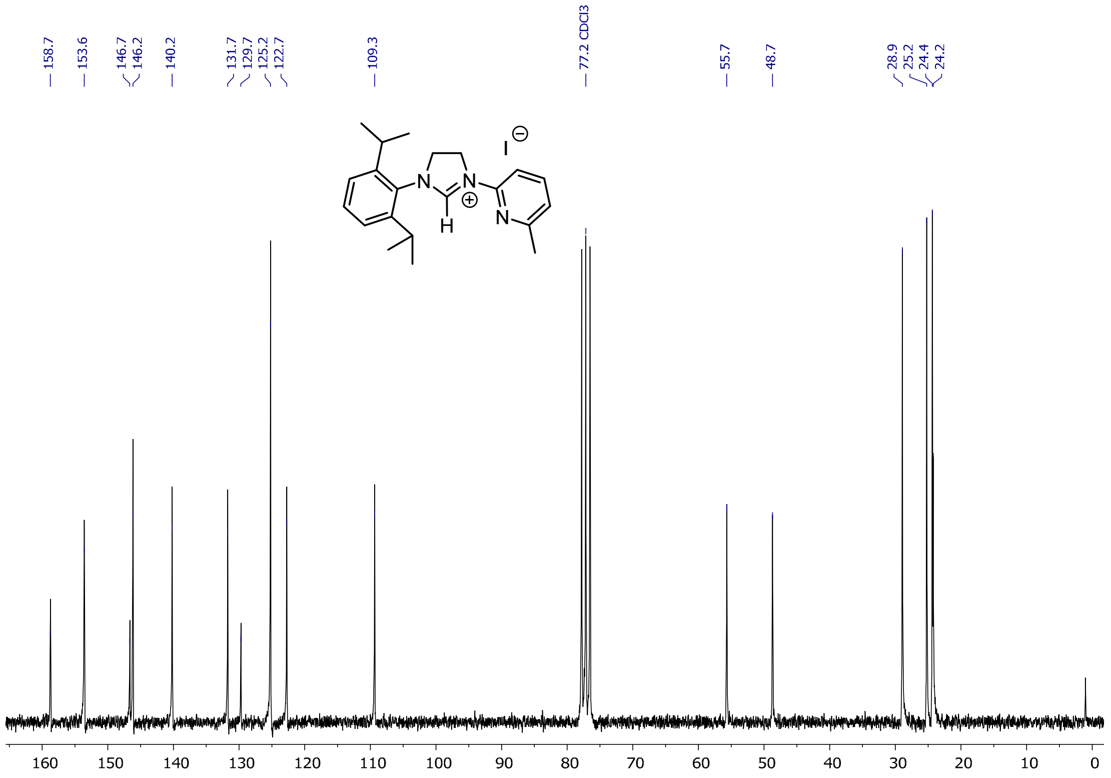

Figure S2. $^{13}C\{^{1}H\}$ NMR (50MHz, 298K) of **L1** in $CDCl_3$.

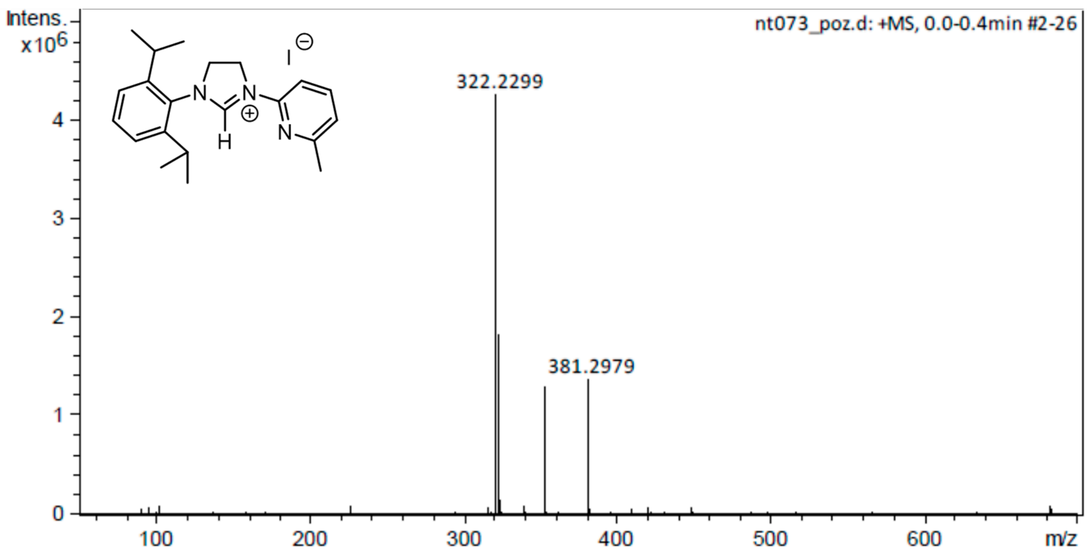

Figure S3. HRMS-ESI(+) of **L1**.

S5.2. Compound L3

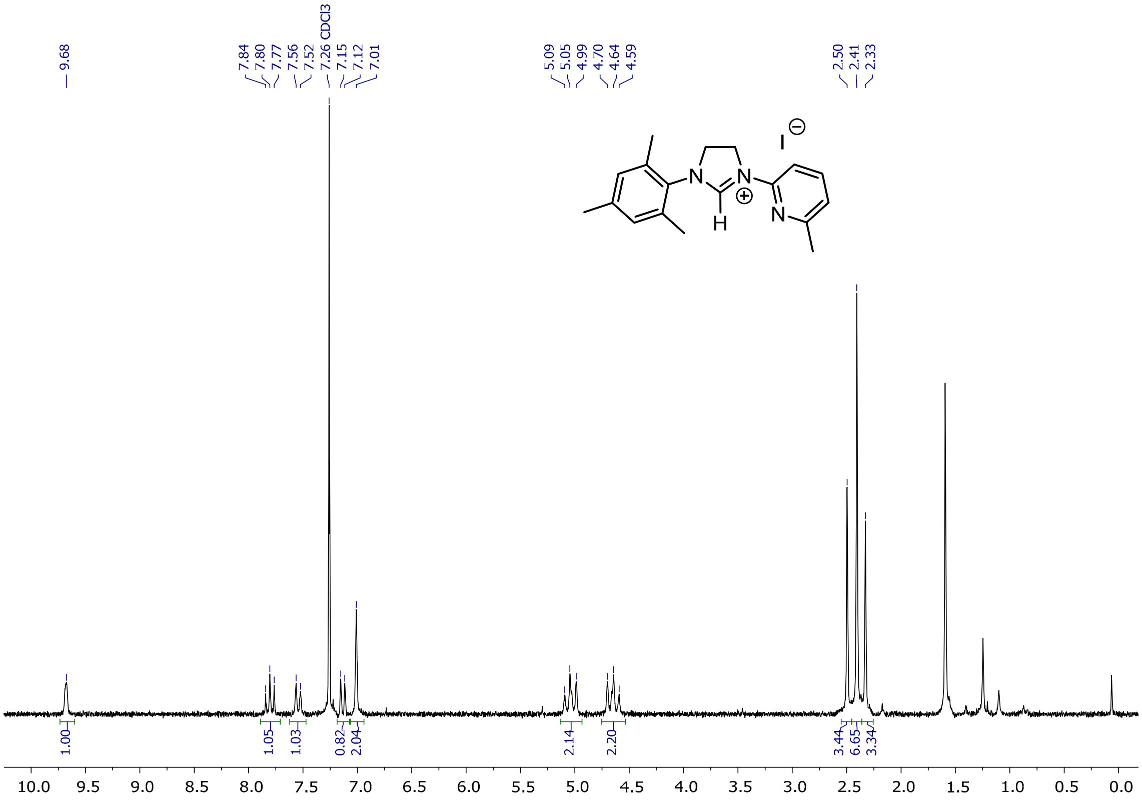

Figure S4. ^{1}H NMR (200MHz, 298K) of **L3** in $CDCl_3$.

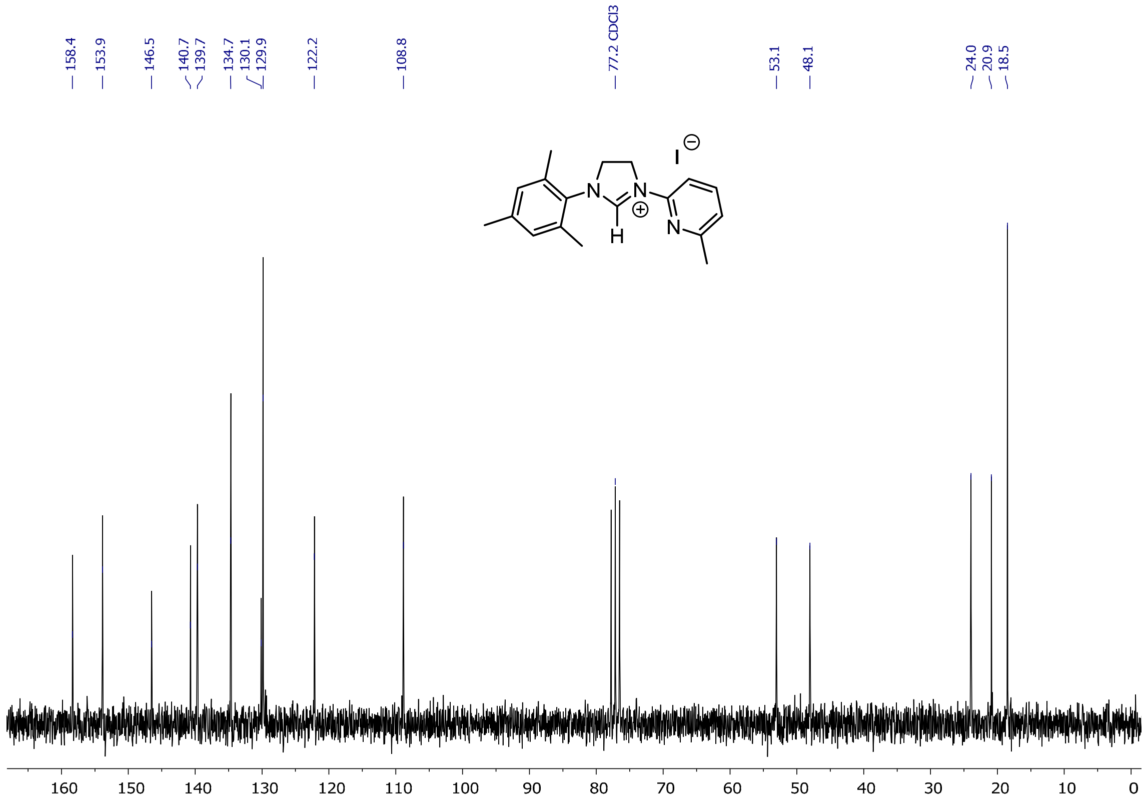

Figure S5. $^{13}C\{^{1}H\}$ NMR (50MHz, 298K) of **L3** in $CDCl_3$.

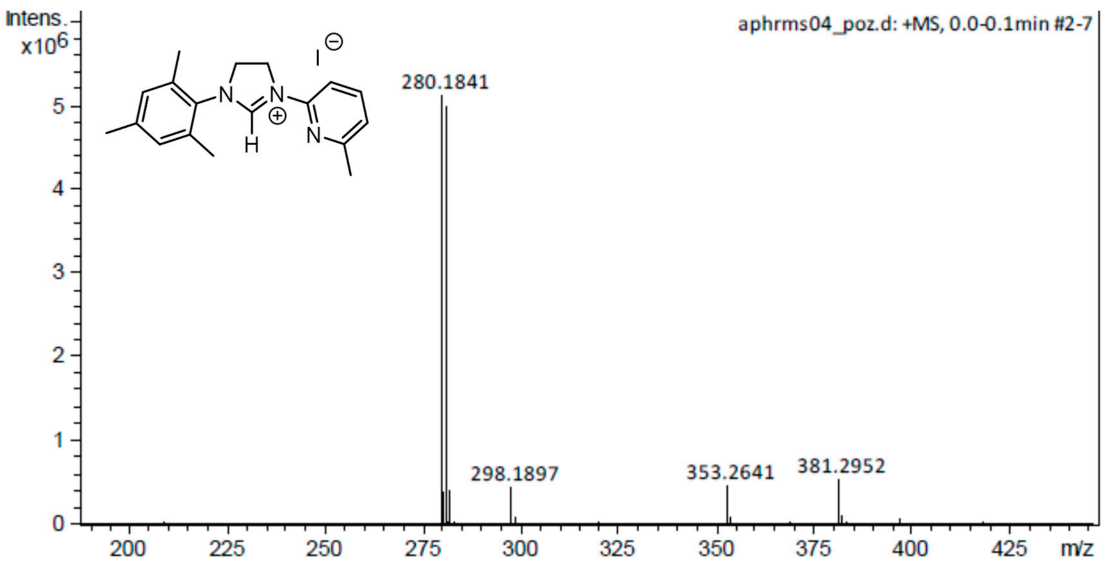

Figure S6. HRMS-ESI(+) of **L3**.

S5.3. Complex 1

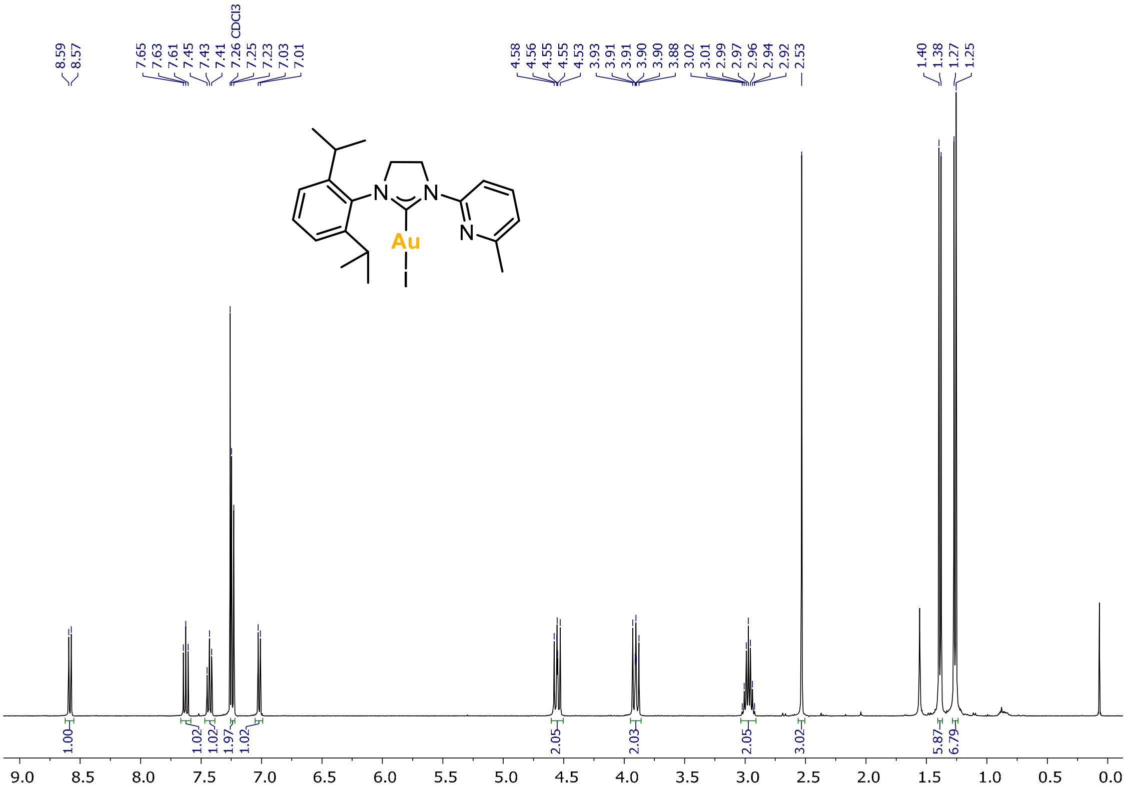

Figure S7. ^{1}H NMR (400MHz, 298K) of **1** in $CDCl_3$.

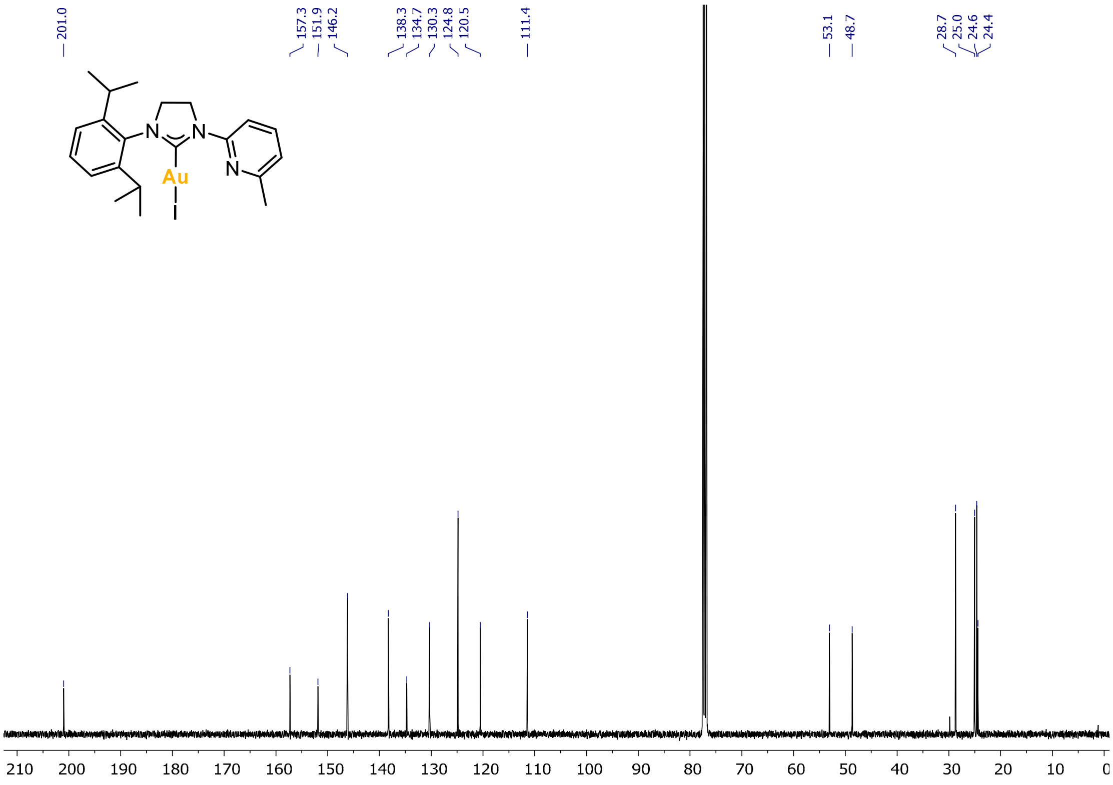

Figure S8. $^{13}C\{^{1}H\}$ NMR (101MHz, 298K) of **1** in $CDCl_3$.

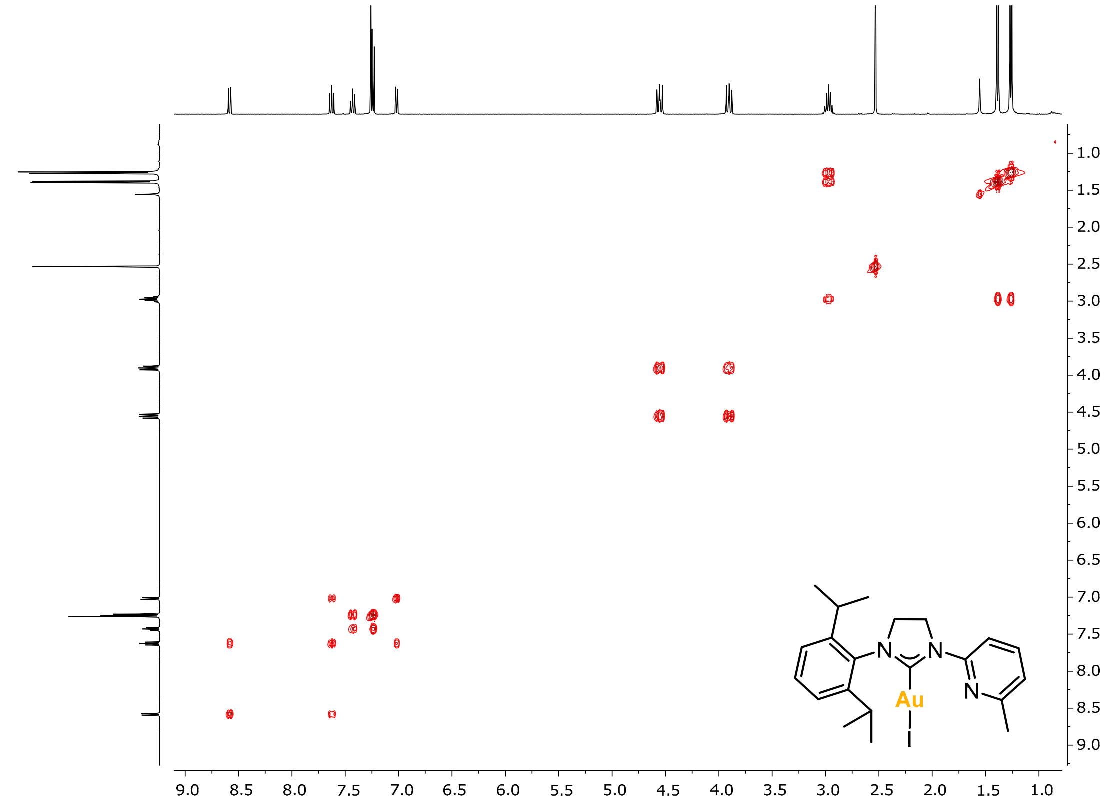

Figure S9. $^{1}H,^{1}H$-COSY NMR (400MHz, 298K) of **1** in $CDCl_3$.

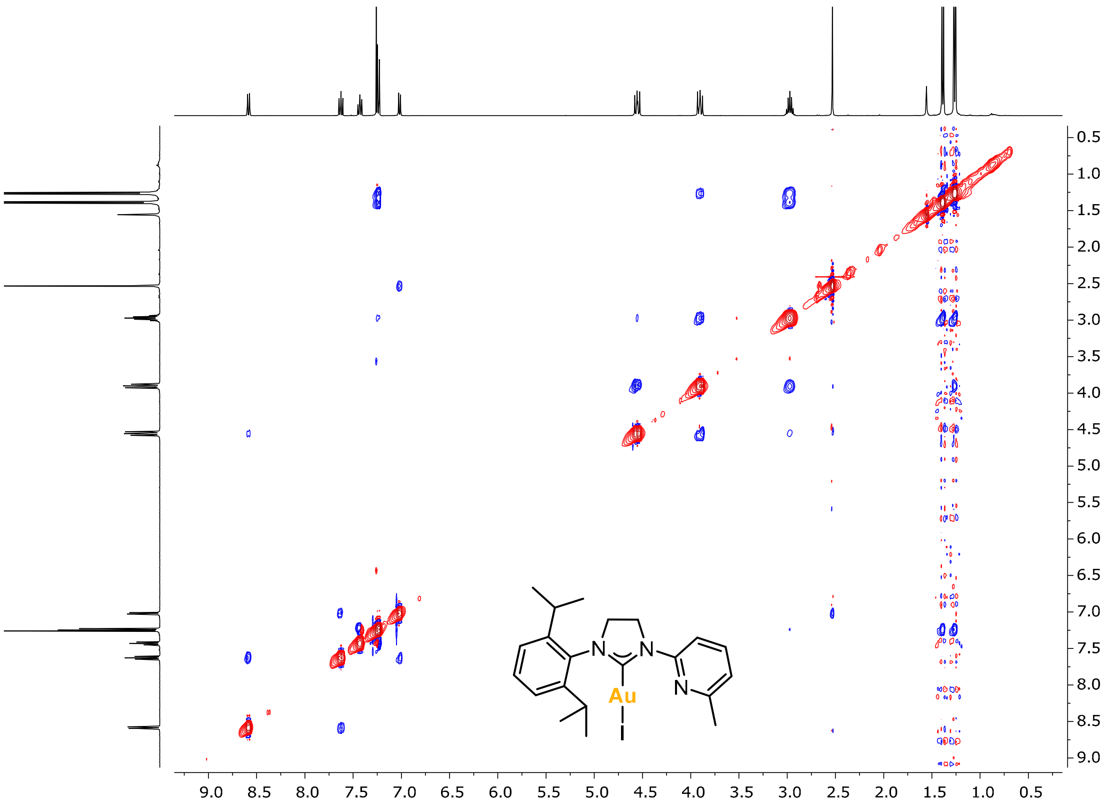

Figure S10. $^{1}H,^{1}H$-NOESY NMR (400MHz, 298K) of **1** in $CDCl_3$.

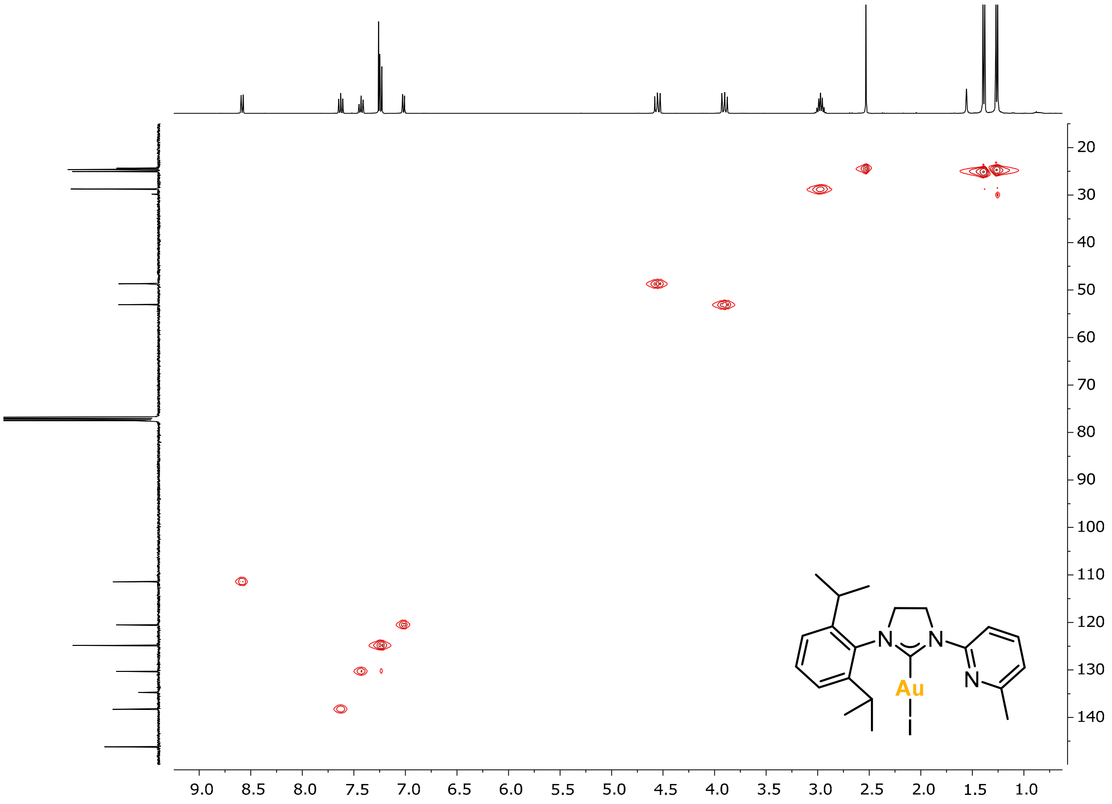

Figure S11. $^{1}H,^{13}C$-HSQC NMR (400MHz, 298K) of **1** in $CDCl_3$.

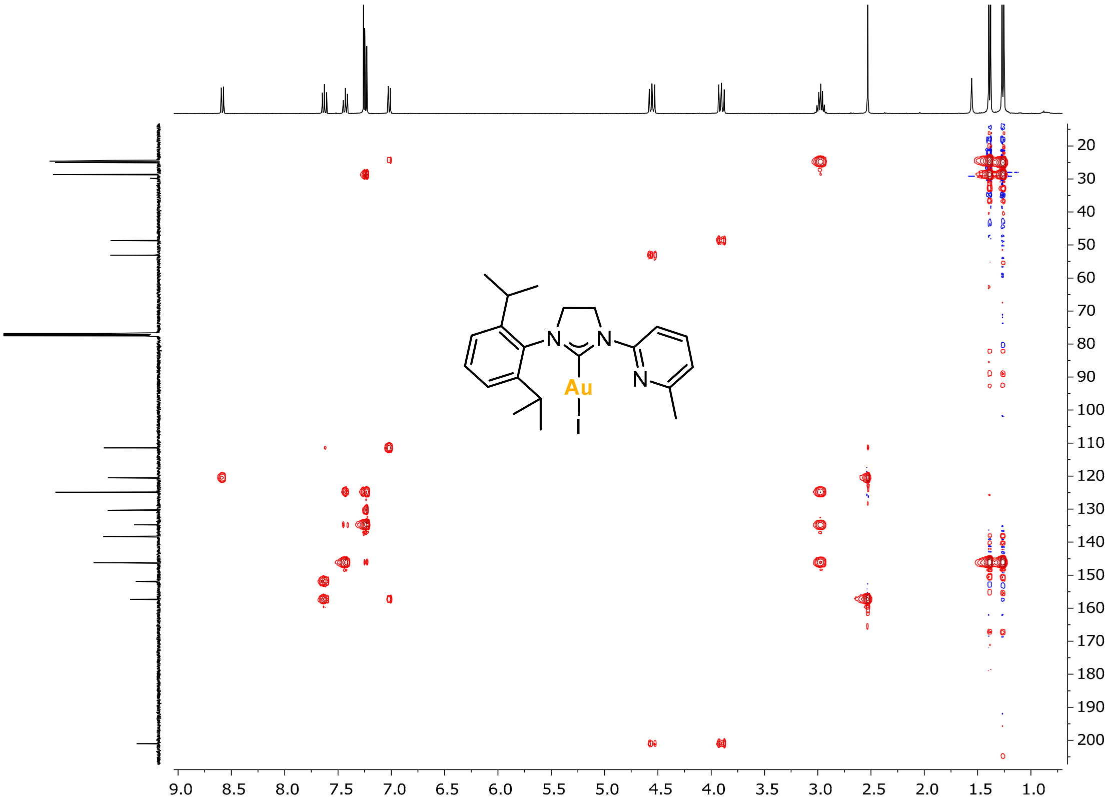

Figure S12. $^{1}H,^{13}C$-HMBC NMR (400MHz, 298K) of **1** in $CDCl_3$.

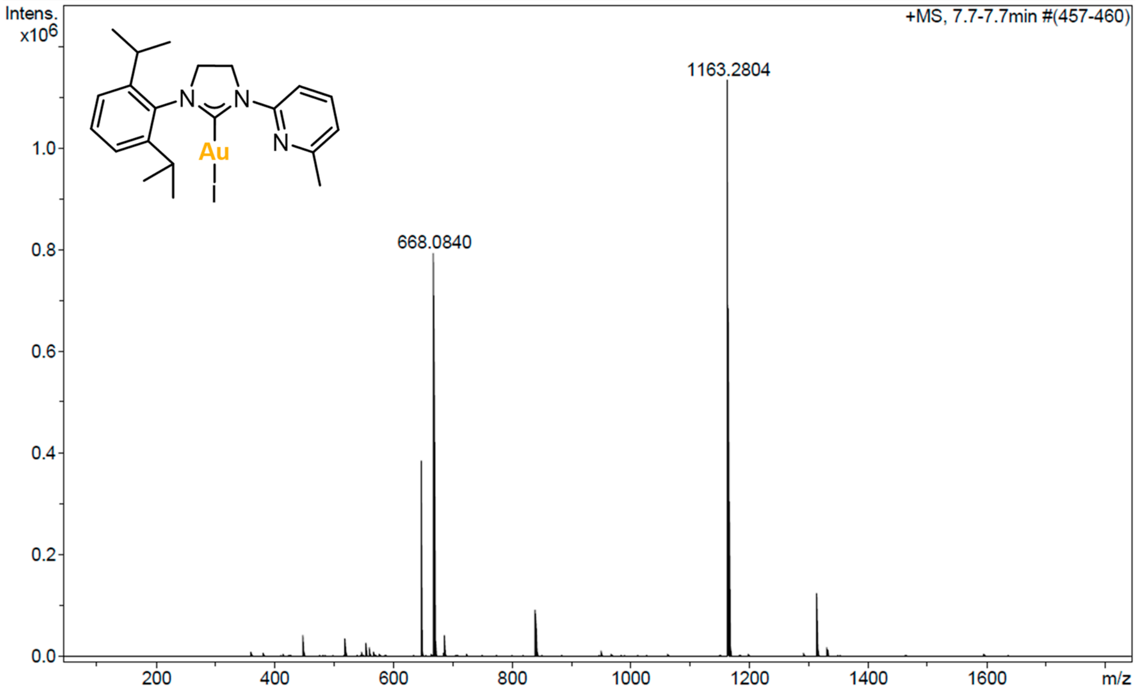

Figure S13. HRMS-ESI(+) of **1**.

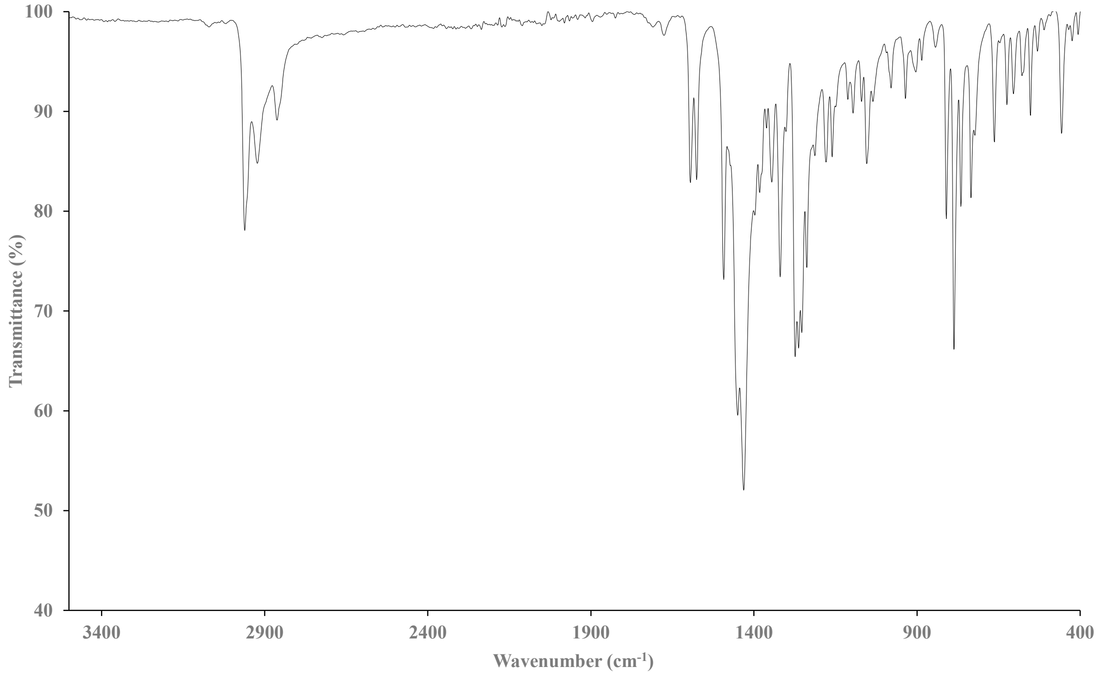

Figure S14. IR spectrum of **1**.

S5.4. Complex 2

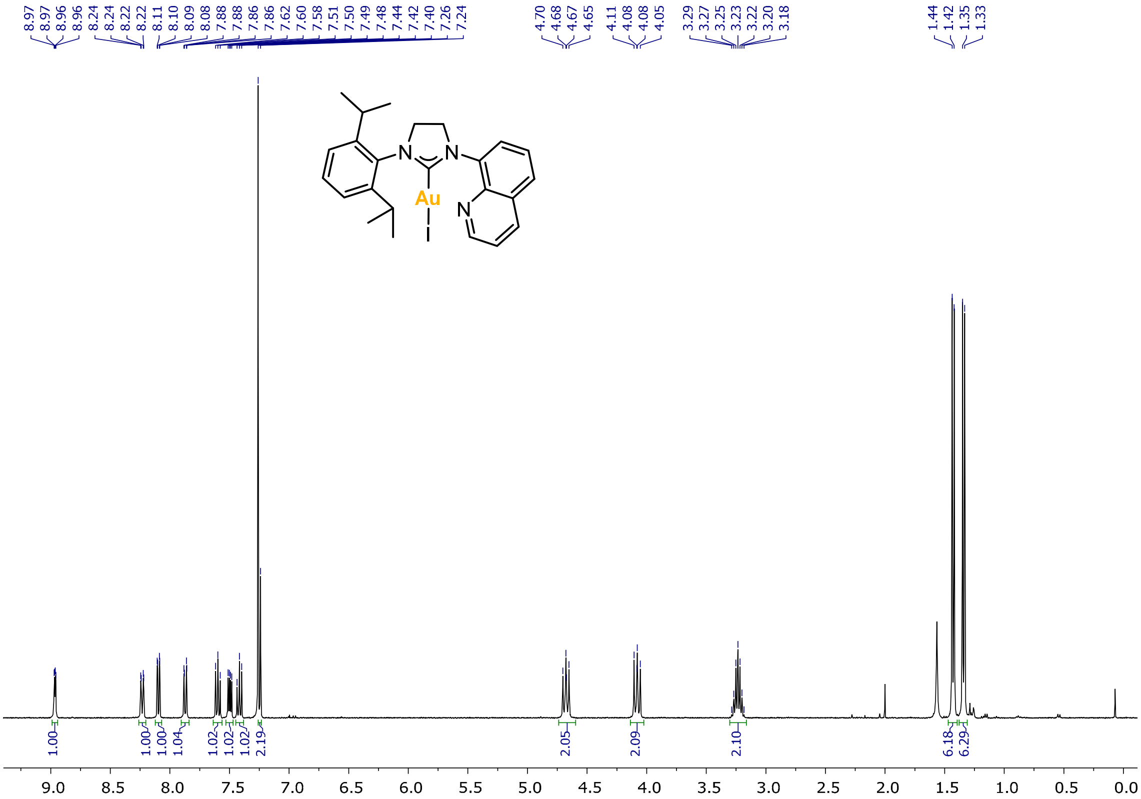

Figure S15. ^{1}H NMR (400MHz, 298K) of **2** in $CDCl_3$.

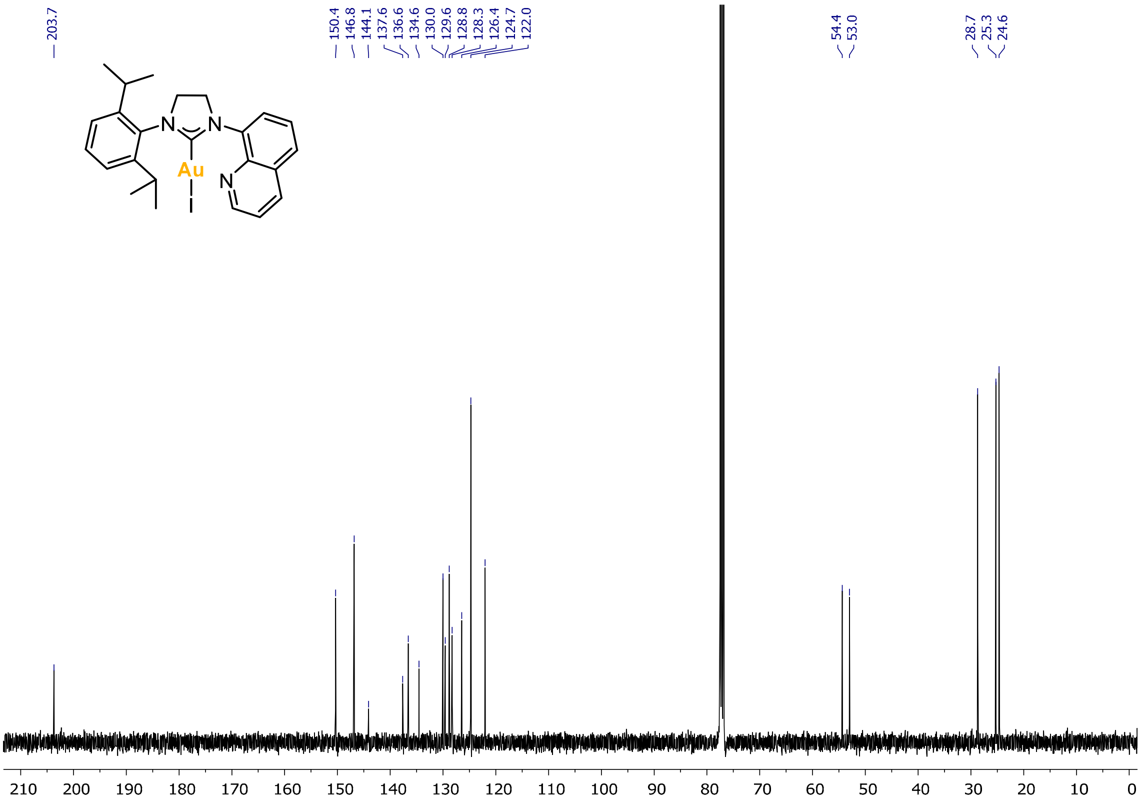

Figure S16. $^{13}C\{^{1}H\}$ NMR (101MHz, 298K) of **2** in $CDCl_3$.

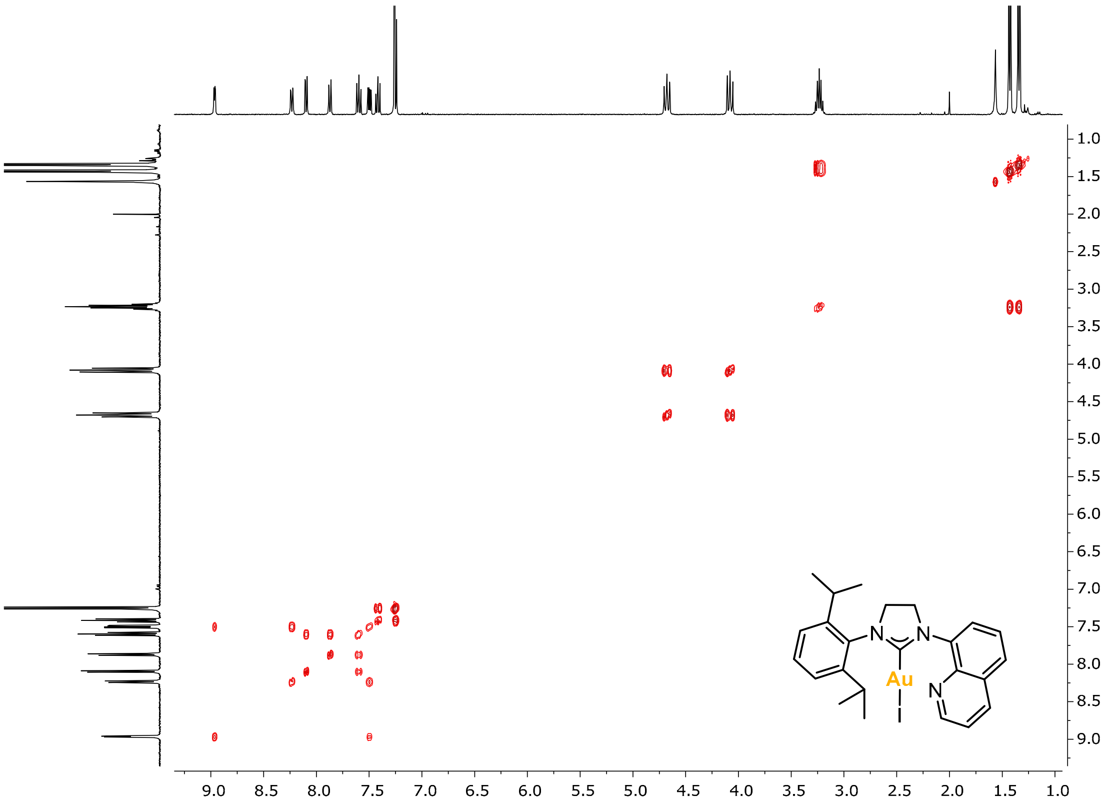

Figure S17. ^{1}H,^{1}H-COSY NMR (400MHz, 298K) of **2** in $CDCl_3$.

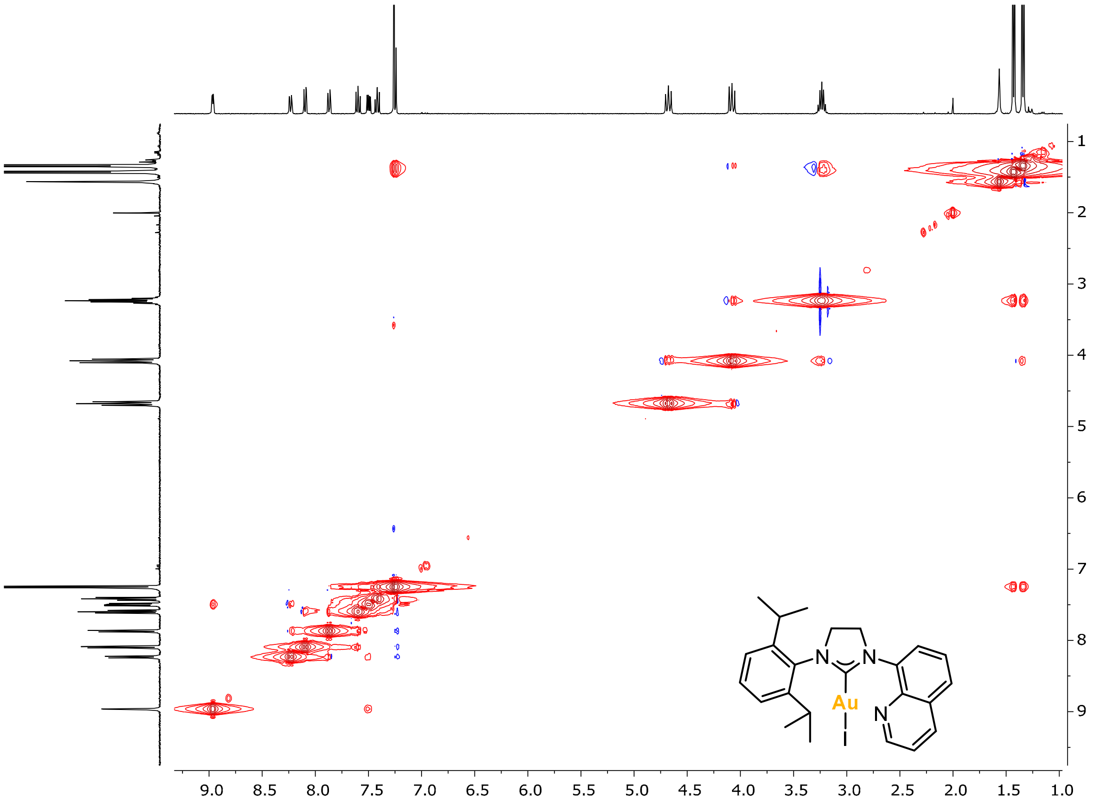

Figure S18. ^{1}H,^{1}H-NOESY NMR (400MHz, 298K) of **2** in $CDCl_3$.

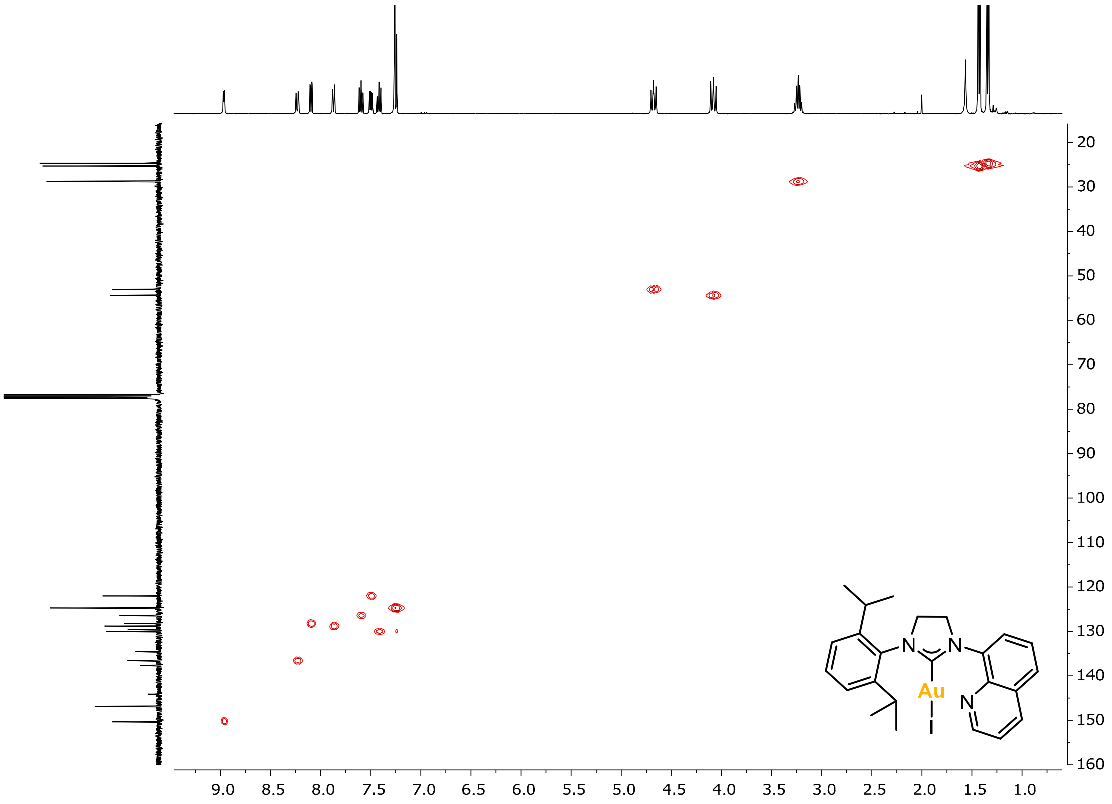

Figure S19. $^{1}H,^{13}C$-HSQC NMR (400MHz, 298K) of **2** in $CDCl_3$.

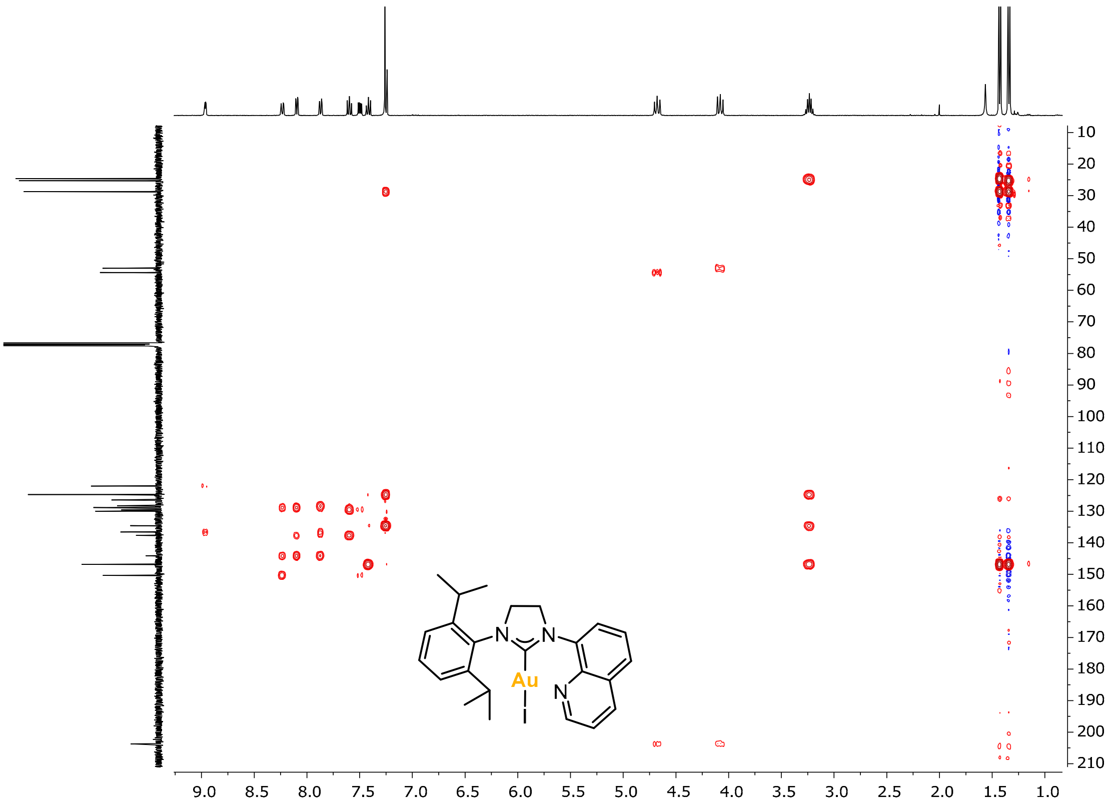

Figure S20. $^{1}H,^{13}C$-HMBC NMR (400MHz, 298K) of **2** in $CDCl_3$.

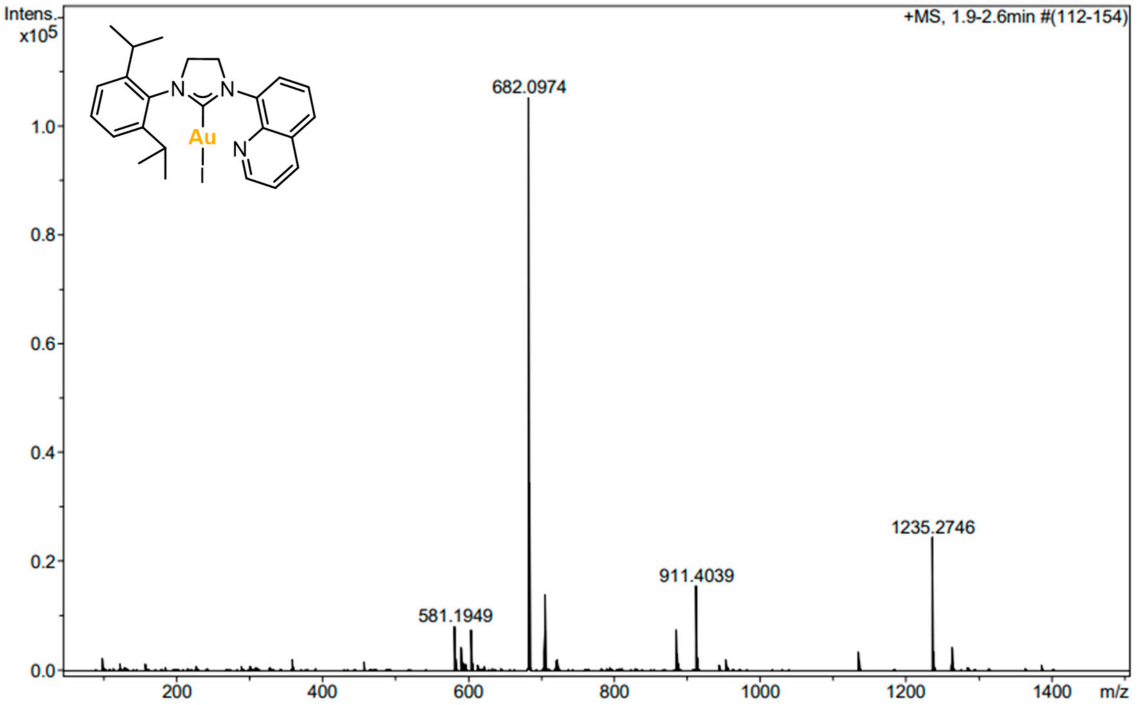

Figure S21. HRMS-ESI(+) of **2**.

S5.5. Complex 3

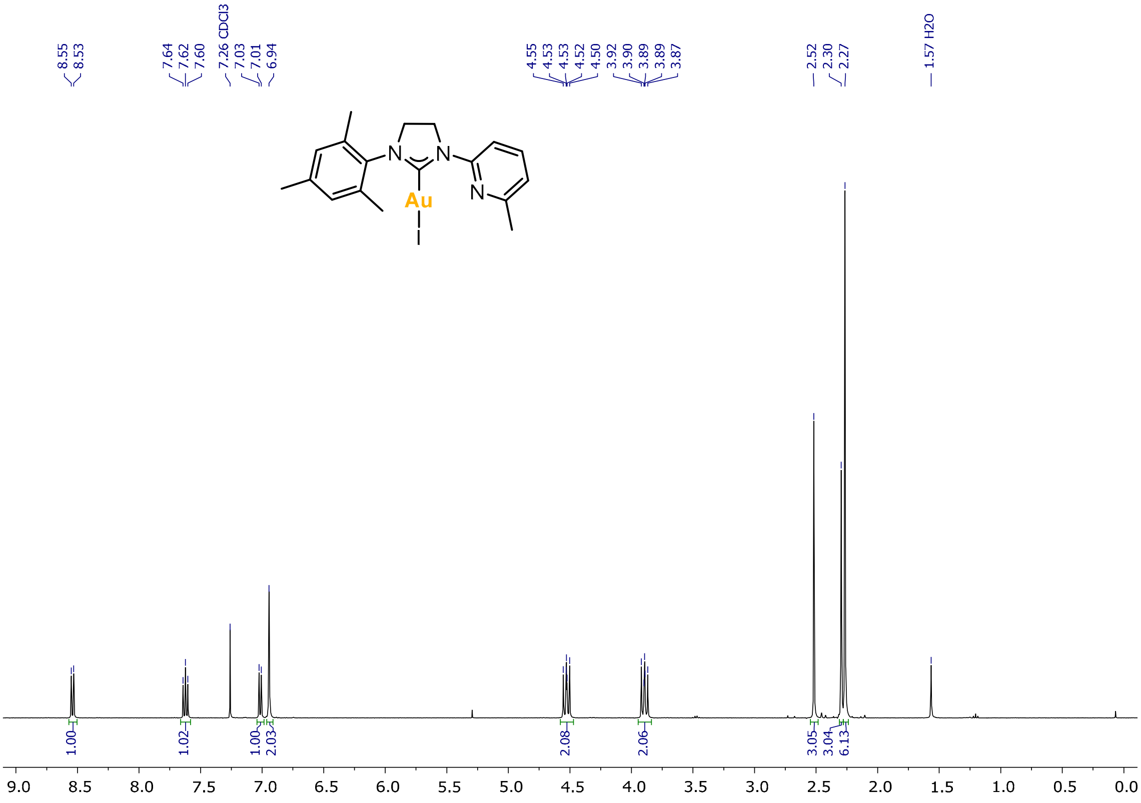

Figure S22. ^{1}H NMR (400MHz, 298K) of **3** in $CDCl_3$.

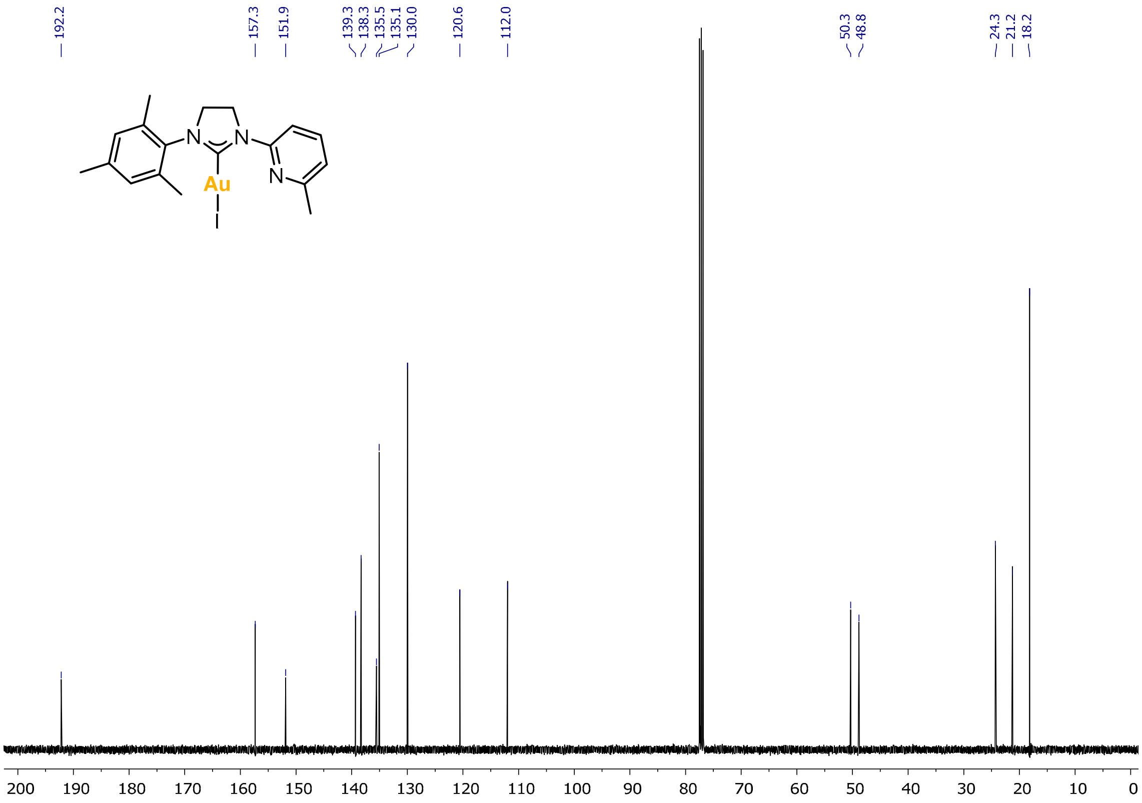

Figure S23. $^{13}C\{^{1}H\}$ NMR (101MHz, 298K) of **3** in $CDCl_3$.

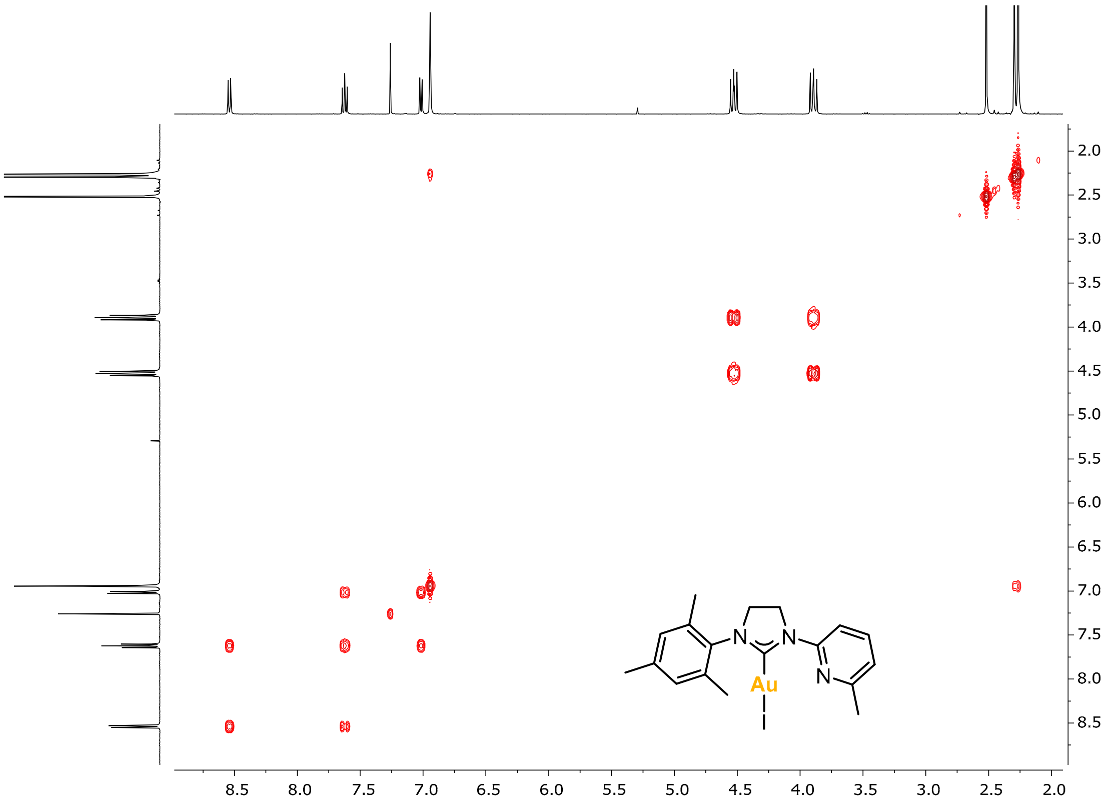

Figure S24. $^{1}H,^{1}H$-COSY NMR (400MHz, 298K) of **3** in $CDCl_3$.

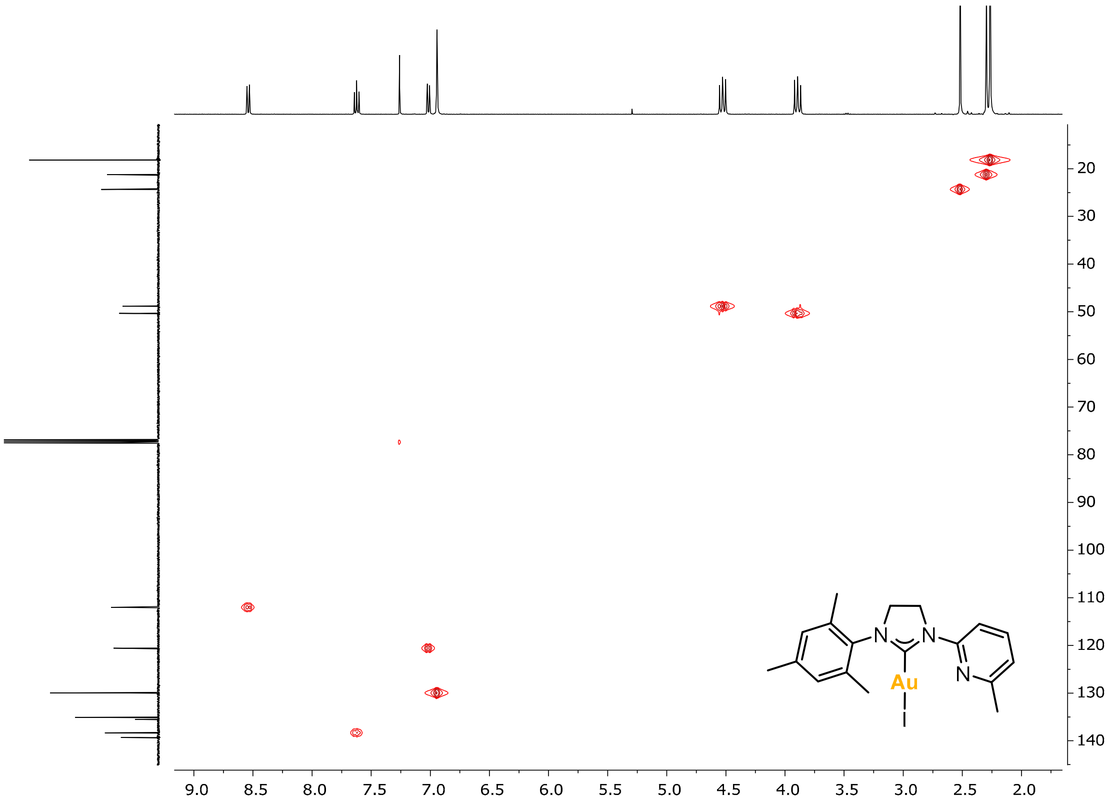

Figure S25. $^{1}H,^{13}C$-HSQC NMR (400MHz, 298K) of **3** in $CDCl_3$.

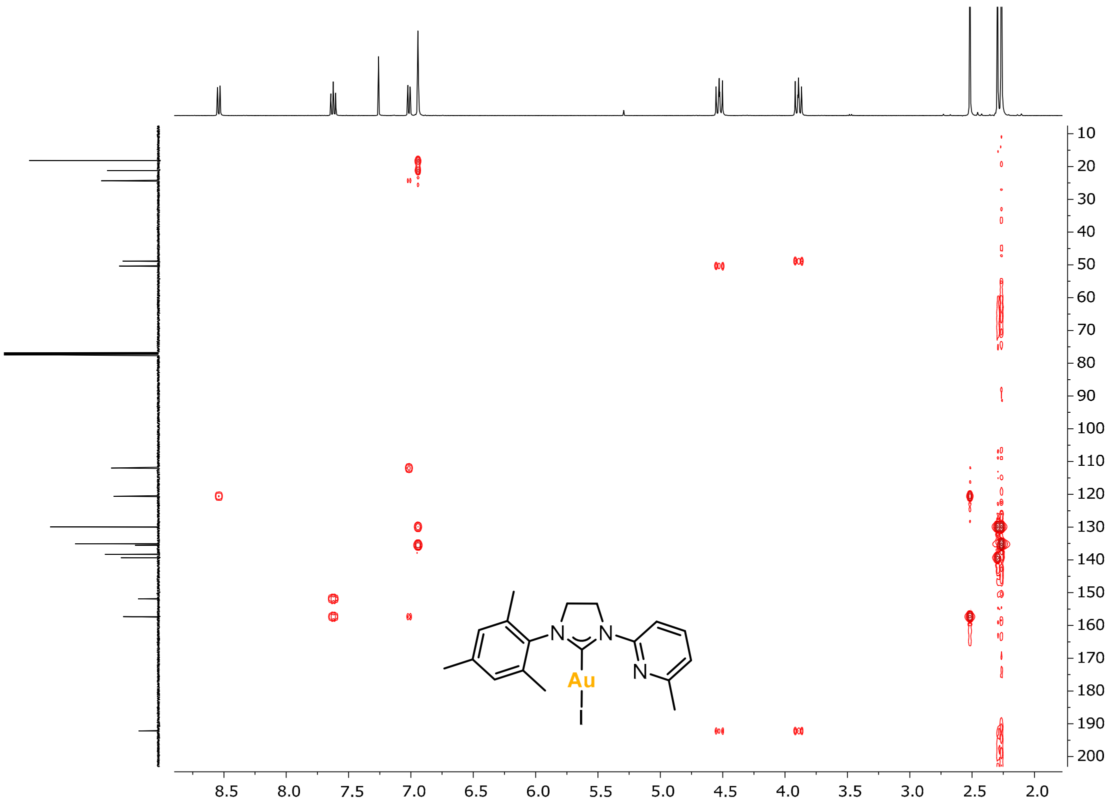

Figure S26. $^{1}H,^{13}C$-HMBC NMR (400MHz, 298K) of **3** in $CDCl_3$.

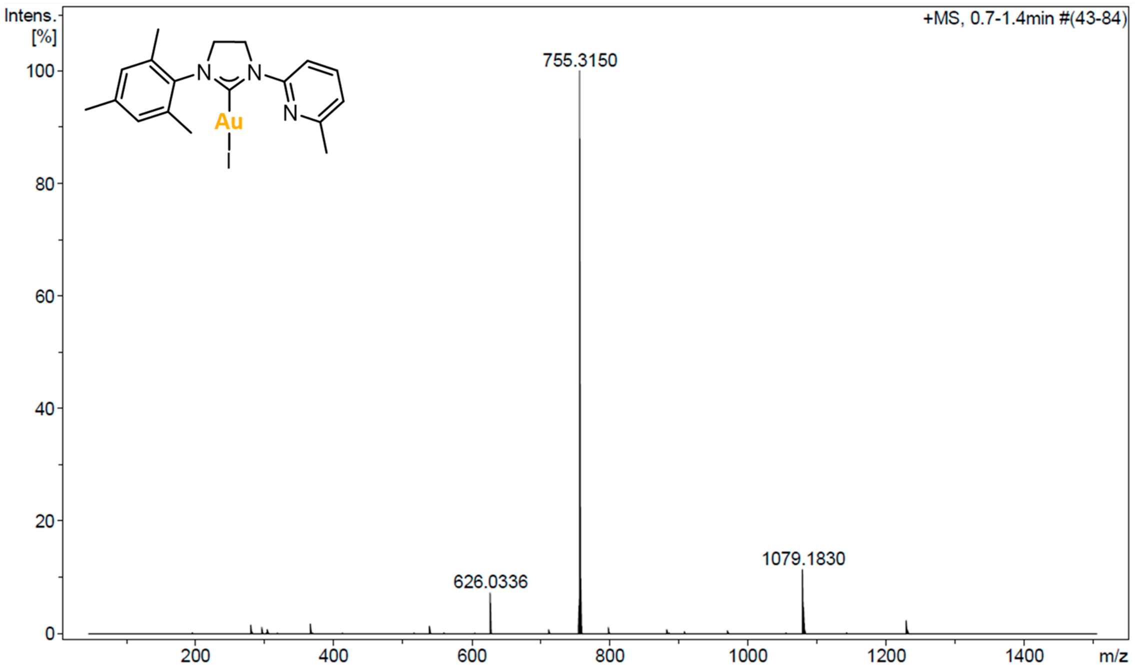

Figure S27. HRMS-ESI(+) of **3**.

S5.6. Complex 4

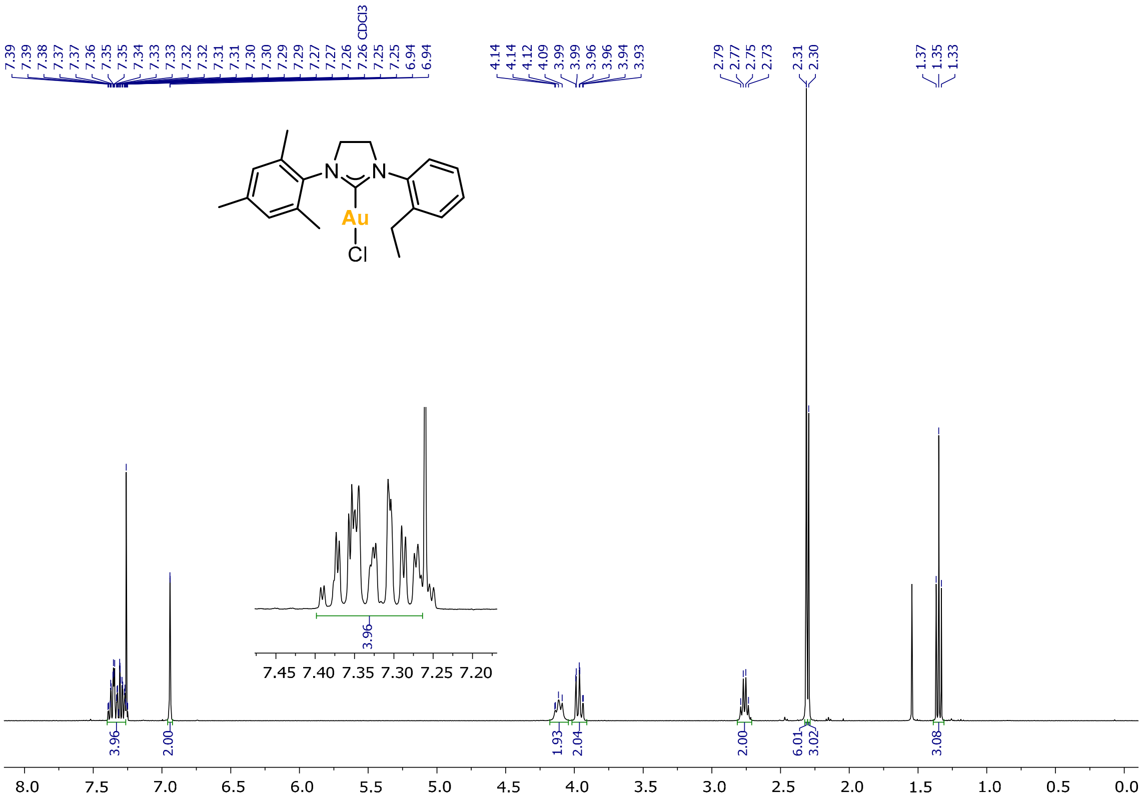

Figure S28. ^{1}H NMR (400MHz, 298K) of **4** in $CDCl_3$.

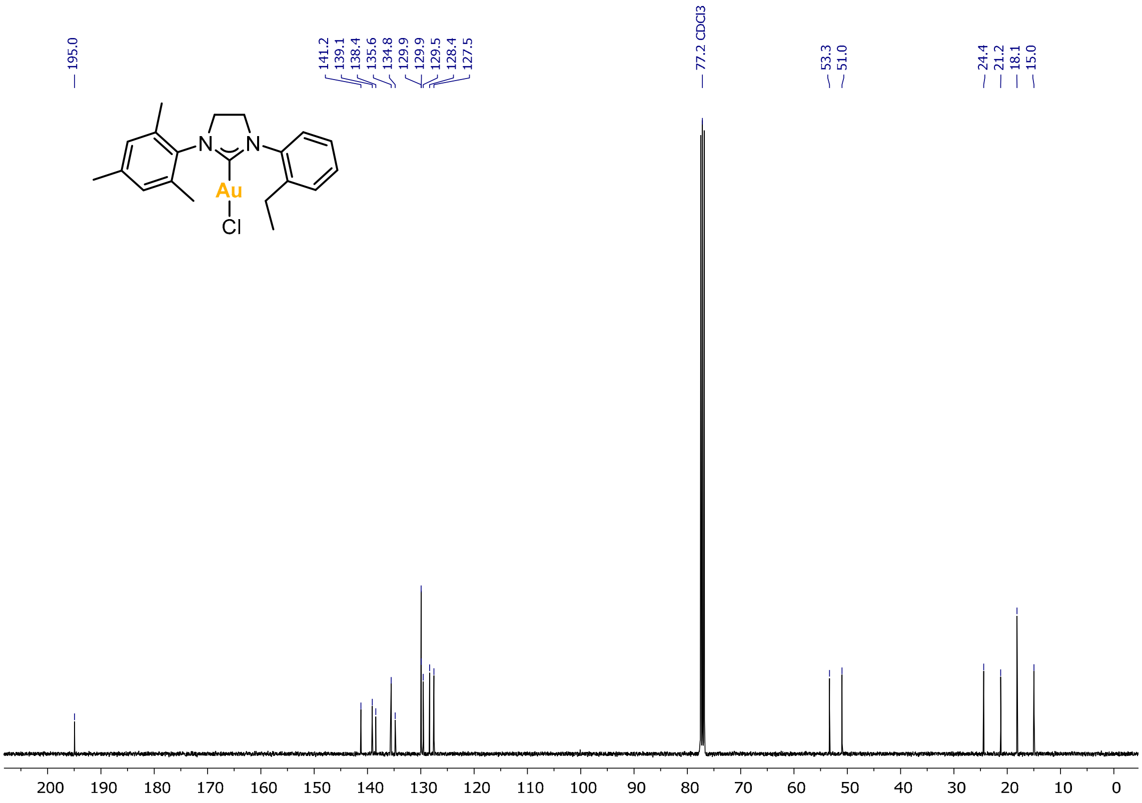

Figure S29. $^{13}C\{^{1}H\}$ NMR (101MHz, 298K) of **4** in $CDCl_3$.

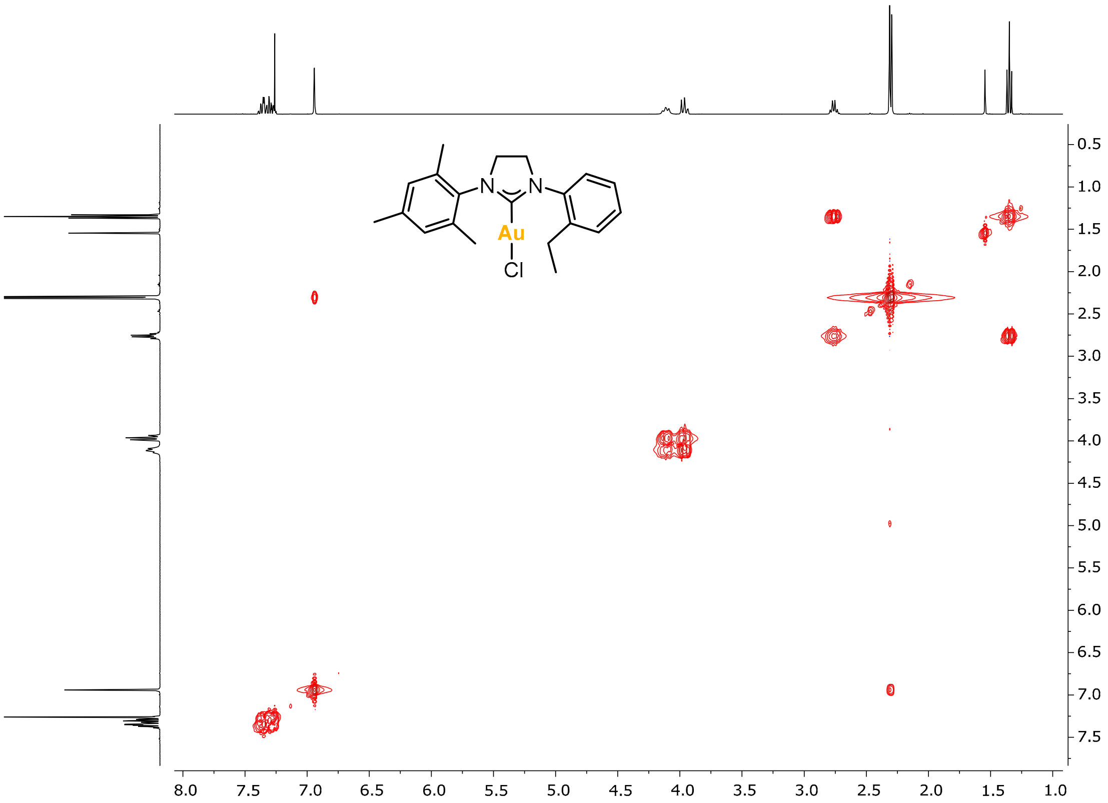

Figure S30. $^{1}H,^{1}H$-COSY NMR (400MHz, 298K) of **4** in $CDCl_3$.

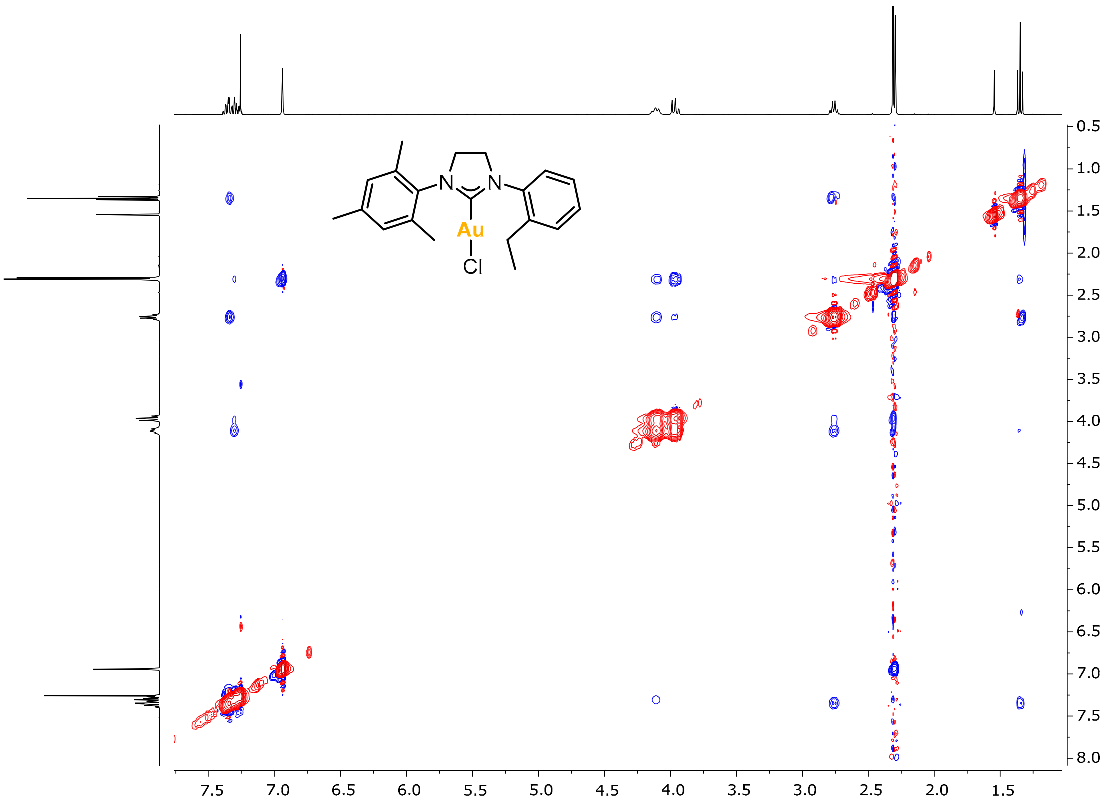

Figure S31. ^{1}H,^{1}H-NOESY NMR (400MHz, 298K) of **4** in $CDCl_3$.

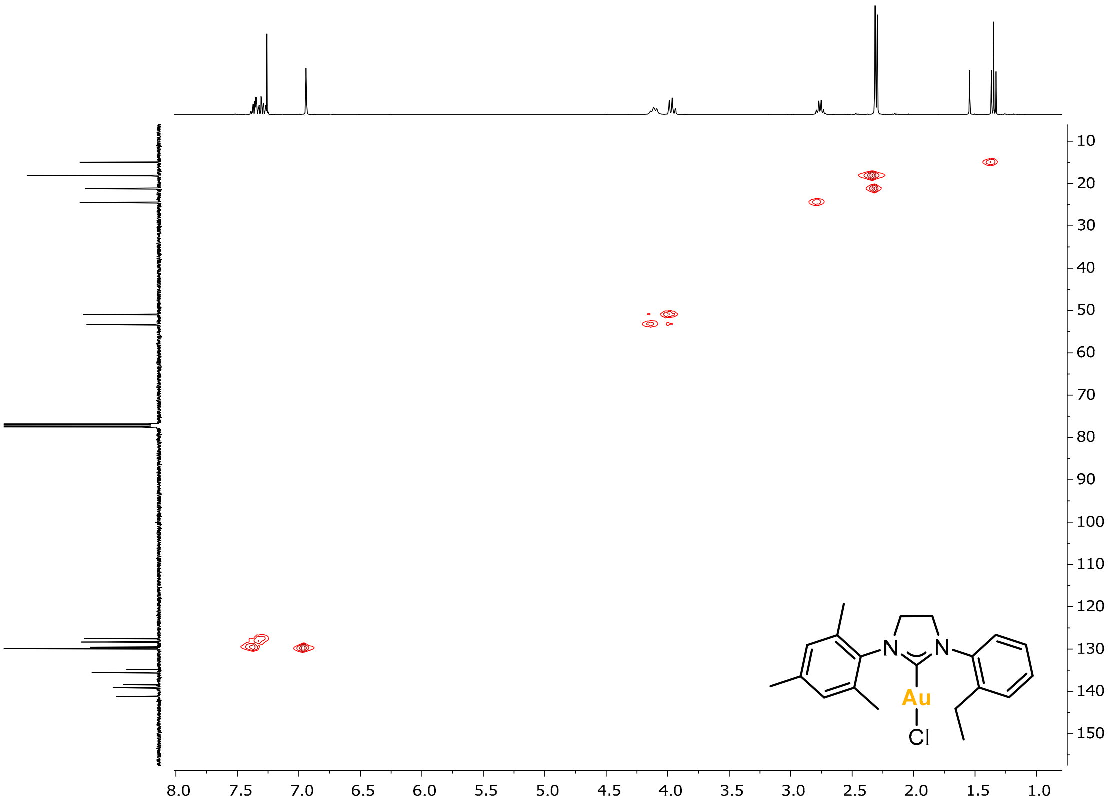

Figure S32. ^{1}H,^{13}C-HSQC NMR (400MHz, 298K) of **4** in $CDCl_3$.

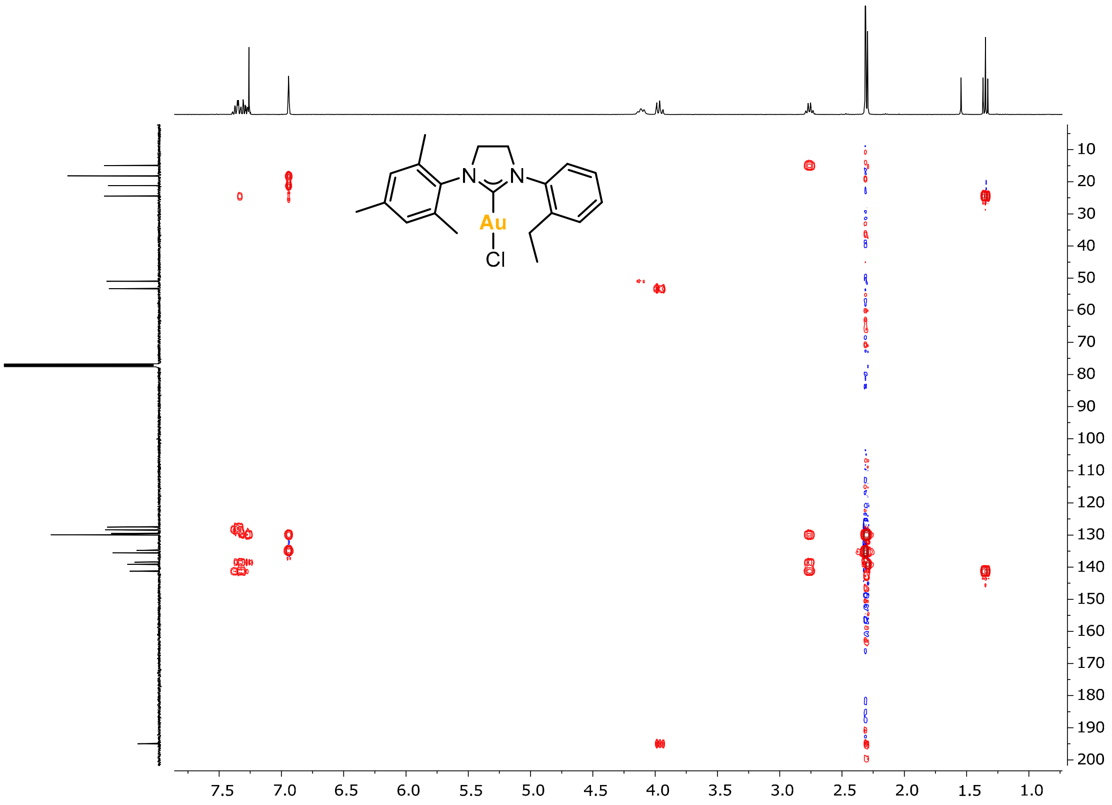

Figure S33. $^{1}H,^{13}C$-HMBC NMR (400MHz, 298K) of **4** in $CDCl_3$.

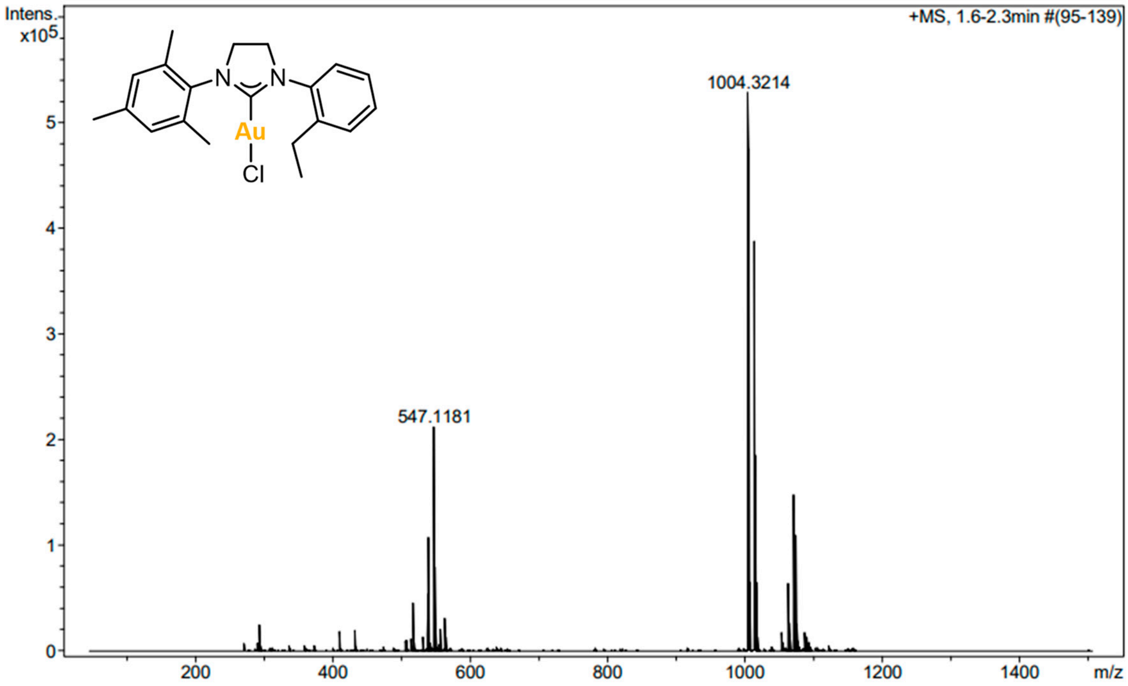

Figure S34. HRMS-ESI(+) of **4**.

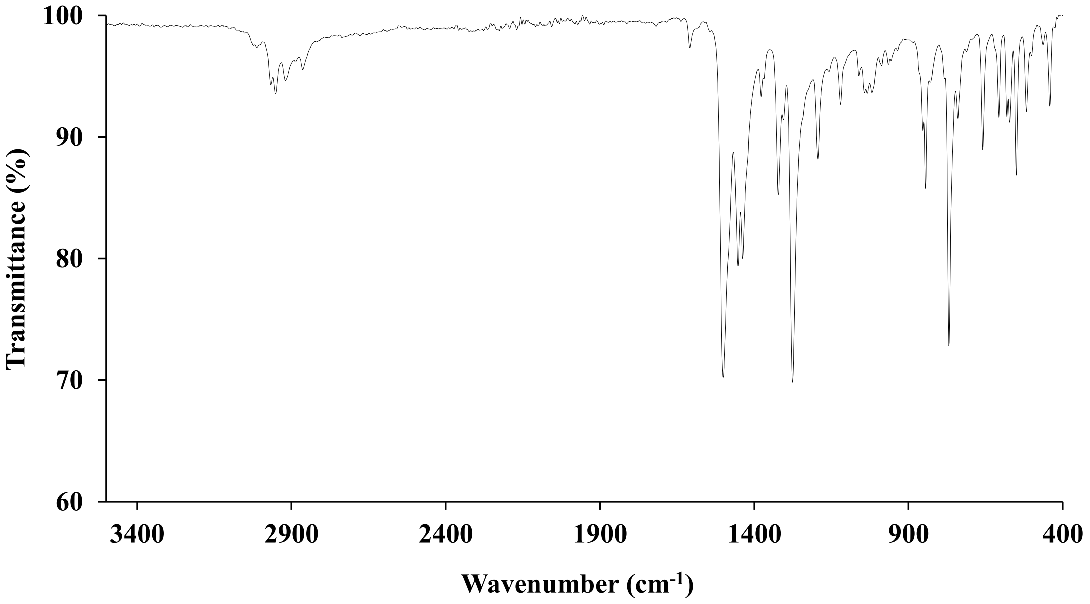

Figure S35. IR spectrum of **4**.

S5.7. Compound L1^{ox}-I

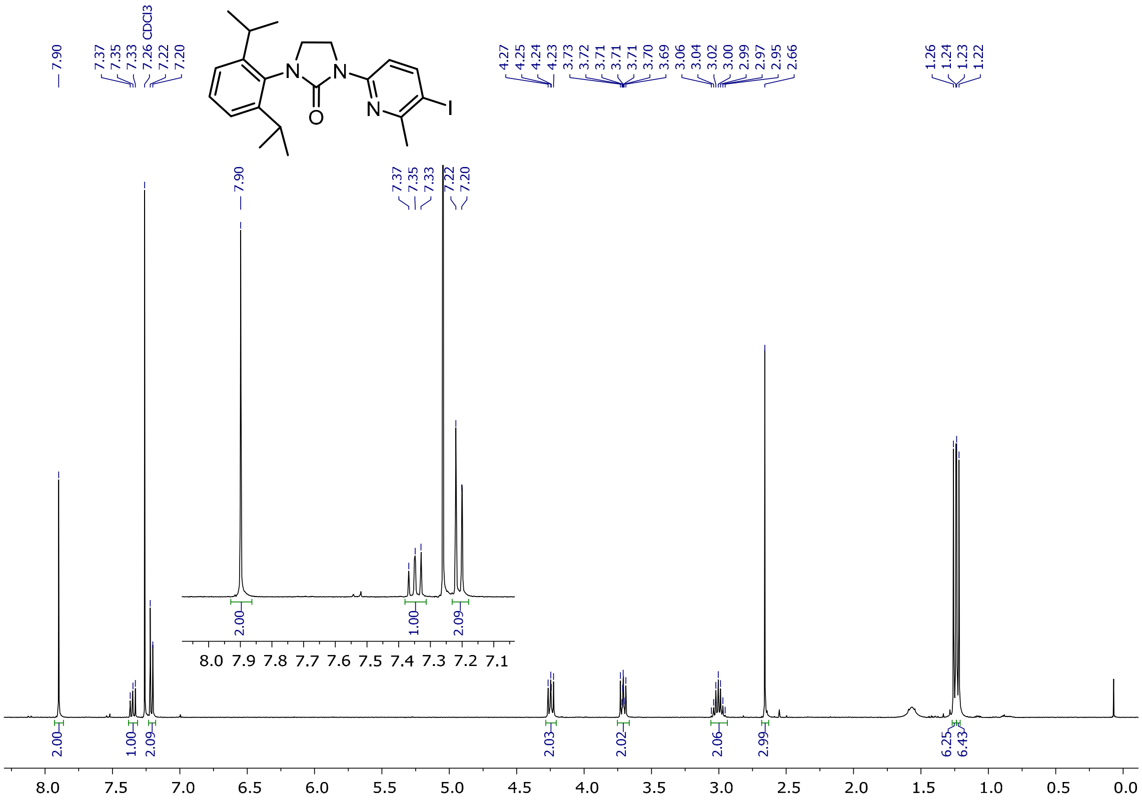

Figure S36. ^{1}H NMR (400MHz, 298K) of **L1^{ox}-I** in $CDCl_3$.

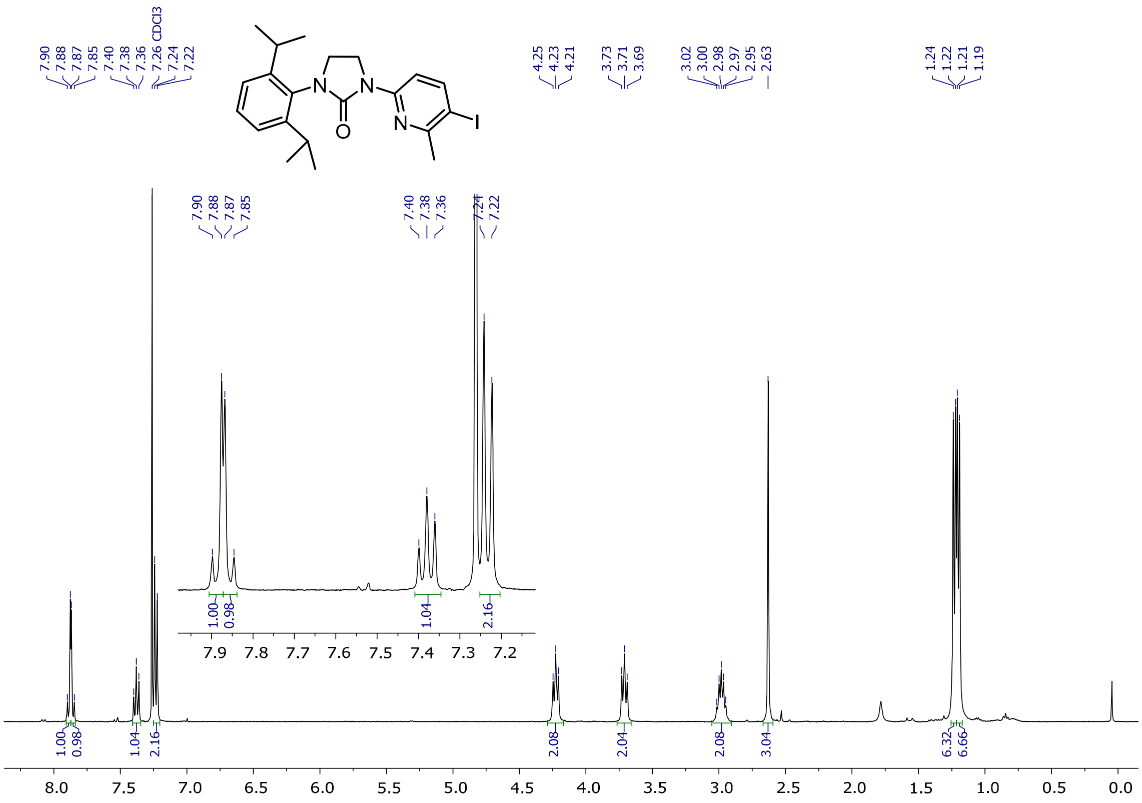

Figure S37. ^{1}H NMR (400MHz, 228K) of **L1^{ox}-I** in $CDCl_3$.

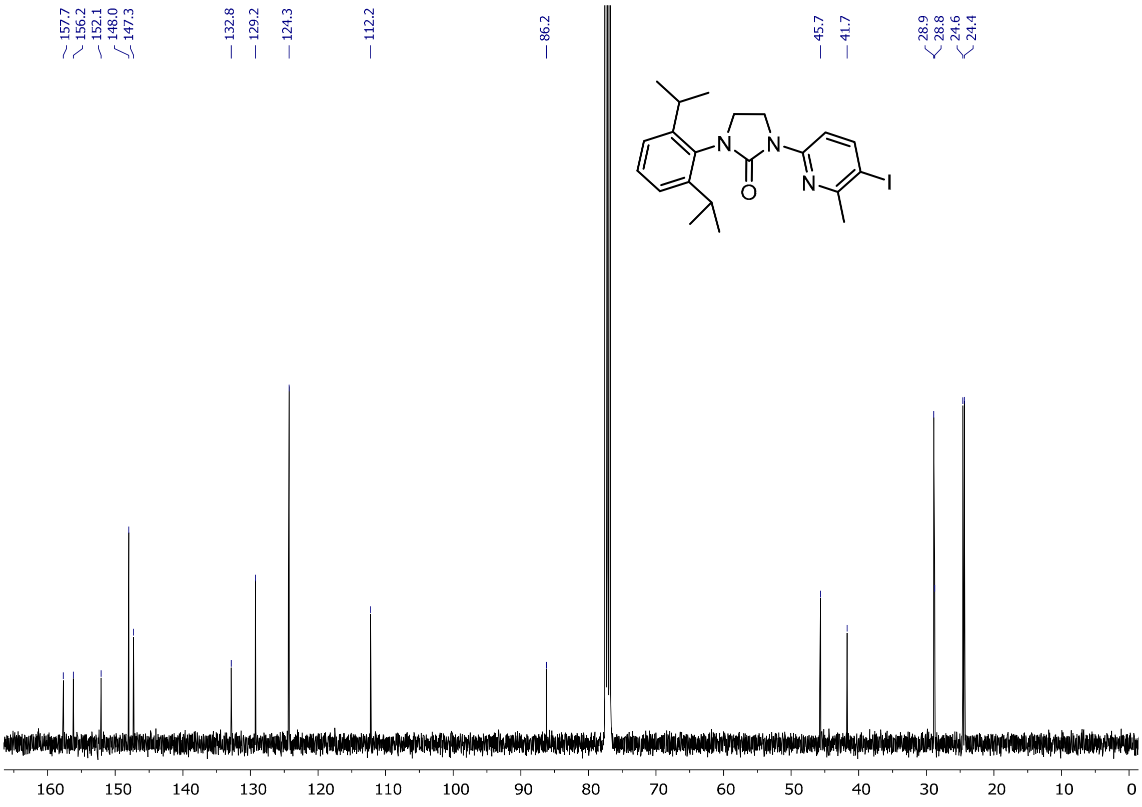

Figure S38. $^{13}C\{^{1}H\}$ NMR (101MHz, 298K) of **L1^{ox}-I** in $CDCl_3$.

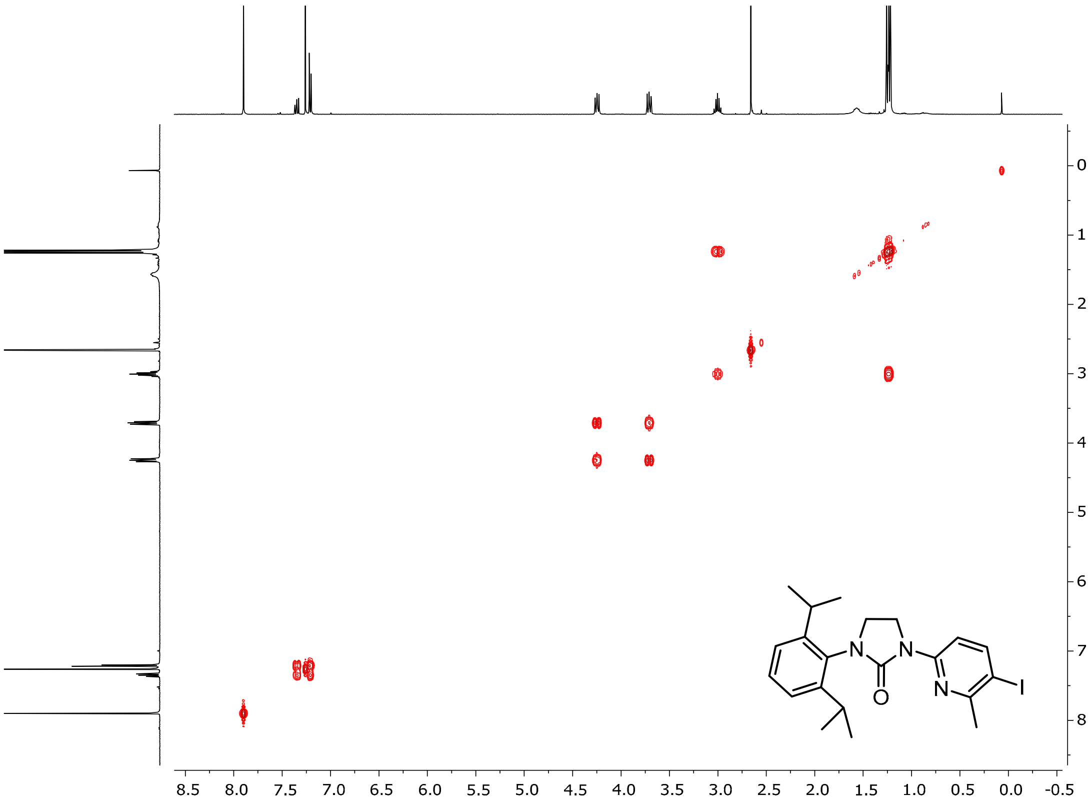

Figure S39. ^{1}H,^{1}H-COSY NMR (400MHz, 298K) of **L1^{ox}-I** in $CDCl_3$.

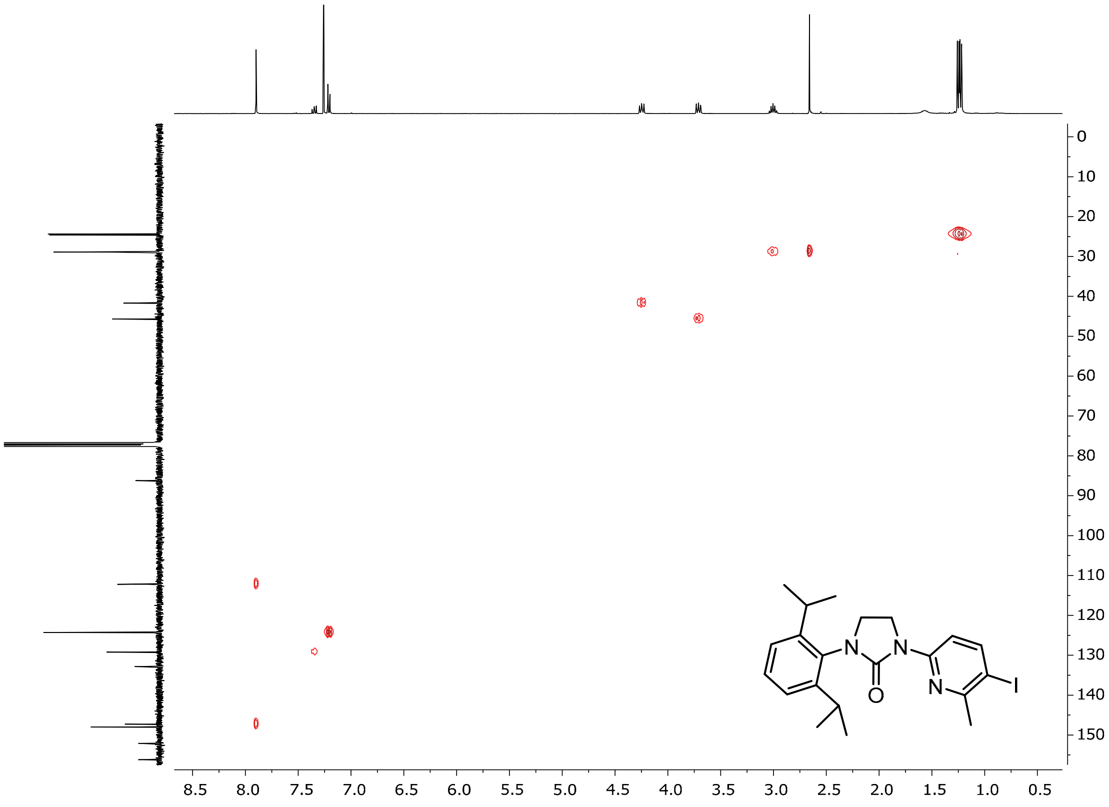

Figure S40. ^{1}H,^{13}C-HSQC NMR (400MHz, 298K) of **L1^{ox}-I** in $CDCl_3$.

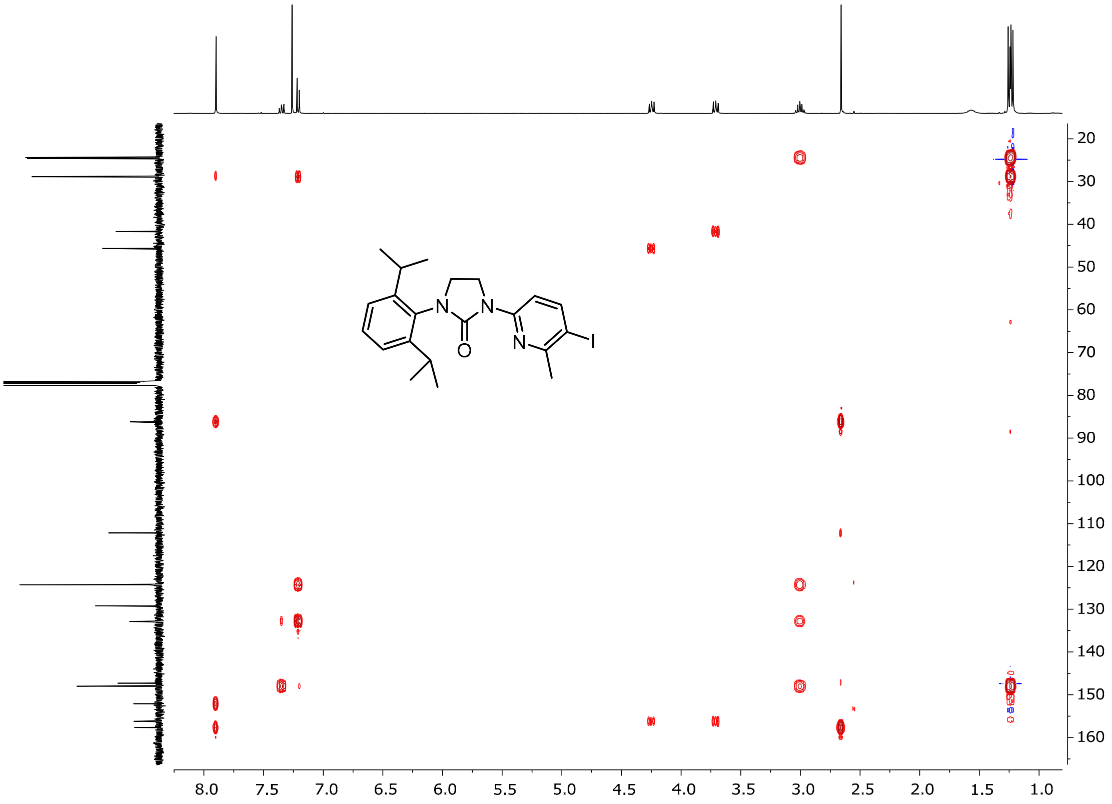

Figure S41. ^{1}H,^{13}C-HMBC NMR (400MHz, 298K) of **L1^{ox}-I** in $CDCl_3$.

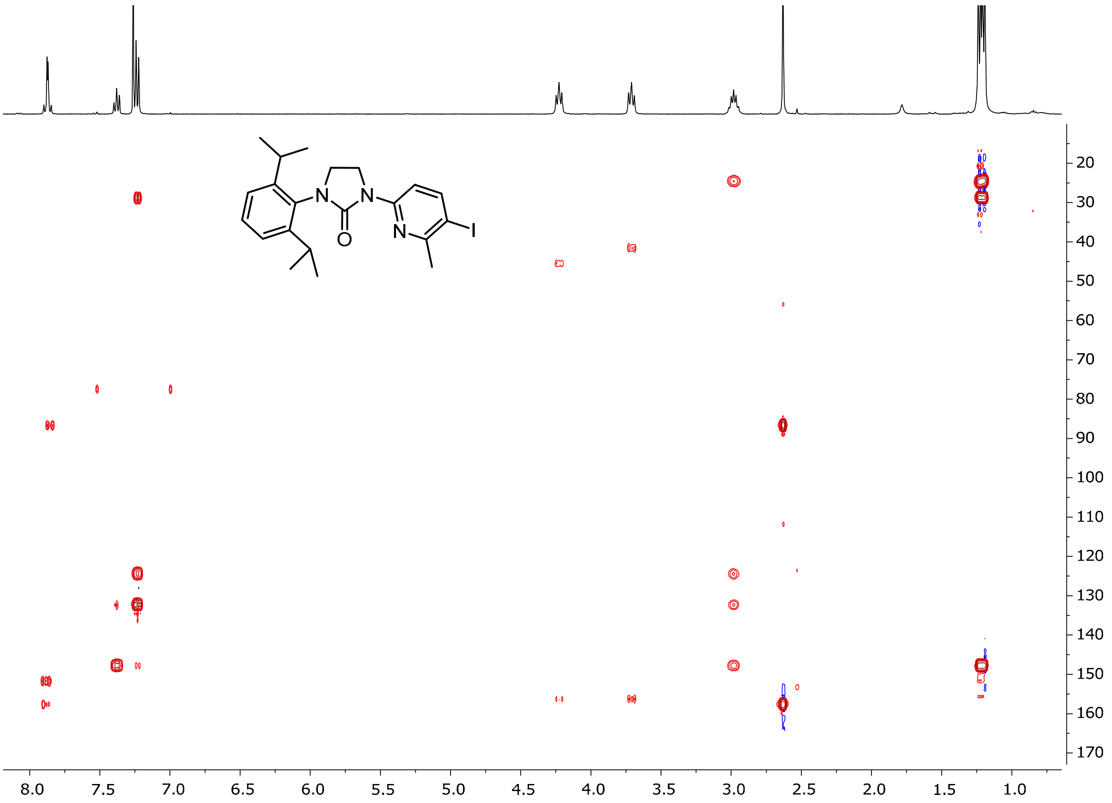

Figure S42. ^{1}H,^{13}C-HMBC NMR (400MHz, 228K) of **L1^{ox}-I** in $CDCl_3$.

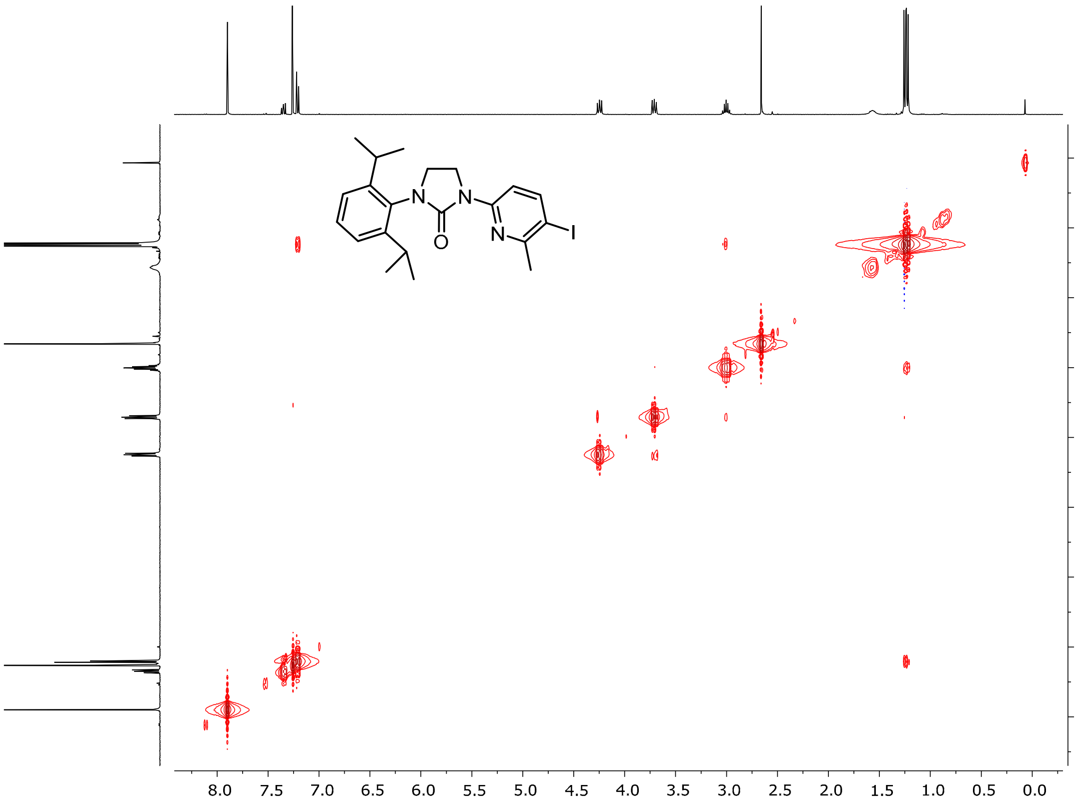

Figure S43. ^{1}H,^{1}H-NOESY NMR (400MHz, 298K) of **L1^{ox}-I** in $CDCl_3$.

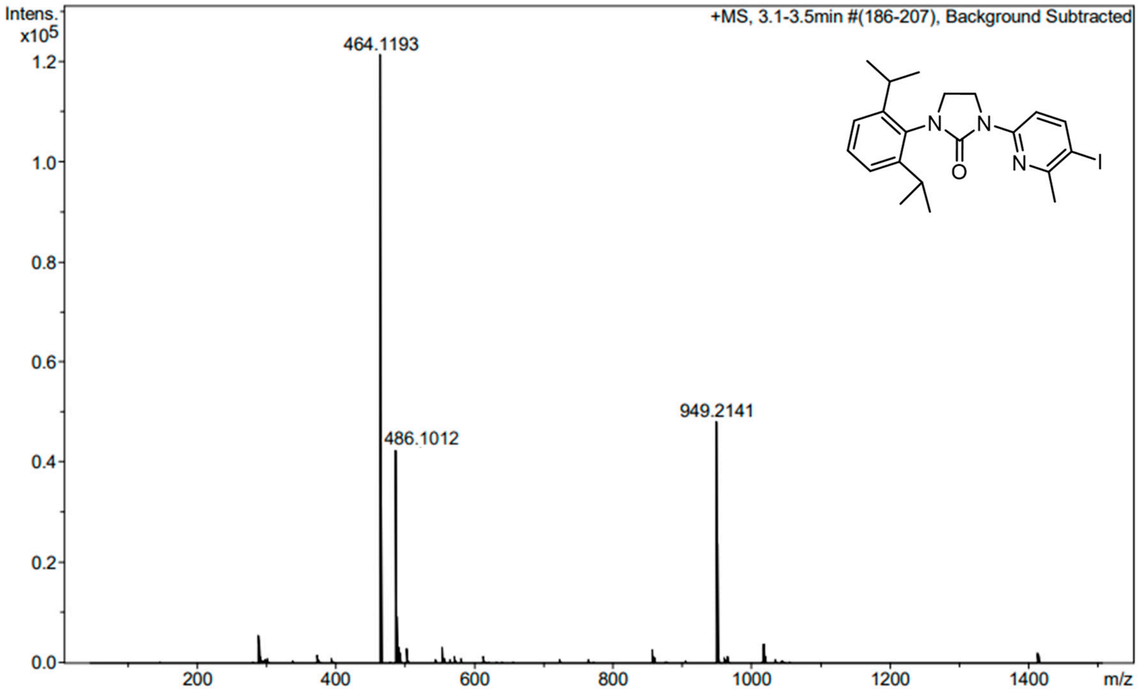

Figure S44. HRMS-ESI(+) of **L1^{ox}-I**.

S5.8. Compound L2^{ox}

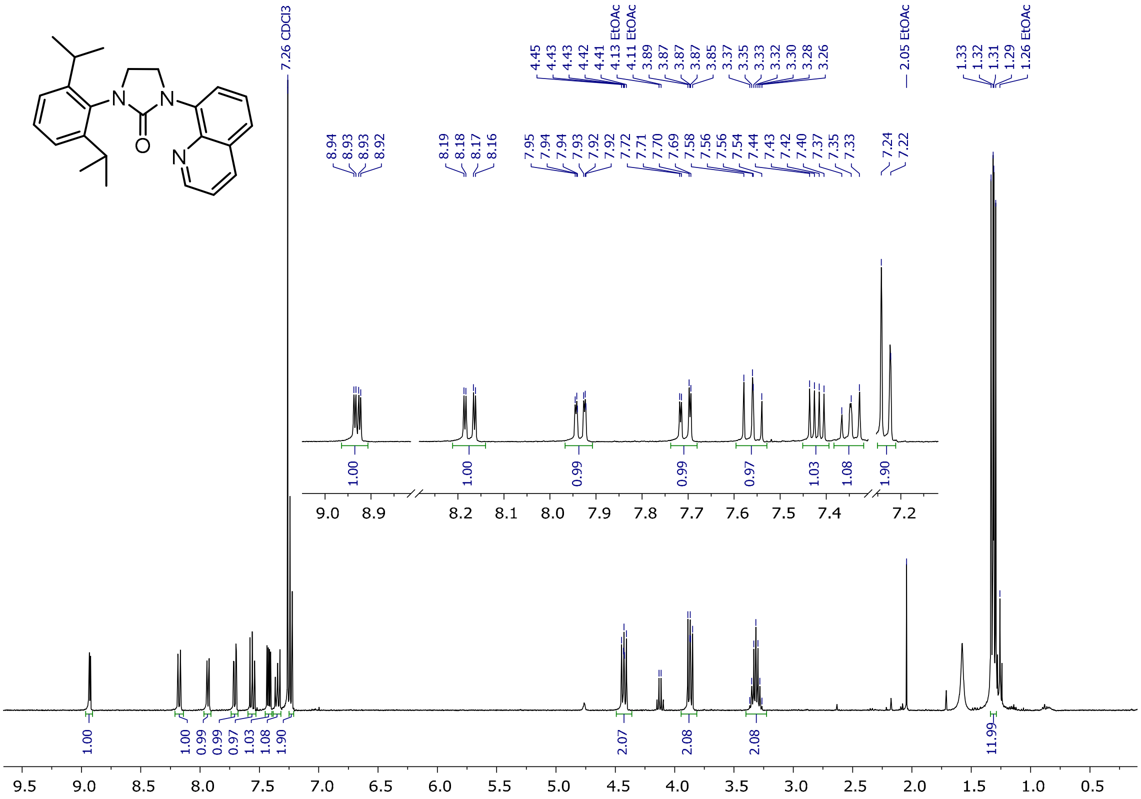

Figure S45. ^{1}H NMR (400MHz, 298K) of **L2^{ox}** in $CDCl_3$.

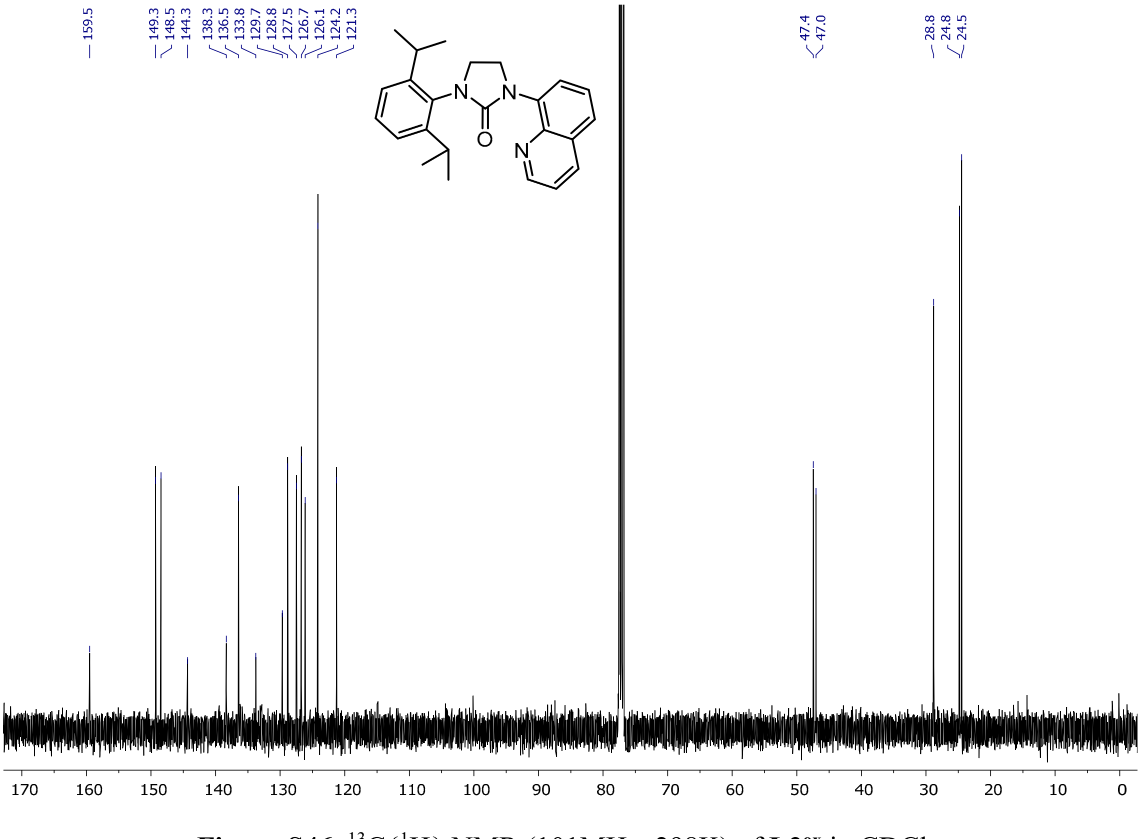

Figure S46. $^{13}C\{^{1}H\}$ NMR (101MHz, 298K) of **L2^{ox}** in $CDCl_3$.

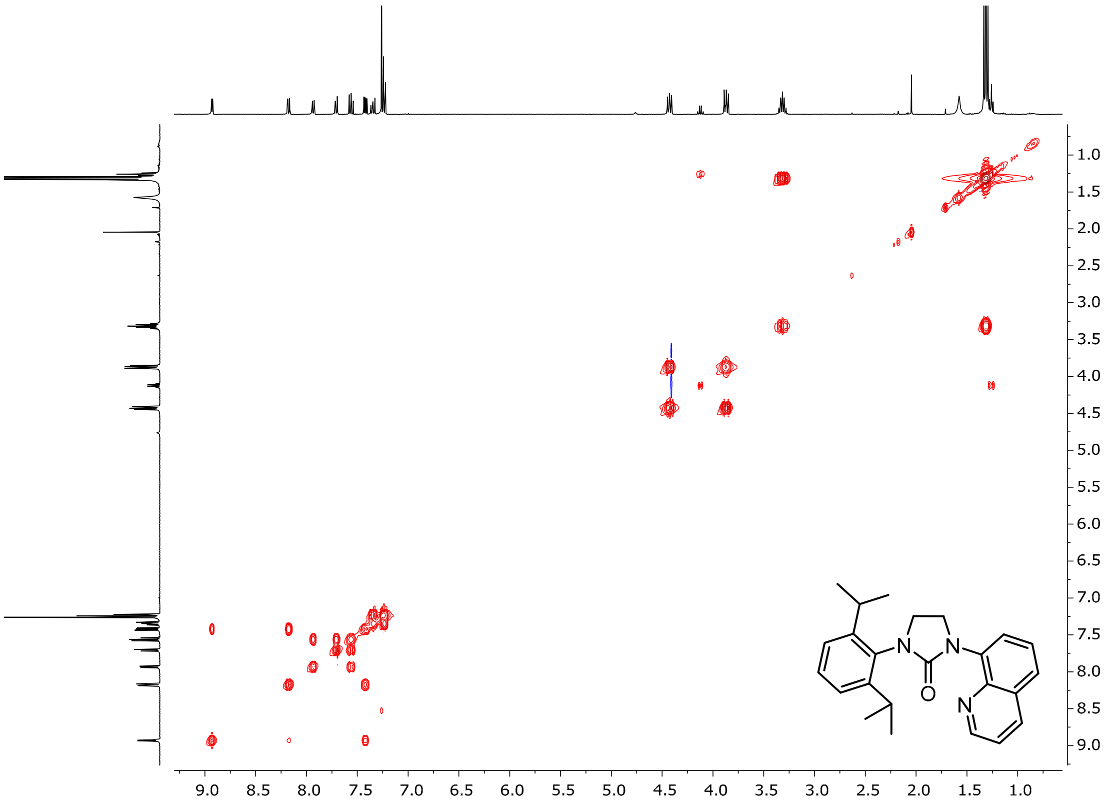

Figure S47. ^{1}H,^{1}H-COSY NMR (400MHz, 298K) of **L2^{ox}** in $CDCl_3$.

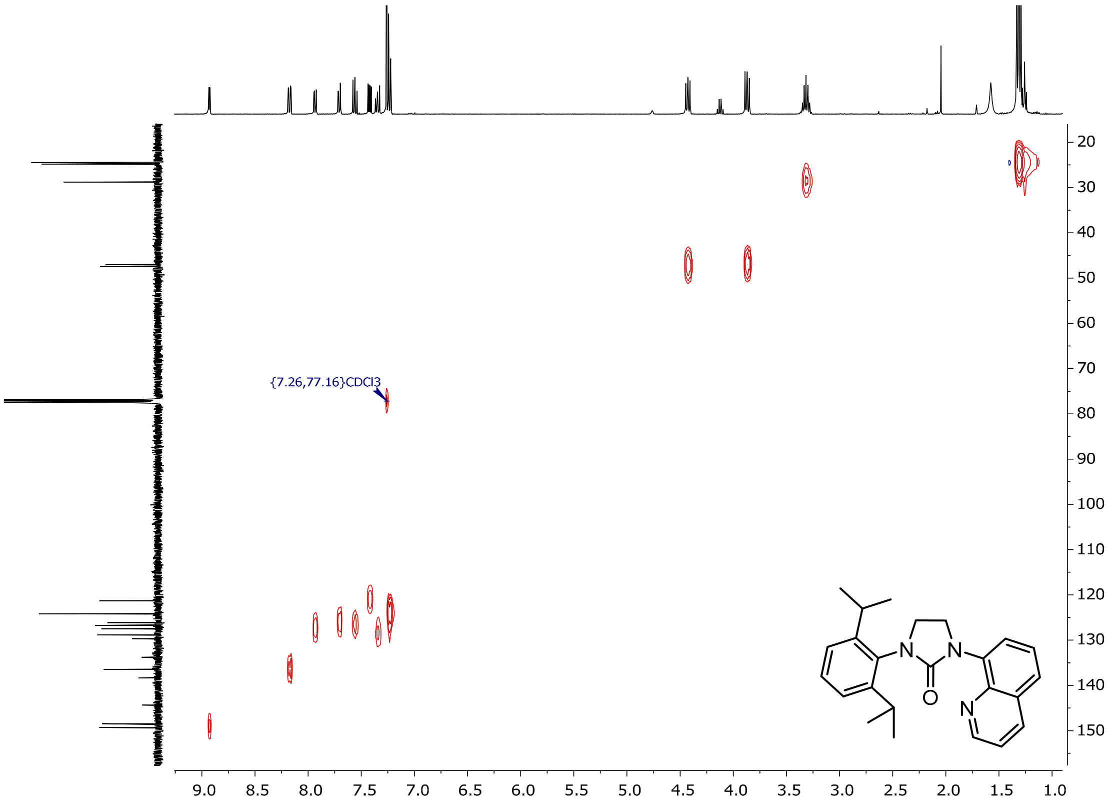

Figure S48. ^{1}H,^{13}C-HSQC NMR (400MHz, 298K) of **L2^{ox}** in $CDCl_3$.

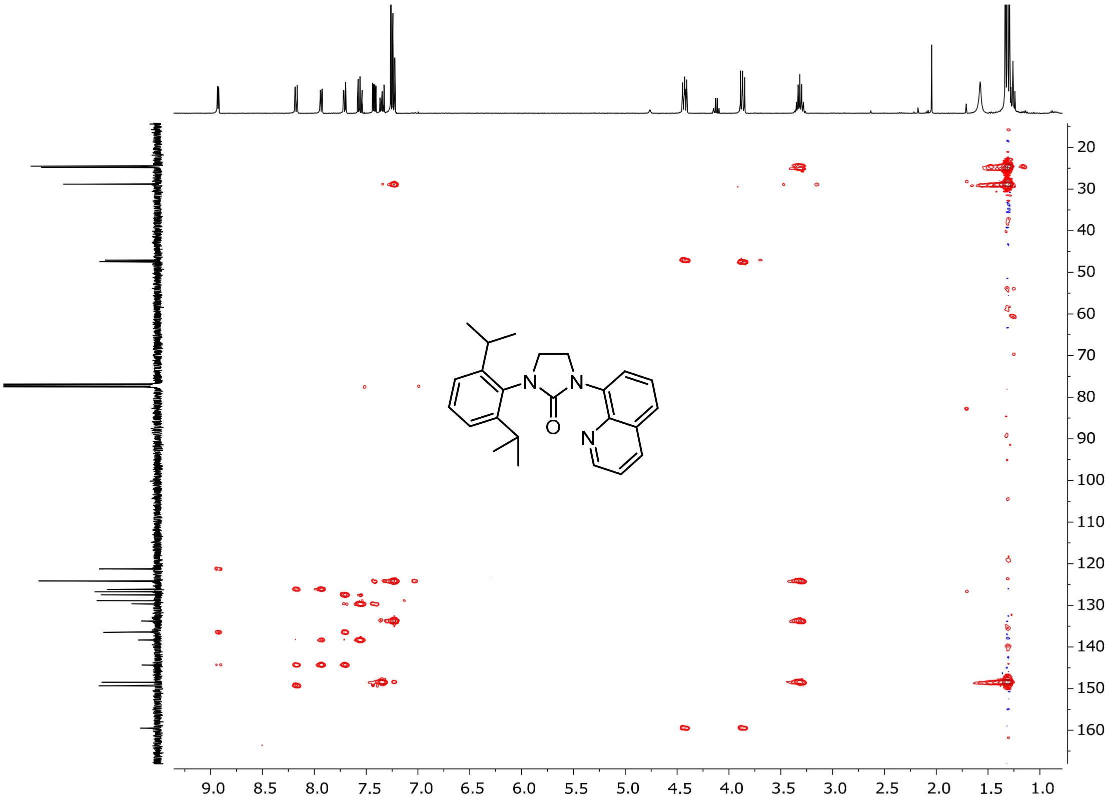

Figure S49. $^{1}H,^{13}C$-HMBC NMR (400MHz, 298K) of **L2**^{ox} in $CDCl_3$.

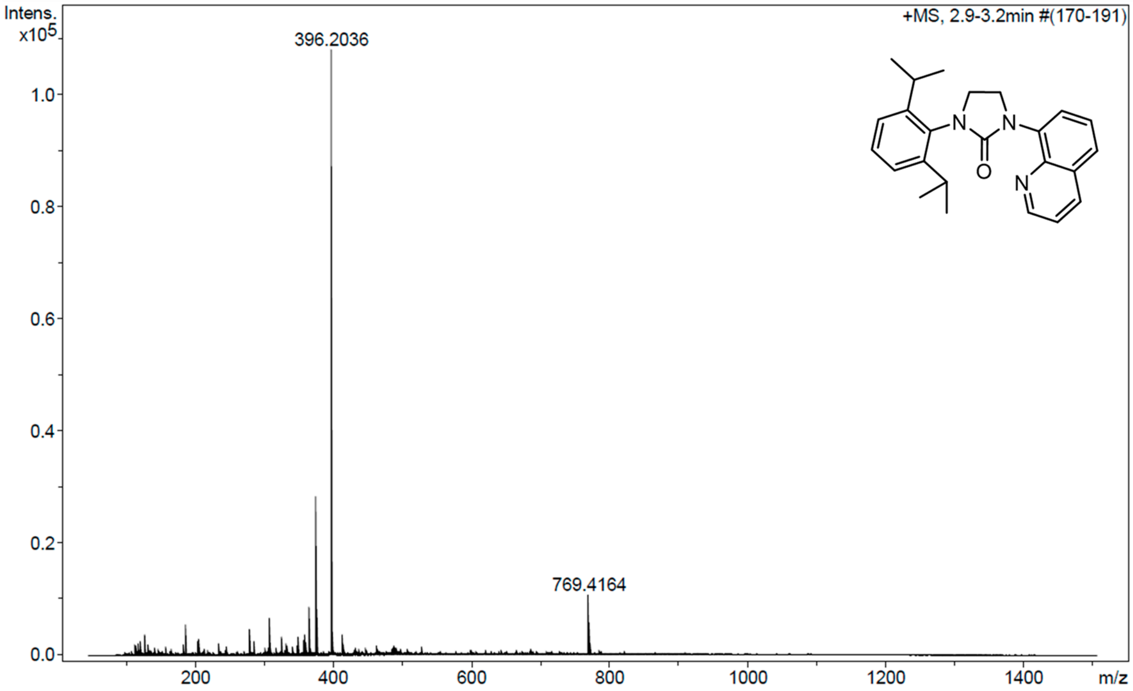

Figure S50. HRMS-ESI(+) of **L2**^{ox}.

S5.9. Compound L2^{ox}-I

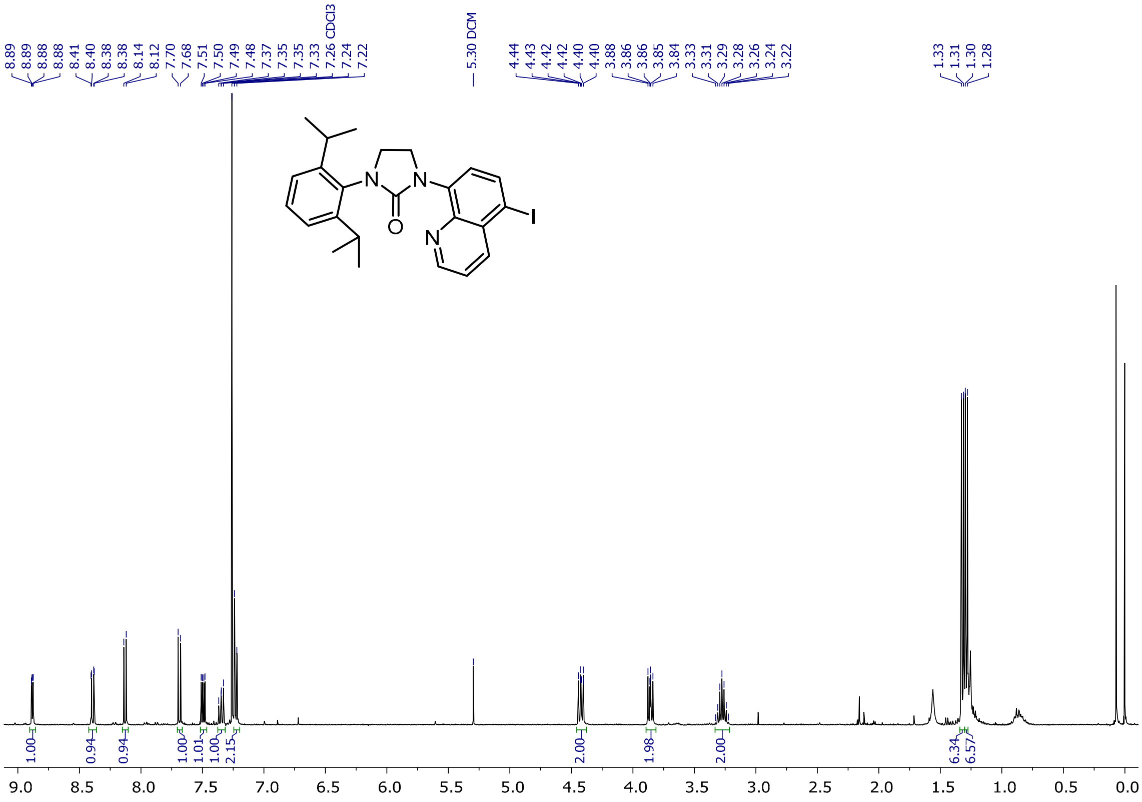

Figure S51. ^{1}H NMR (400MHz, 298K) of **L2^{ox}-I** in $CDCl_3$.

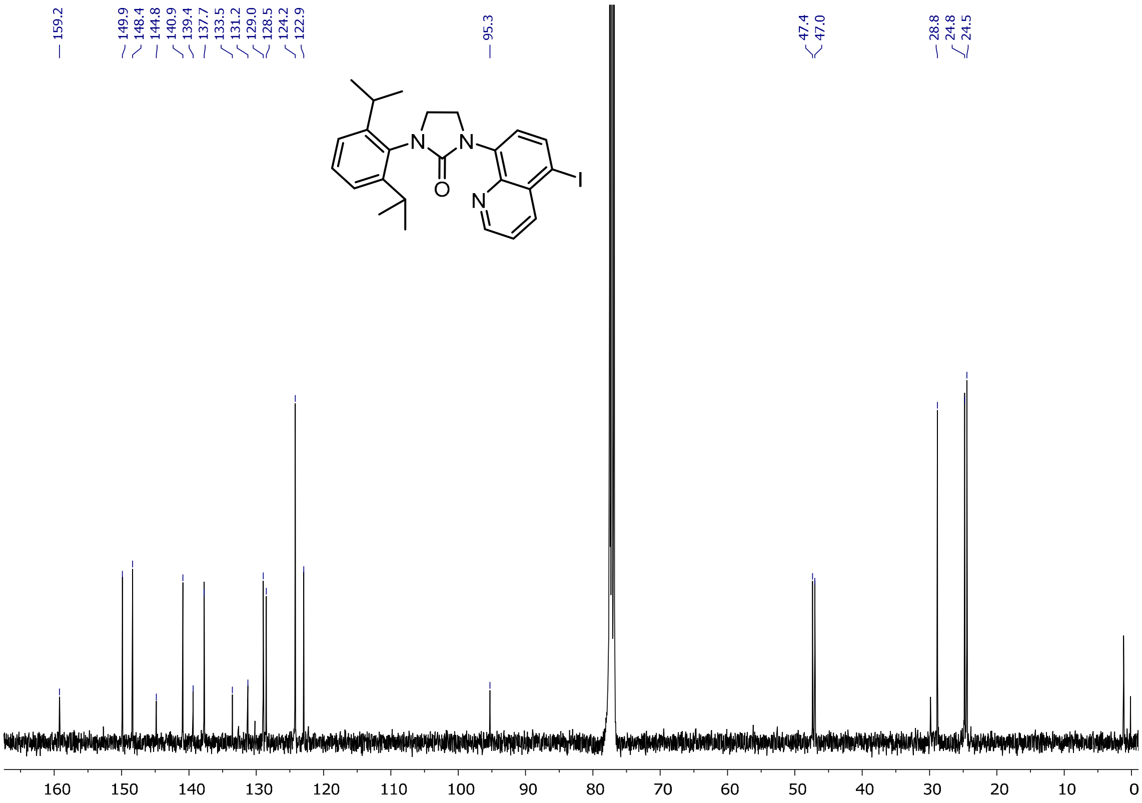

Figure S52. $^{13}C\{^{1}H\}$ NMR (101MHz, 298K) of **L2^{ox}-I** in $CDCl_3$.

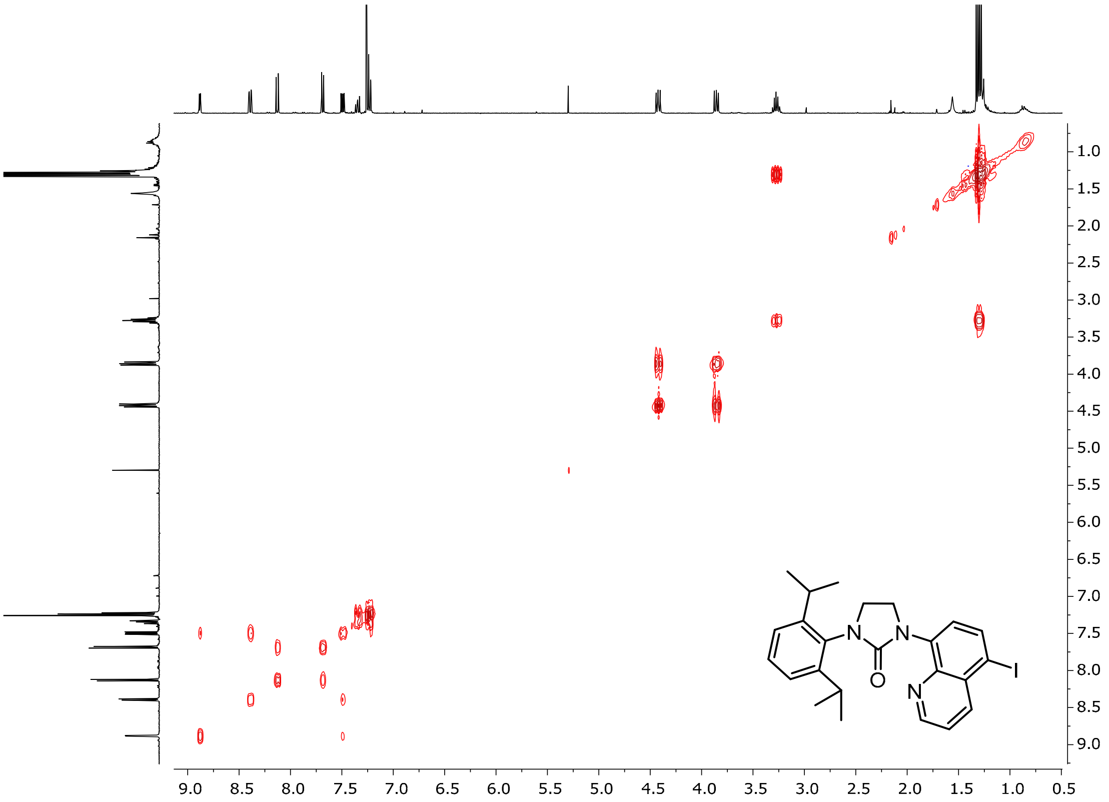

Figure S53. ^{1}H,^{1}H-COSY NMR (400MHz, 298K) of **L2^{ox}-I** in $CDCl_3$.

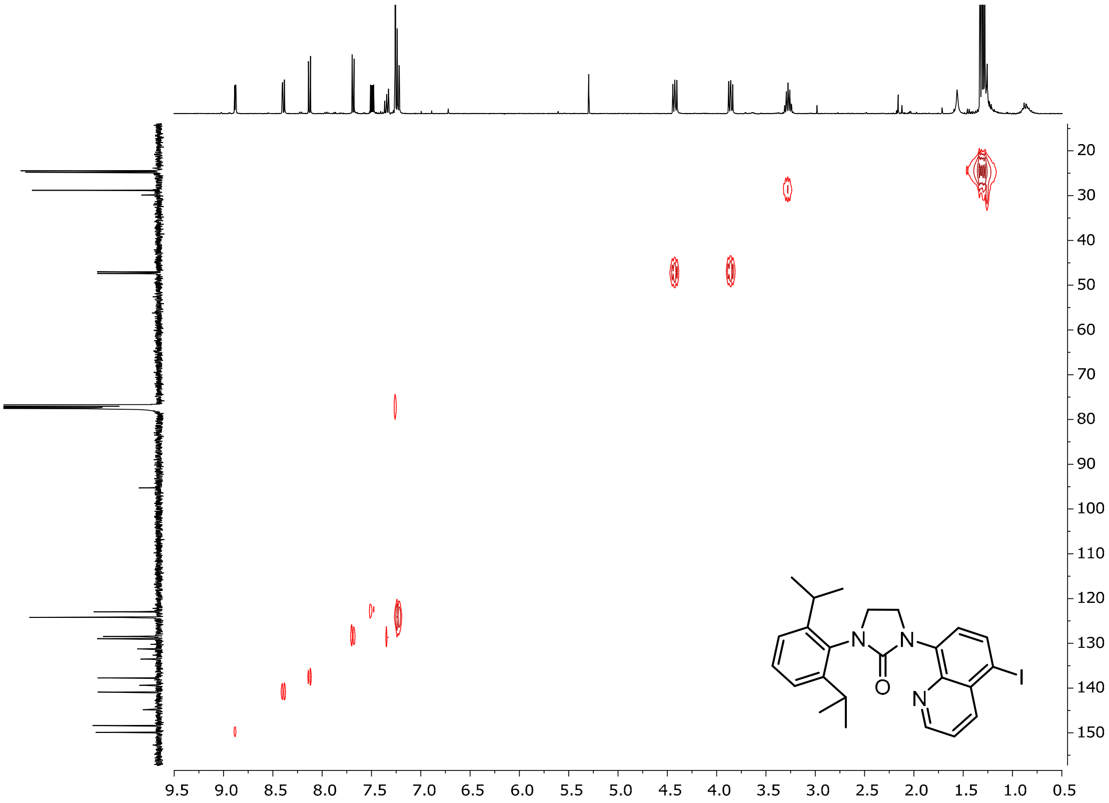

Figure S54. ^{1}H,^{13}C-HSQC NMR (400MHz, 298K) of **L2^{ox}-I** in $CDCl_3$.

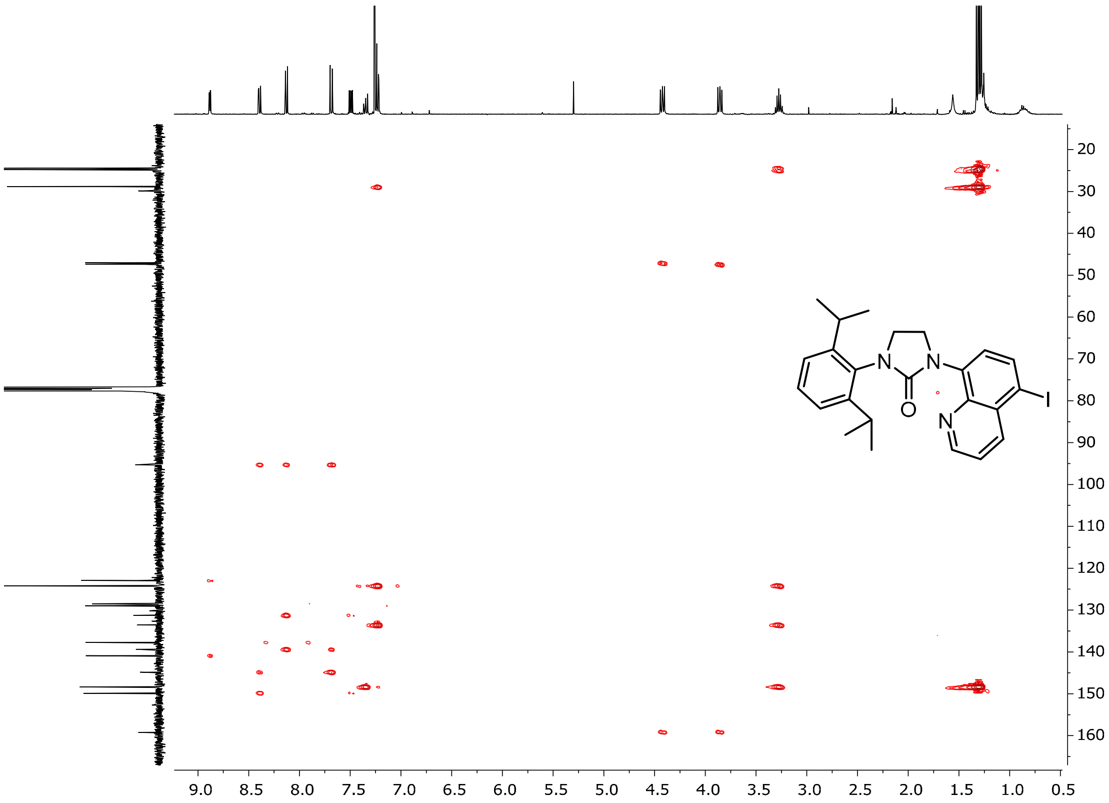

Figure S55. $^{1}H,^{13}C$-HMBC NMR (400MHz, 298K) of **L2^{ox}-I** in $CDCl_3$.

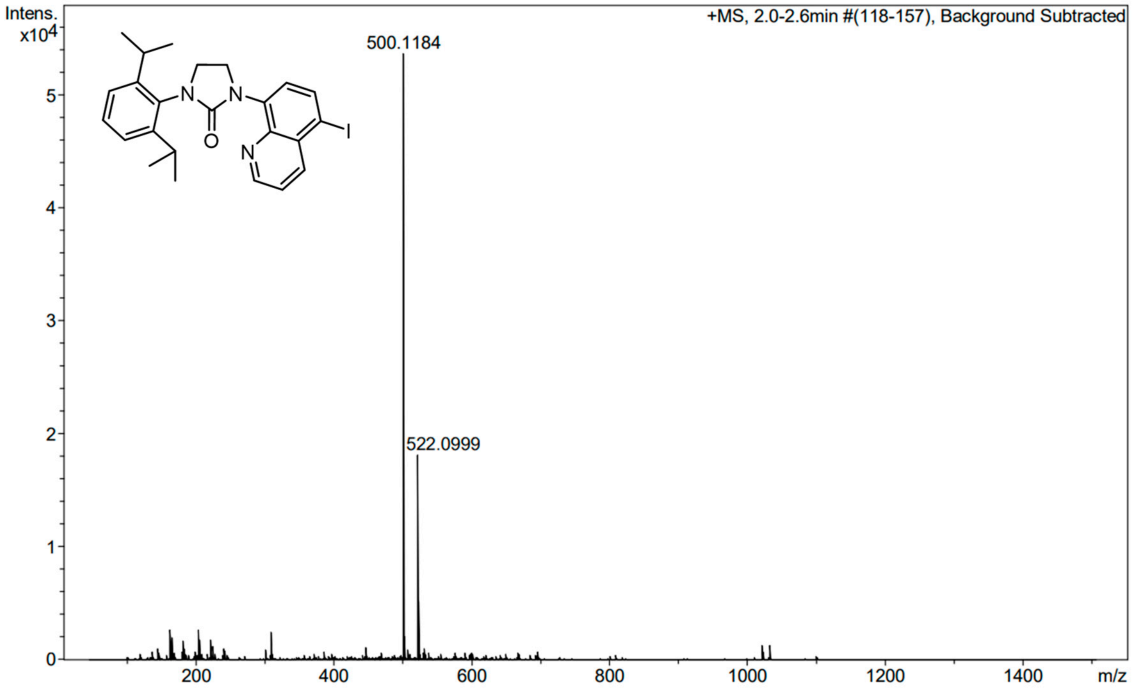

Figure S56. HRMS-ESI(+) of **L2^{ox}-I**.

S5.10. Compound L3^{ox}

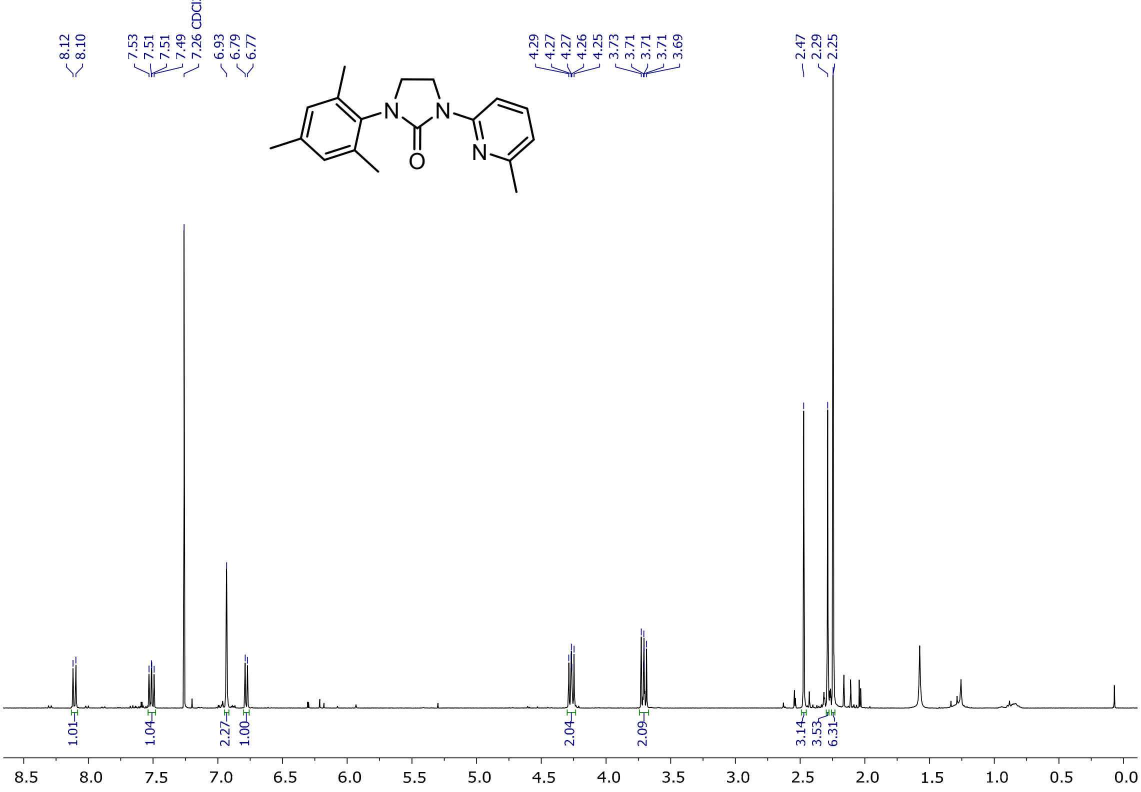

Figure S57. ^{1}H NMR (400MHz, 298K) of **L3^{ox}** in $CDCl_3$.

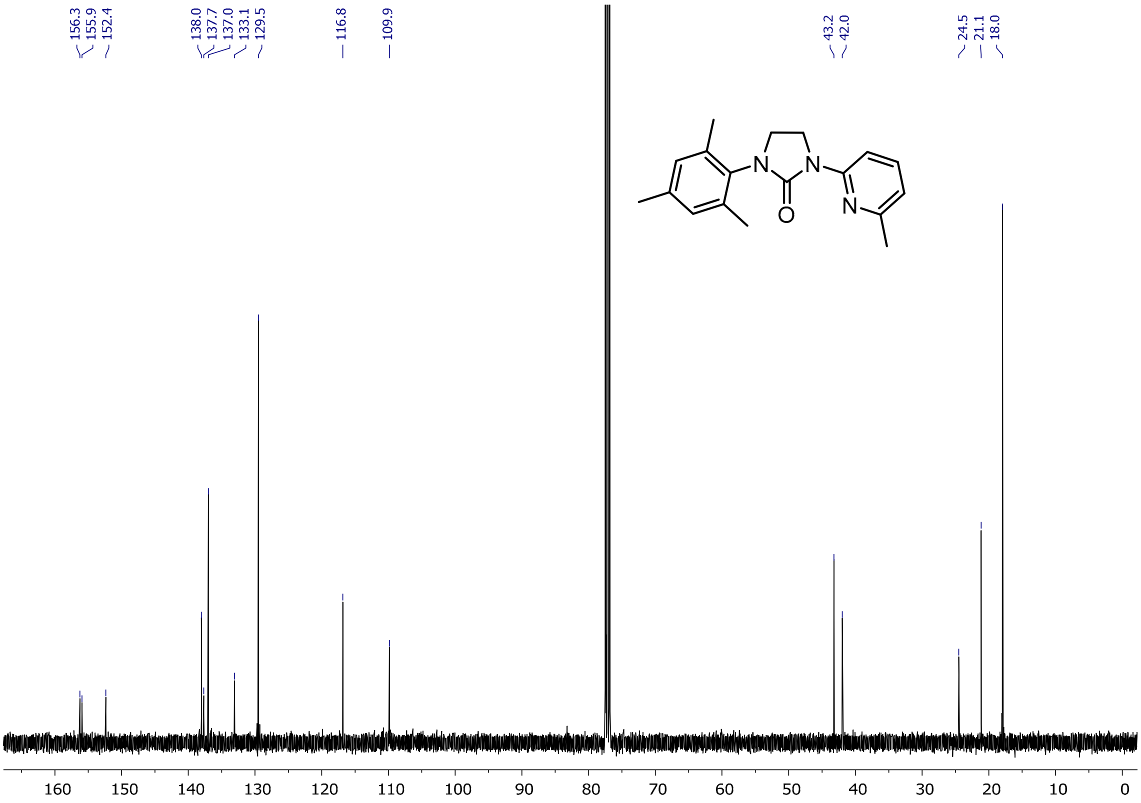

Figure S58. $^{13}C\{^{1}H\}$ NMR (101MHz, 298K) of **L3^{ox}** in $CDCl_3$.

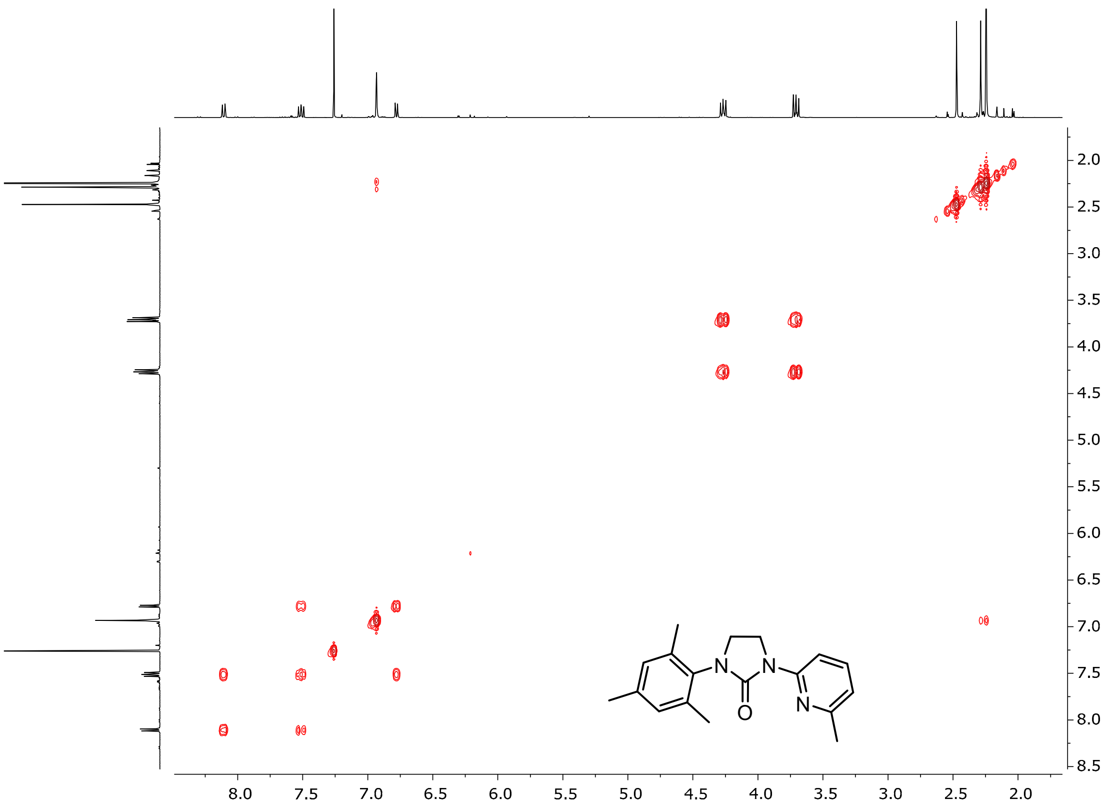

Figure S59. $^{1}H,^{1}H$-COSY NMR (400MHz, 298K) of **L3^{ox}** in $CDCl_3$.

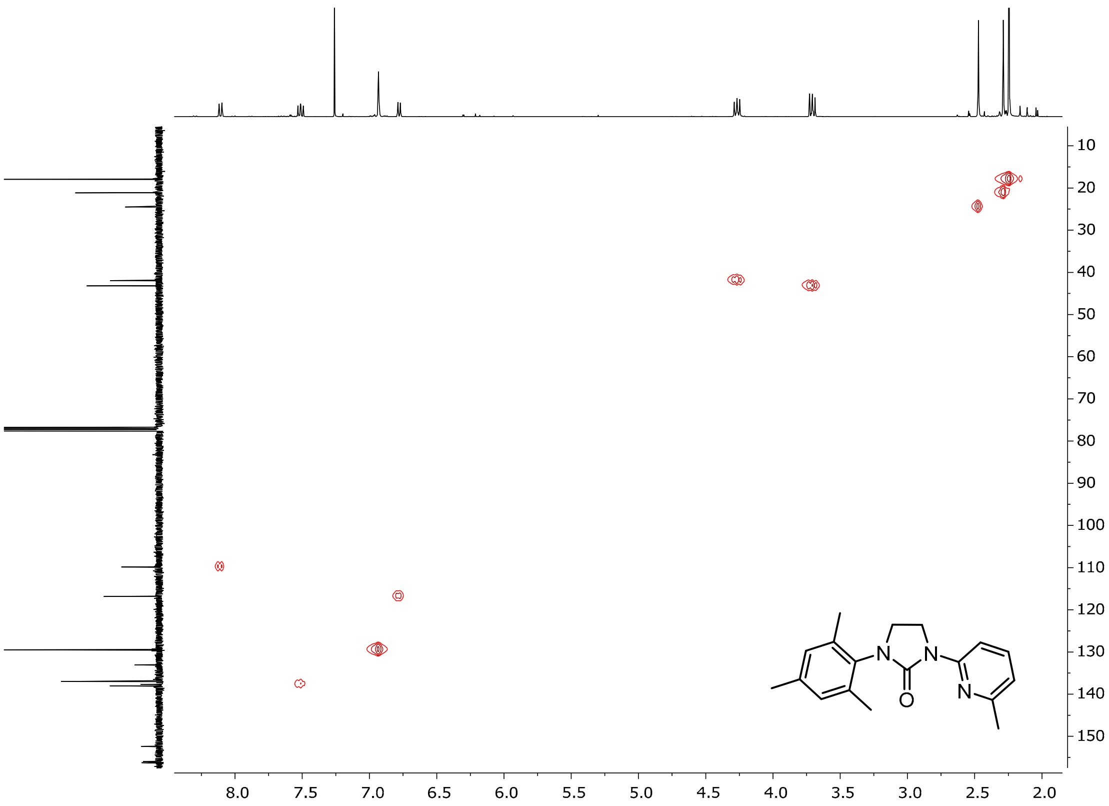

Figure S60. $^{1}H,^{13}C$-HSQC NMR (400MHz, 298K) of **L3^{ox}** in $CDCl_3$.

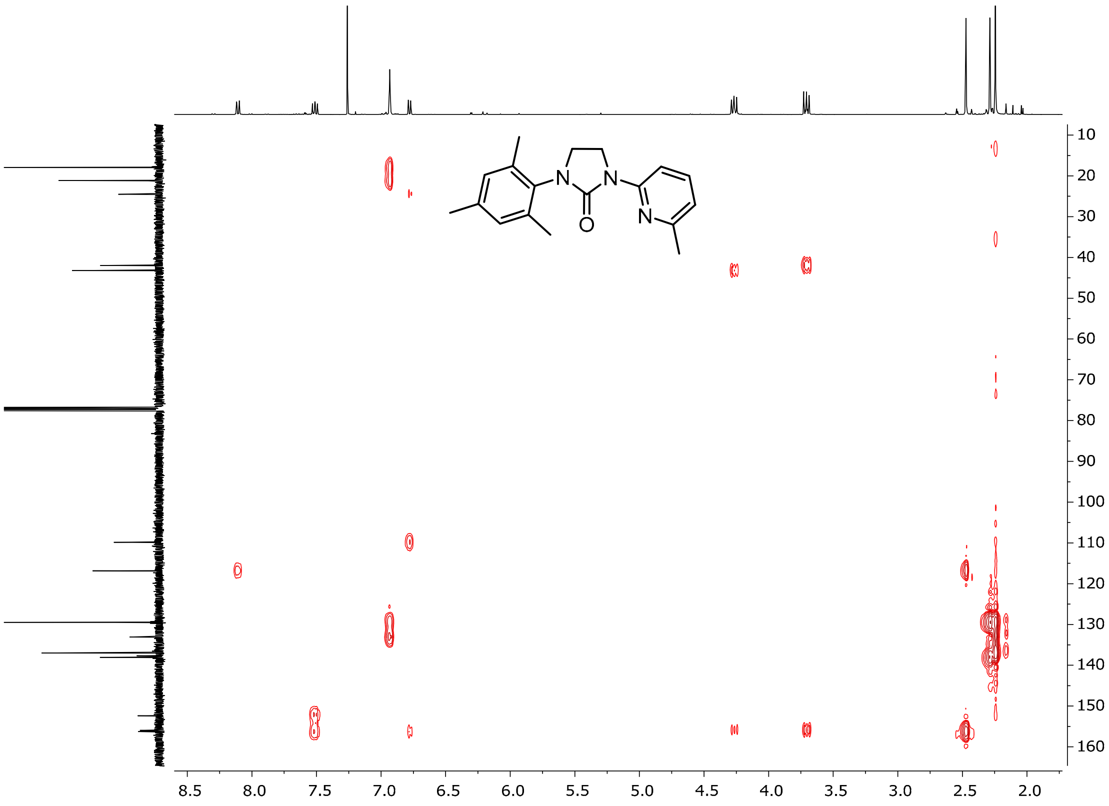

Figure S61. $^{1}H,^{13}C$-HMBC NMR (400MHz, 298K) of **L3^{ox}** in $CDCl_3$.

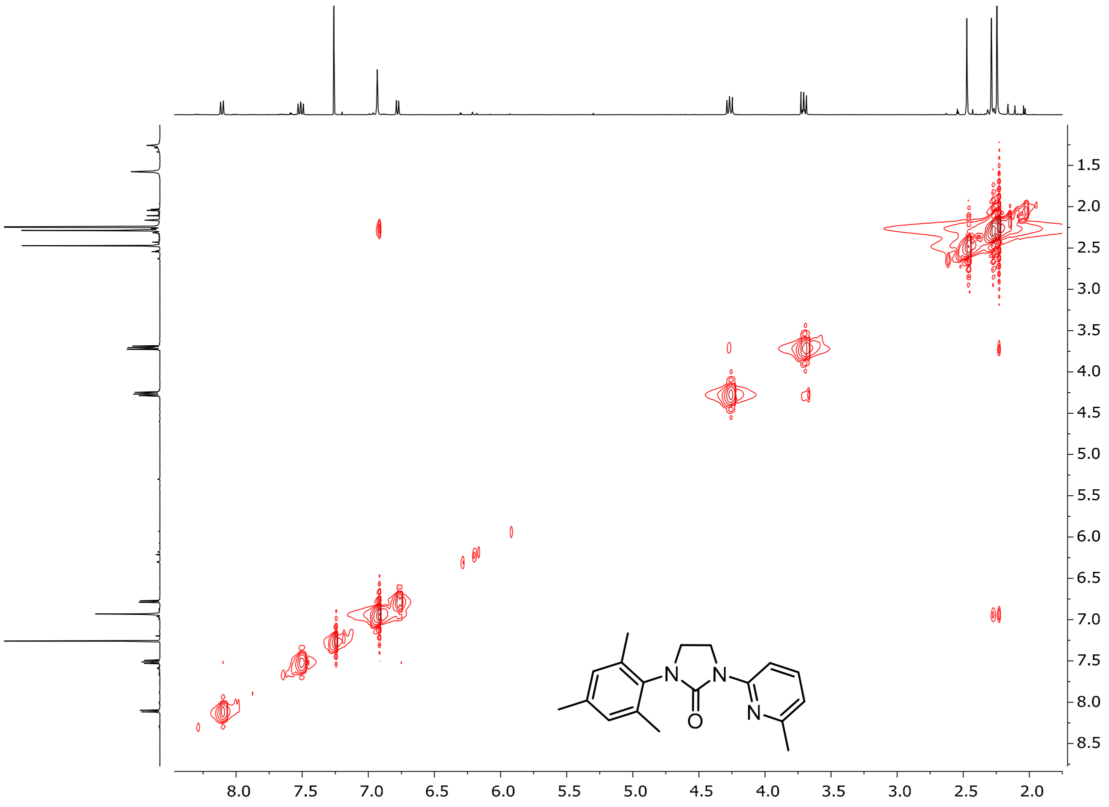

Figure S62. $^{1}H,^{1}H$-NOESY NMR (400MHz, 298K) of **L3^{ox}** in $CDCl_3$.

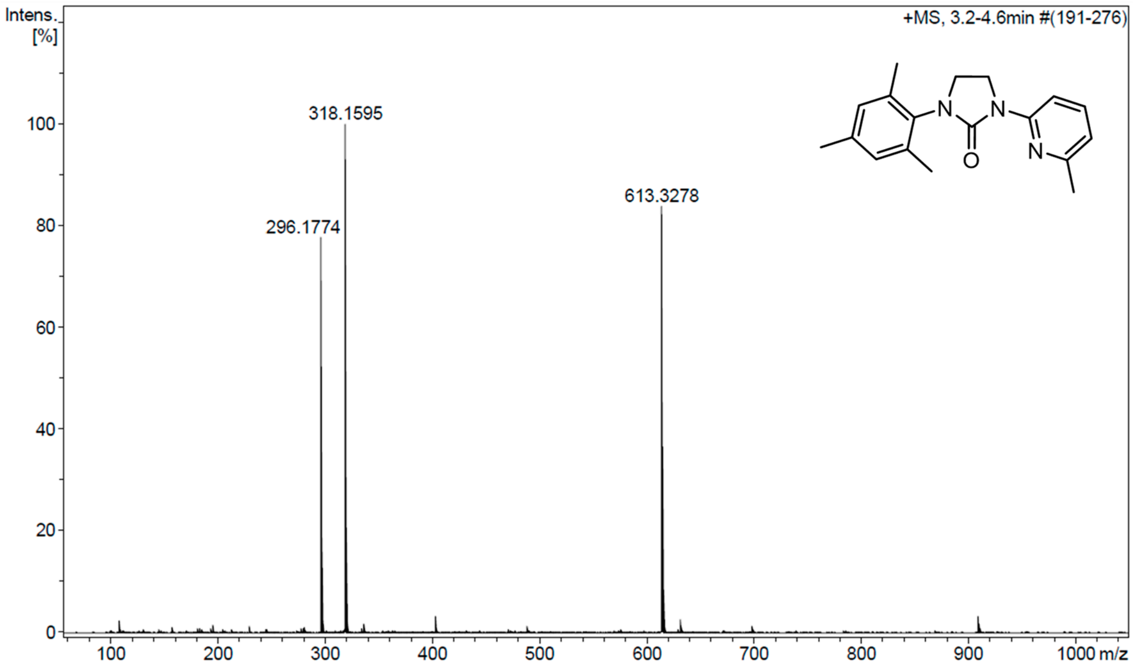

Figure S63. HRMS-ESI(+) of **L3^{ox}**.

S6. Experiments using water and ^{18}O-labeled water as additive

The origin of the oxygen atom in the formed imidazolinones was investigated by taking complex **2** as a case study and causing it to react with $PhI(OAc)_2$, employing water or 97% ^{18}O-labeled water as additive (Scheme S4).

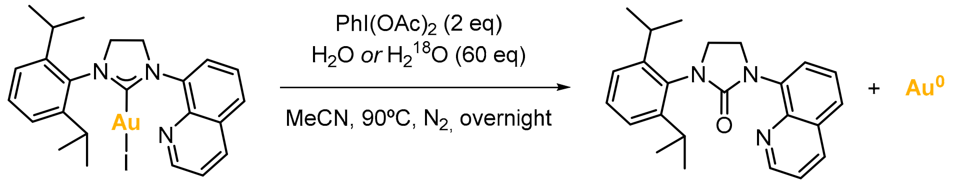

Scheme S4. Reaction of complex **2** with $PhI(OAc)_2$ as oxidant and water as additive.

Complex **2** (12.3 mg, 0.018 mmol, 1.0 eq) and $PhI(OAc)_2$ (12.1 mg, 0.038 mmol, 2.0 eq) were weighed in a vial. In the Schlenk line, the atmosphere of the vial was replaced with nitrogen. Then, anhydrous acetonitrile (0.8 mL) and water or ^{18}O-water (20 µL, 1.109 mmol, 61.6 eq) were added. The vial was sealed, and the mixture was allowed to react overnight at 90ºC. The reaction crude was a clear brown solution with Au(0) nuggets. The nuggets were separated by decantation, thoroughly washed with abundant acetonitrile several times, and dried. On the other hand, the solution was analyzed by HRMS-ESI to detect the formation of imidazolinone **L2^{ox}** and, when using $H_2{}^{18}O$, to detect and calculate the incorporation of ^{18}O (Figures S64 and S65). The imidazolinone **L2^{ox}** products were isolated by preparative TLC using a DCM:EtOAc (9:1) mixture as eluent.

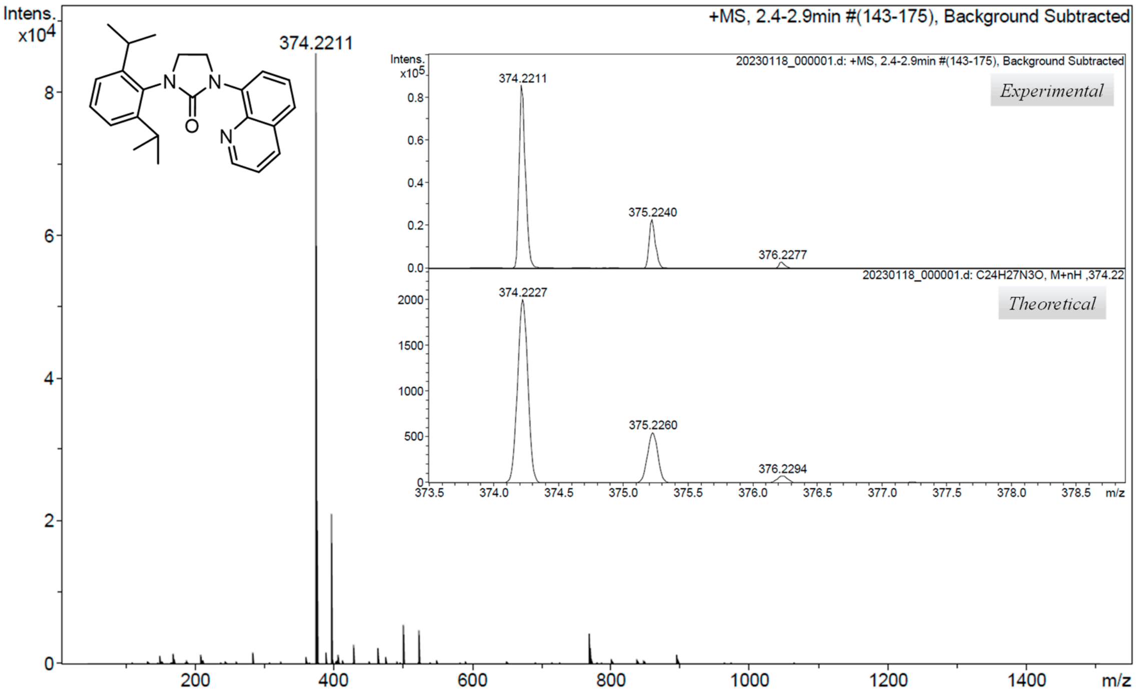

Figure S64. HRMS-ESI(+) of the reaction crude of complex **2**, $PhI(OAc)_2$ and water (Table S2, entry 1); close-up of the peak at *m/z* = 374.2 corresponding to the $[M+H]^+$ species for NHC=^{16}O; and comparison to the theoretical isotopic pattern.

For the reaction using $H_2{}^{18}O$, the percentage of ^{18}O-labeled imidazolinone was calculated using the intensities of the isotopic patterns of the peaks at 374.2 and 376.2, corresponding to NHC=^{16}O and NHC=^{18}O, respectively.

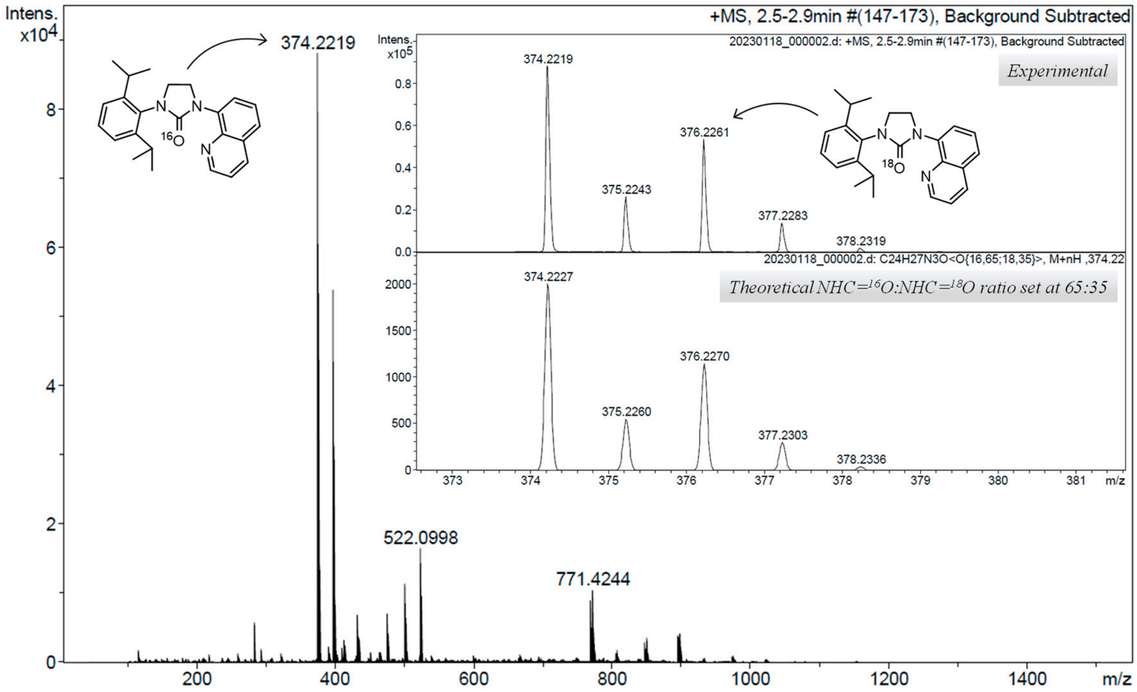

Figure S65. HRMS-ESI(+) of the reaction crude of complex **2**, $PhI(OAc)_2$ and ^{18}O-labeled water (Table S2, entry 2); close-up of the peaks at *m/z* = 374.2 and 376.2 corresponding to the $[M+H]^+$ species for NHC=^{16}O and NHC=^{18}O; and comparison to the theoretical isotopic pattern for an NHC=^{16}O:NHC=^{18}O ratio set at 65:35.

The results of the experiments using water are summarized in Table S2.

Table S2. Results of the reaction of complex **2** with $PhI(OAc)_2$ and water (Scheme S4).

Entry	Additive	Conv. Au(0)	[a]Yield NHC=O	[b]NHC=^{16}O/ NHC=^{18}O
1	H_2O	88%	48%	100/0
2	$H_2{}^{18}O$	97%	41%	65/35

[a] Isolated yield. [b] The ratio of non-labeled to labeled imidazolinone was calculated from the HRMS-ESI spectrum (Figure S65).

S7. X-Ray structures and crystallographic data

S7.1. Complex 1

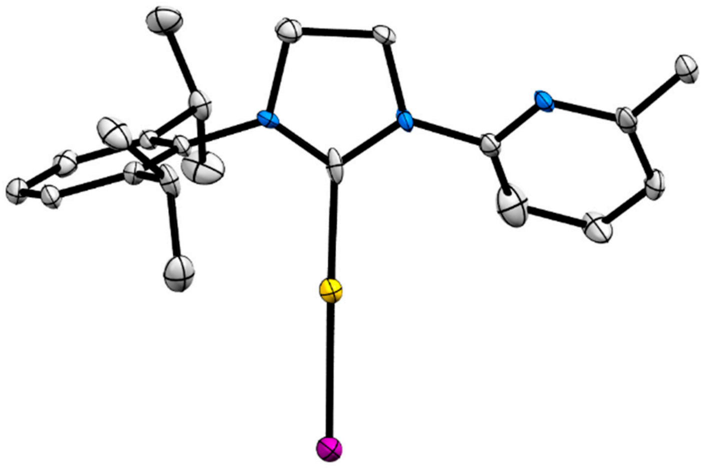

Figure S66. Crystal structure of **1** (CCDC 2238757). Ellipsoids set at 50% probability; H atoms are omitted for clarity.

Table S3. Crystallographic parameters for **1**.

**Chemical formula**	$C_{21}H_{27}AuIN_3$	
**Formula weight**	645.32 g/mol	
**Temperature**	100(2) K	
**Wavelength**	0.71073 Å	
**Crystal size**	0.010 x 0.050 x 0.300 mm	
**Crystal system**	Monoclinic	
**Space group**	P 1 21/n 1	
**Unit cell dimensions**	a = 11.191(2) Å	α = 90°
	b = 17.029(3) Å	β = 103.500(6)°
	c = 11.632(2) Å	γ = 90°
**Volume**	2155.5(7) Å^3	
**Density (calculated)**	1.988 g/cm^3	
**Absorption coefficient**	8.266 mm^{-1}	
**Final R indices**	3780 data; I>2σ(I)	R1 = 0.0611, wR2 = 0.1278
	All data	R1 = 0.1120, wR2 = 0.1550

A colorless needle-like specimen of $C_{21}H_{27}AuIN_3$, with approximate dimensions 0.010 mm x 0.050 mm x 0.300 mm, was used for the X-ray crystallographic analysis. A total of 542 frames were collected. The total exposure time was 2.00 hours. The frames were integrated with the Bruker SAINT software package using a narrow-frame algorithm. The integration of the data using a monoclinic unit cell yielded a total of 49421 reflections to a maximum θ angle of 28.52° (0.74 Å resolution), of which 5442 were independent

(average redundancy 9.081, completeness = 99.3%, R_{int} = 15.63%, R_{sig} = 7.44%) and 3780 (69.46%) were greater than 2σ(F2). The final cell constants of a = 11.191(2) Å, b = 17.029(3) Å, c = 11.632(2) Å, β = 103.500(6)°, volume = 2155.5(7) $Å^3$, are based upon the refinement of the XYZ-centroids of 9886 reflections above 20 σ(I) with 5.139° < 2θ < 56.75°. Data were corrected for absorption effects using the Numerical Mu From Formula method (SADABS). The ratio of minimum to maximum apparent transmission was 0.232. The calculated minimum and maximum transmission coefficients (based on crystal size) were 0.1910 and 0.9220. The structure was solved and refined by means of the Bruker SHELXTL Software Package using the space group P 1 21/n 1, with Z = 4 for the formula unit, $C_{21}H_{27}AuIN_3$. The final anisotropic full-matrix least-squares refinement on F^2, with 240 variables, converged at R1 = 6.11% for the observed data and wR2 = 15.50% for all data. The goodness-of-fit was 1.138. The largest peak in the final difference electron density synthesis was 4.529 $e^-/Å^3$, and the largest hole was -4.080 $e^-/Å^3$ with an RMS deviation of 0.334 $e^-/Å^3$. On the basis of the final model, the calculated density was 1.988 g/cm^3 and F(000), 1224 e^-.

S7.2. Complex 2

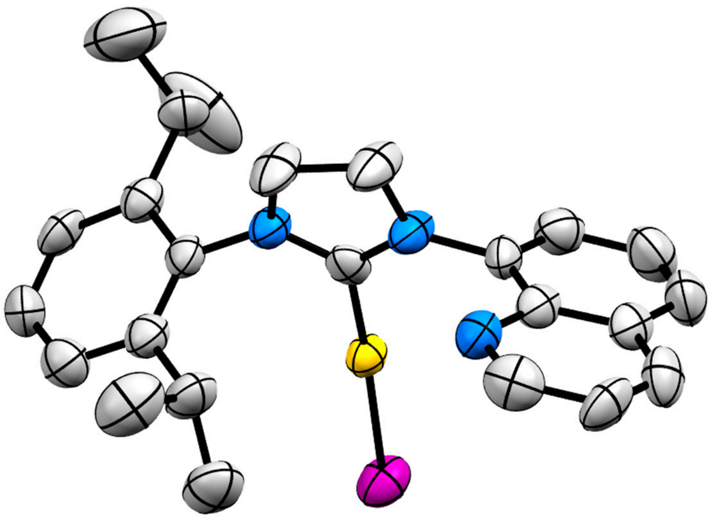

Figure S67. Crystal structure of **2** (CCDC2238754). Ellipsoids set at 50% probability; H atoms are omitted for clarity.

Table S4. Crystallographic parameters for **2**.

**Chemical formula**	$C_{24}H_{27}AuIN_3$	
**Formula weight**	681.35 g/mol	
**Temperature**	100(2) K	
**Wavelength**	0.71073 Å	
**Crystal size**	0.250 x 0.250 x 0.200 mm	
**Crystal system**	Monoclinic	
**Space group**	P 21/c	
**Unit cell dimensions**	a = 10.353(2) Å	α = 90°
	b = 13.845(3) Å	β = 104.203(4)°
	c = 17.507(4) Å	γ = 90°
**Volume**	2432.6(9) $Å^3$	
**Density (calculated)**	1.860 g/cm^3	
**Absorption coefficient**	7.331 mm^{-1}	
**Final R indices**	3439 data; I>2σ(I)	R1 = 0.0508, wR2 = 0.0925
	All data	R1 = 0.0873, wR2 = 0.1089

A yellow specimen of $C_{24}H_{27}AuIN_3$, with approximate dimensions 0.250 mm x 0.250 mm x 0.200 mm, was used for the X-ray crystallographic analysis. The measurement was carried out on a *BRUKER SMART APEX CCD* diffractometer using graphite-monochromated Mo *K*α radiation (λ = 0.71073 Å) from an x-Ray tube. The measurements were made in the range from 1.90 to 28.30° for θ. Hemi-sphere data collection was carried out with ω and φ scans. A total of 9725 reflections were collected, of which 5087 (R_{int} = 0.061) were unique. The programs used were the following: data collection, Smart; data reduction, Saint+; and absorption correction, SADABS. Structure solution and refinement were performed using SHELXL. The structure was solved by direct methods and refined by the full-matrix least-squares methods on F^2. The non-hydrogen atoms were refined anisotropically. The H-atoms were placed in geometrically optimized positions and forced to ride on the atoms to which they are attached.

S7.3. Complex 3

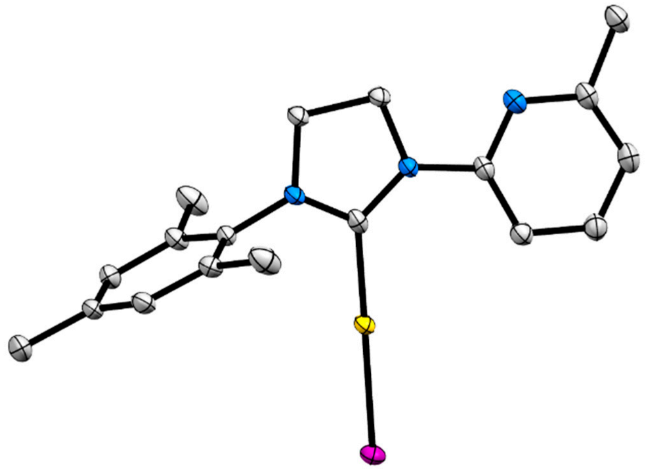

Figure S68. Crystal structure of **3** (CCDC 2238755). Ellipsoids set at 50% probability; H atoms are omitted for clarity.

Table S5. Crystallographic parameters for **3**.

**Chemical formula**	$C_{18}H_{21}AuIN_3$	
**Formula weight**	603.24 g/mol	
**Temperature**	100(2) K	
**Wavelength**	0.71073 Å	
**Crystal size**	0.150 x 0.200 x 0.200 mm	
**Crystal system**	Monoclinic	
**Space group**	P 1 21/c 1	
**Unit cell dimensions**	a = 9.1638(5) Å	α = 90°
	b = 12.7701(7) Å	β = 102.7840(10)°
	c = 15.9946(8) Å	γ = 90°
**Volume**	1825.33(17) Å^3	
**Density (calculated)**	2.195 g/cm^3	
**Absorption coefficient**	9.754 mm^{-1}	
**Final R indices**	5734 data; I>2σ(I)	R1 = 0.0240, wR2 = 0.0500
	All data	R1 = 0.0307, wR2 = 0.0526

A colorless block-like specimen of $C_{18}H_{21}AuIN_3$, with approximate dimensions of 0.150 mm x 0.200 mm x 0.200 mm, was used for the X-ray crystallographic analysis. A total of 428 frames were collected. The total exposure time was 1.19 hours. The frames were integrated with the Bruker SAINT software package using a narrow-frame algorithm. The integration of the data, using a monoclinic unit cell, yielded a total of 37990 reflections to a maximum θ angle of 32.21° (0.67 Å resolution), of which 6435 were independent (average redundancy 5.904, completeness = 99.7%, R_{int} = 4.63%, R_{sig} = 2.97%) and 5734

(89.11%) were greater than 2σ(F2). The final cell constants of a = 9.1638(5) Å, b = 12.7701(7) Å, c = 15.9946(8) Å, β = 102.7840(10)°, volume = 1825.33(17) $Å^3$, were based upon the refinement of the XYZ-centroids of 121 reflections above 20 σ(I) with 6.568° < 2θ < 49.17°. Data were corrected for absorption effects using the Numerical Mu From Formula method (SADABS). The ratio of minimum to maximum apparent transmission was 0.221. The calculated minimum and maximum transmission coefficients (based on crystal size) were 0.2460 and 0.3220. The structure was solved and refined by means of the Bruker SHELXTL Software Package, using the space group P 1 21/c 1, with Z = 4 for the formula unit, $C_{18}H_{21}AuIN_3$. The final anisotropic full-matrix least-squares refinement on F^2 with 212 variables converged at R1 = 2.40% for the observed data and wR2 = 5.26% for all data. The goodness-of-fit was 1.096. The largest peak in the final difference electron density synthesis was 1.621 $e^-/Å^3$, and the largest hole was -0.850 $e^-/Å^3$ with an RMS deviation of 0.156 $e^-/Å^3$. On the basis of the final model, the calculated density was 2.195 g/cm^3 and F(000), 1128 e^-.

S7.4. Complex 4

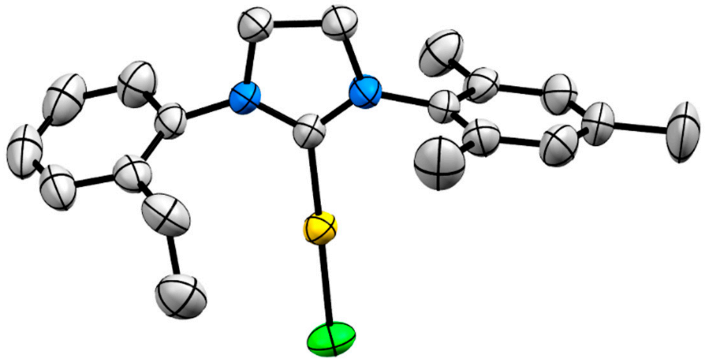

Figure S69. Crystal structure of **4** (CCDC 2238756). Ellipsoids set at 50% probability; H atoms are omitted for clarity.

Table S6. Crystallographic parameters for **4**.

**Chemical formula**	$C_{20}H_{24}AuClN_2$	
**Formula weight**	524.83 g/mol	
**Temperature**	300(2) K	
**Wavelength**	0.71076 Å	
**Crystal size**	0.080 x 0.100 x 0.280 mm	
**Crystal system**	Monoclinic	
**Space group**	P 1 21/c 1	
**Unit cell dimensions**	a = 19.07(2) Å	α = 90°
	b = 8.236(11) Å	β = 92.08(4)°
	c = 12.435(15) Å	γ = 90°
**Volume**	1952.(4) Å^3	
**Density (calculated)**	1.786 g/cm^3	
**Absorption coefficient**	7.678 mm^{-1}	
**Final R indices**	3779 data; I>2σ(I)	R1 = 0.0312, wR2 = 0.0630
	All data	R1 = 0.0705, wR2 = 0.0724

A colorless needle-like specimen of $C_{20}H_{24}AuClN_2$, with approximate dimensions of 0.080 mm x 0.100 mm x 0.280 mm, was used for the X-ray crystallographic analysis. The X-ray intensity data were measured on a three-circle diffractometer system equipped with a ceramic x-ray tube (Mo Kα, λ = 0.71076 Å) and a doubly curved silicon crystal Bruker Triumph monochromator. A total of 1734 frames were collected. The total exposure time was 13.00 hours. The frames were integrated with the Bruker SAINT software package using a narrow-frame algorithm. The integration of the data using a monoclinic unit cell yielded a total of 112940 reflections to a maximum θ angle of 29.64° (0.72 Å resolution), of which 4883 were independent (average redundancy 23.129, completeness = 88.6%, R_{int} = 6.71%, R_{sig} = 3.82%) and 3779 (77.39%) were greater than 2σ(F2). The final cell constants of a = 19.07(2) Å, b = 8.236(11) Å, c = 12.435(15) Å, β = 92.08(4)°, volume = 1952.(4) Å^3, were based upon the refinement of the XYZ-centroids of 9603 reflections above 20 σ(I), with 5.892° < 2θ < 53.88°. Data were corrected for absorption effects using the Multi-Scan method (SADABS). The ratio of minimum to maximum apparent transmission was 0.552. The calculated minimum and maximum transmission coefficients (based on crystal size) were 0.4117 and 0.7455. The structure was solved and refined by means of the Bruker SHELXTL Software Package, using the space group P 1 21/c 1, with Z = 4 for the formula unit, $C_{20}H_{24}AuClN_2$. The final anisotropic full-matrix least-squares refinement on F^2 with 221 variables converged at R1 = 3.12% for the observed data and wR2 = 7.24% for all data. The goodness-of-fit was 1.176. The largest peak in the final difference electron density synthesis was 1.706 e$^-$/Å^3, and the largest hole was -2.804 e$^-$/Å^3 with an RMS deviation of 0.183 e$^-$/Å^3. On the basis of the final model, the calculated density was 1.786 g/cm^3 and F(000), 1016 e$^-$.

S8. SEM-EDX characterization of Au(0) macroaggregates

In order to analyze the obtained Au(0) macroaggregates on a general basis, from the reactions of Au(I) complexes with oxidants (Section 4), the reaction depicted in Scheme S4 was selected as the case study. The Au(0) nuggets were isolated from the reaction outcome by decantation, then washed with abundant 1,2-DCE and, finally, dried under vacuum. The dried Au(0) nuggets were morphologically characterized by scanning electron microscopy (SEM), and energy-dispersive x-ray spectroscopy (EDX) allowed for the analysis of the elemental composition.

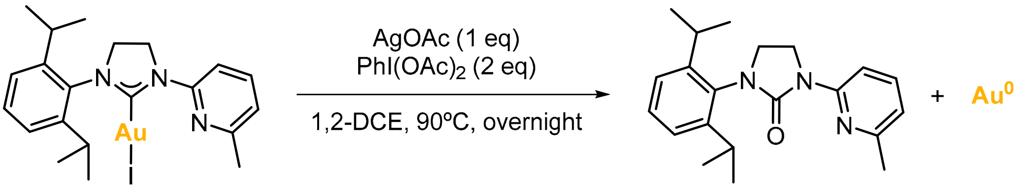

1 **L1^{ox}**

Scheme S5. Formation of product **L1^{ox}** and Au(0) macroaggregates from complex **1**.

S8.1. SEM images

The images displayed herein show that the Au(0) macroaggregates have irregular shapes and sizes, reaching particle lengths of up to ~500μm. The surfaces of the particles are irregular and feature both granulated and flat domains.

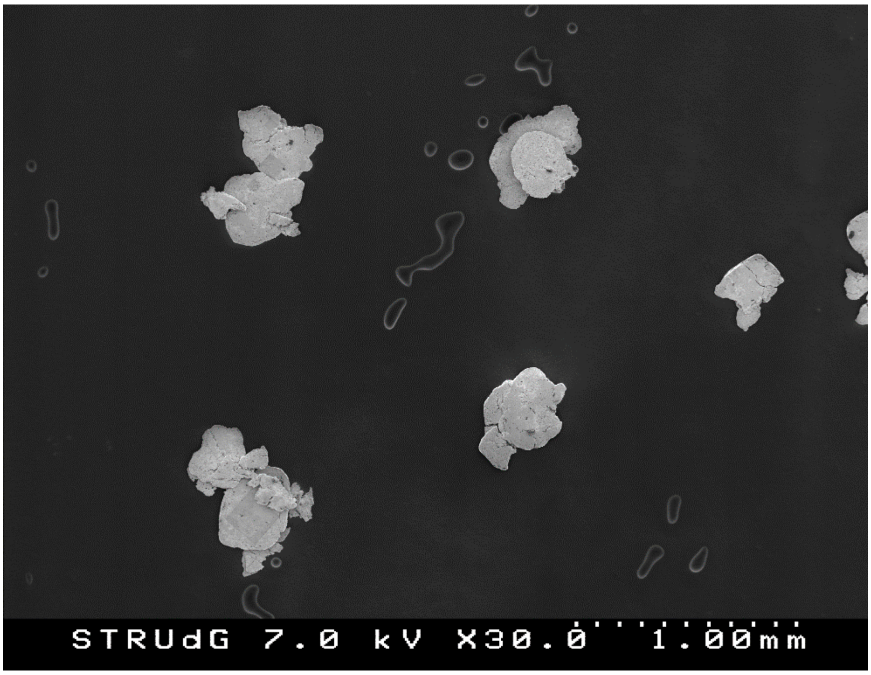

Figure S70. SEM image at x30.

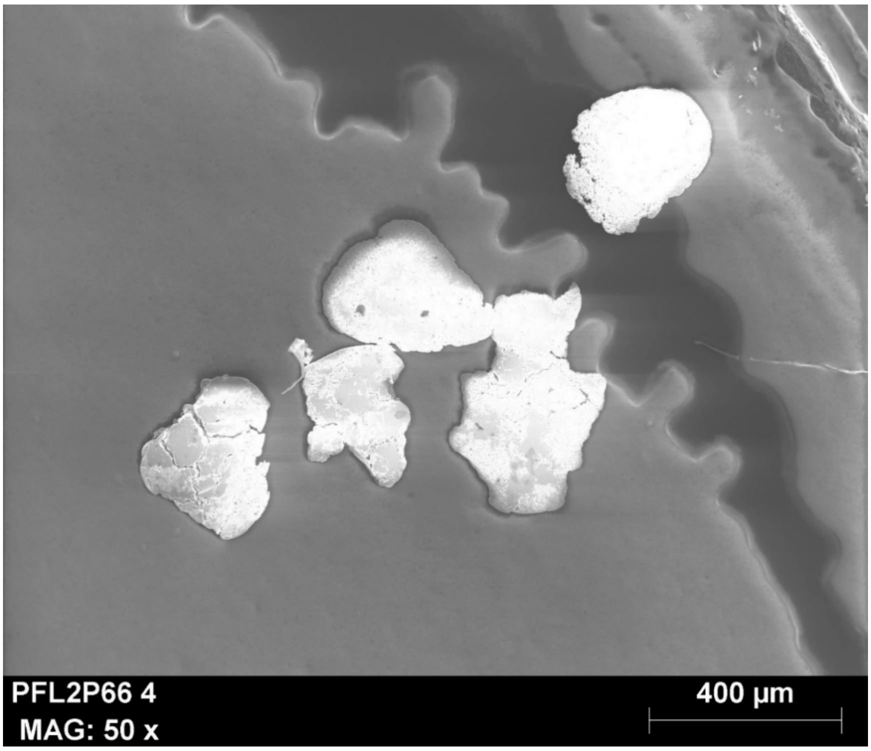

Figure S71. SEM image at x50.

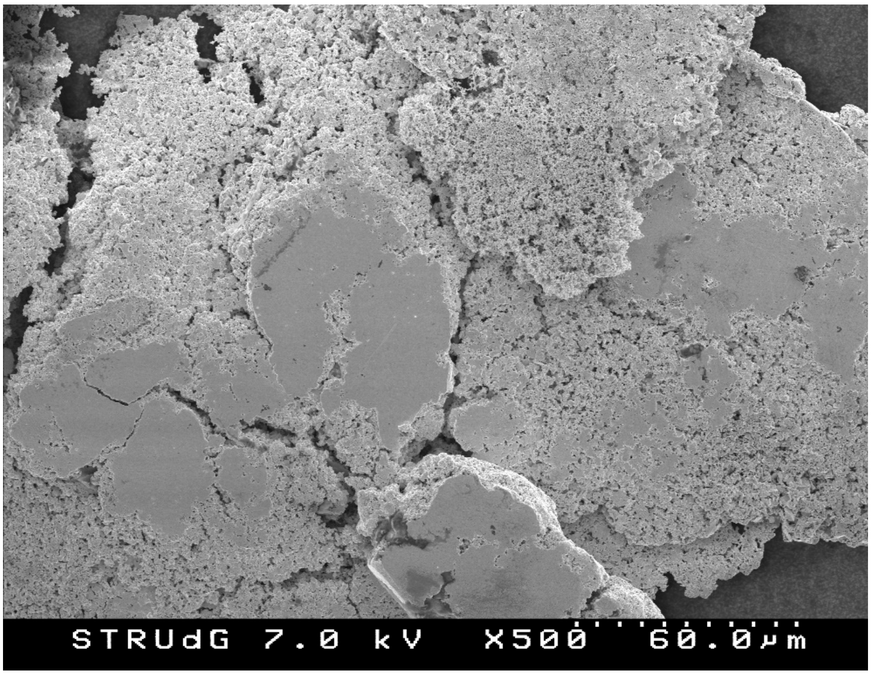

Figure S72. SEM image at x500.

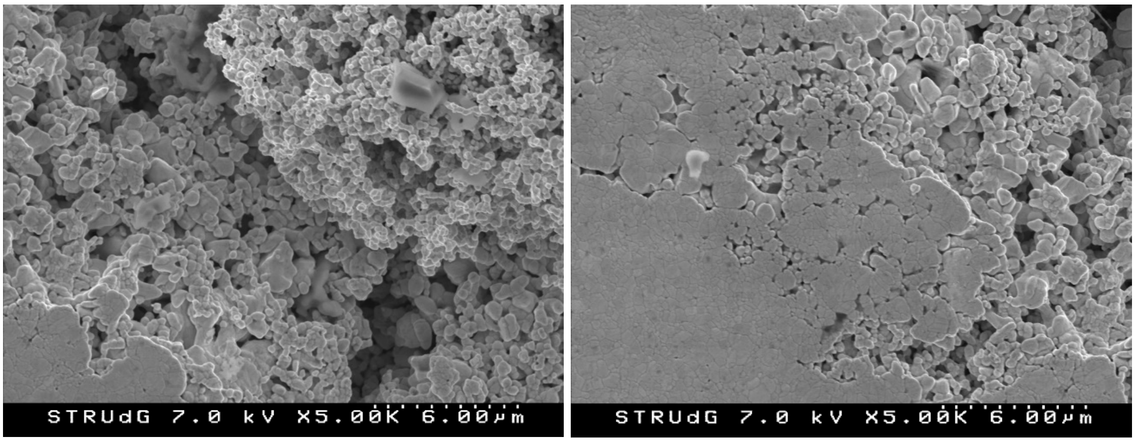

Figure S73. SEM images at x5K.

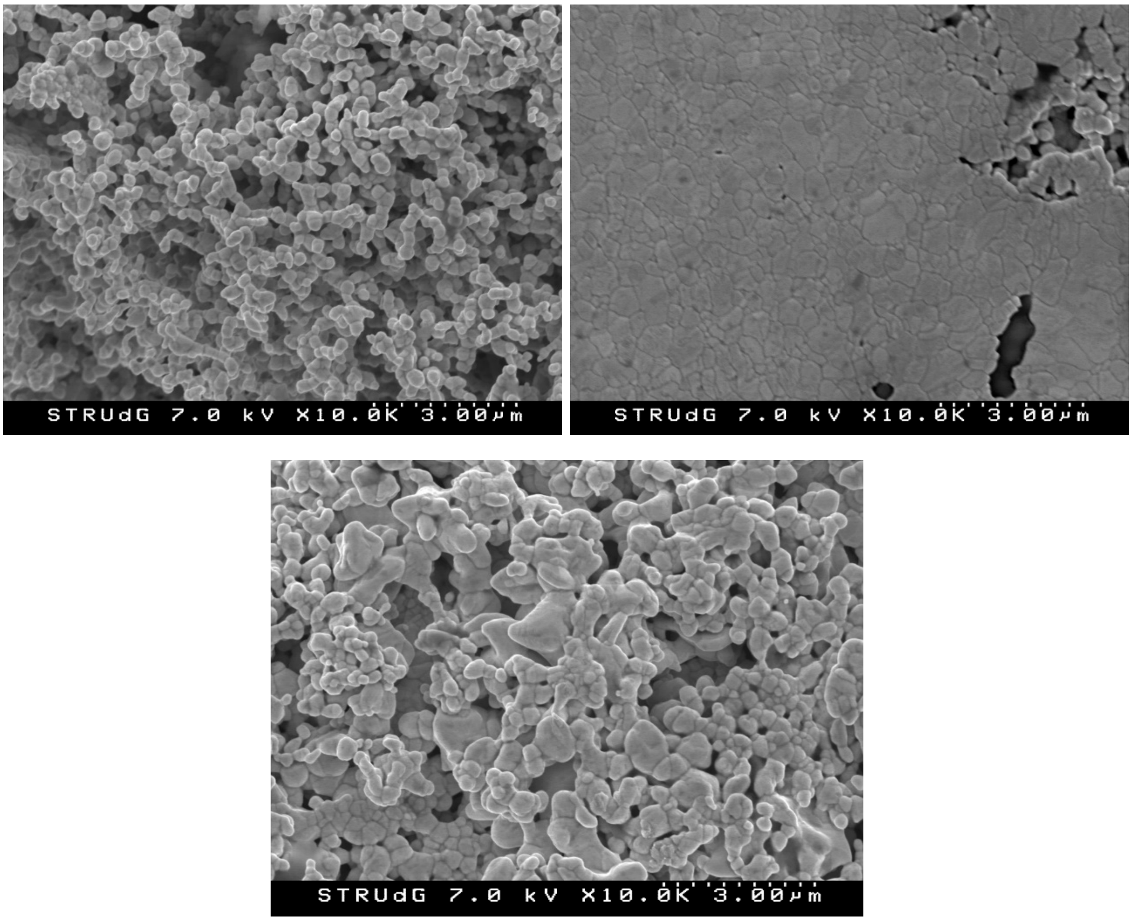

Figure S74. SEM images at x10K.

S8.2. SEM-EDX analysis

The determination of the elemental composition of the particles was carried out at morphologically different regions of the particles. All of them showed high purity on Au, and the observed C and N could be attributed to impurities from the carbon tape support.

SEM-EDX of a granulated region

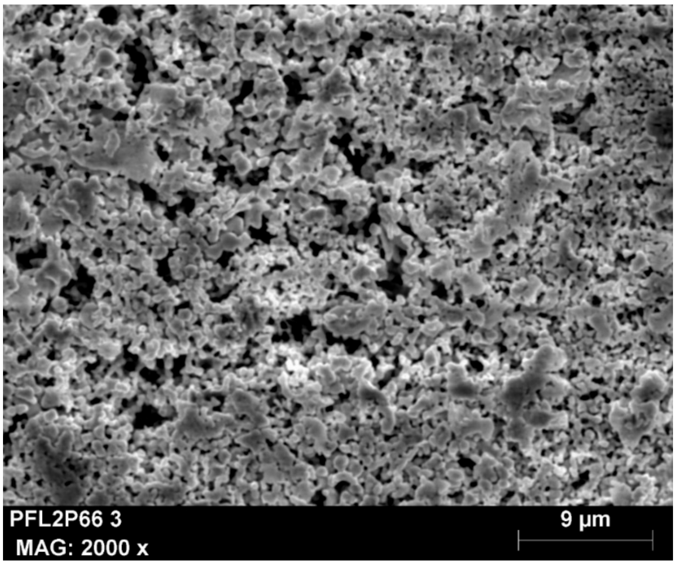

Figure S75. Granulated zone analyzed by SEM-EDX.

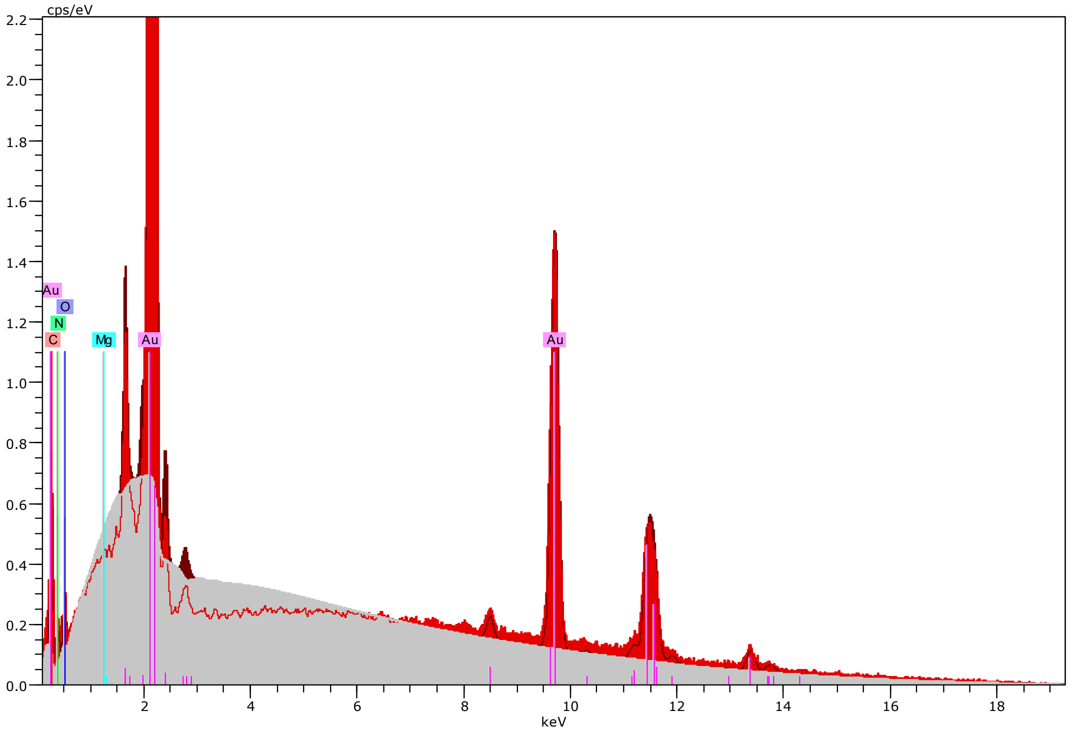

Figure S76. EDX spectrum of a granulated region.

Element	Series	unn. C [wt.%]	norm. C [wt.%]	Atom. C [at.%]
Carbon	K-series	9.22	7.21	45.01
Nitrogen	K-series	3.29	2.57	13.77
Oxygen	K-series	2.05	1.60	7.51
Magnesium	K-series	0.00	0.00	0.00
Gold	L-series	113.18	88.61	33.71
	Total:	127.73	100.00	100.00

SEM-EDX of a flat region

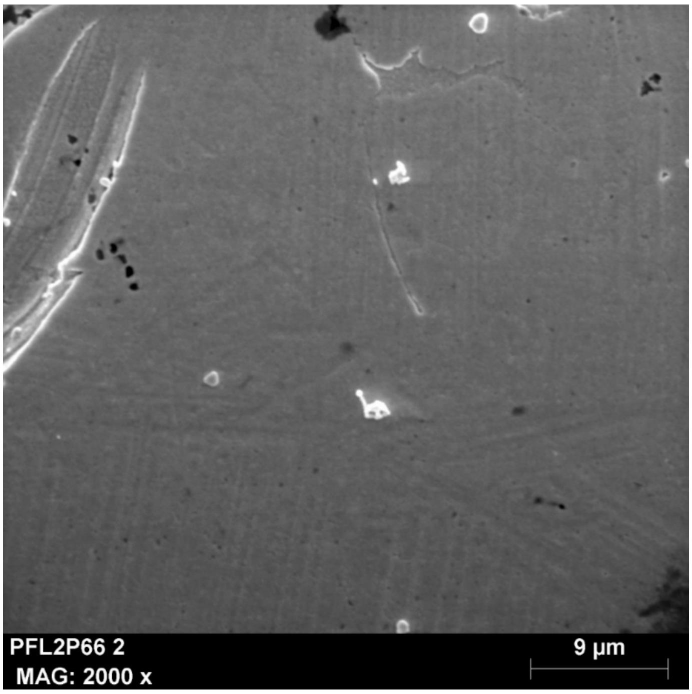

Figure S77. Flat zone analyzed by SEM-EDX.

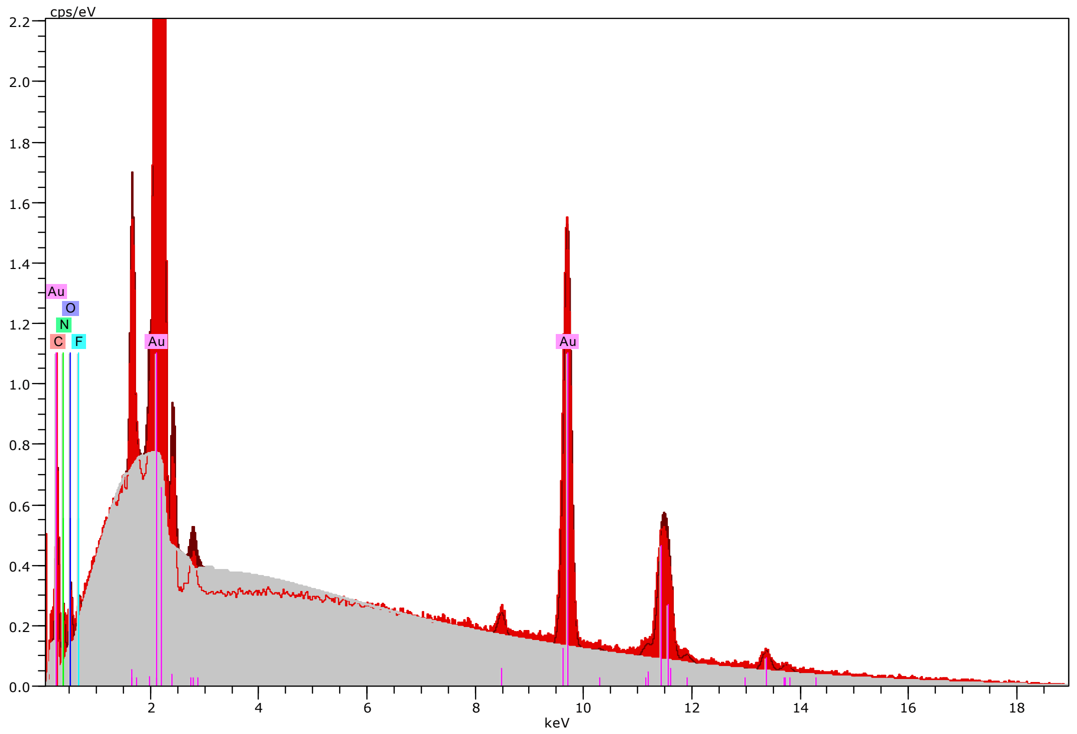

Figure S78. EDX spectrum of a flat region.

Element	Series	unn. C [wt.%]	norm. C [wt.%]	Atom. C [at.%]
Carbon	K-series	8.07	6.91	42.01
Nitrogen	K-series	3.50	2.99	15.61
Oxygen	K-series	1.97	1.69	7.71
Fluorine	K-series	0.64	0.55	2.11
Gold	L-series	102.62	87.85	32.56
	Total:	116.81	100.00	100.00

SEM-EDX of a mixed zone, with granulated and flat regions.

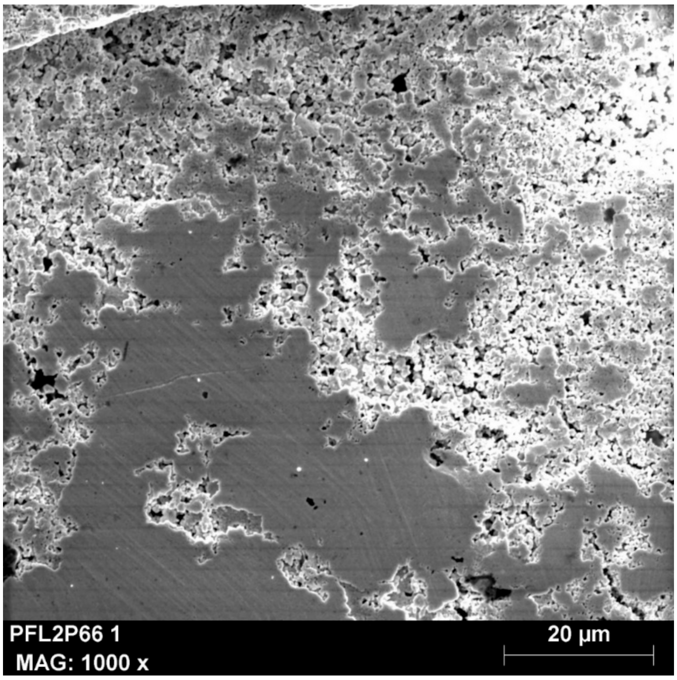

Figure S79. Mixed zone analyzed by SEM-EDX.

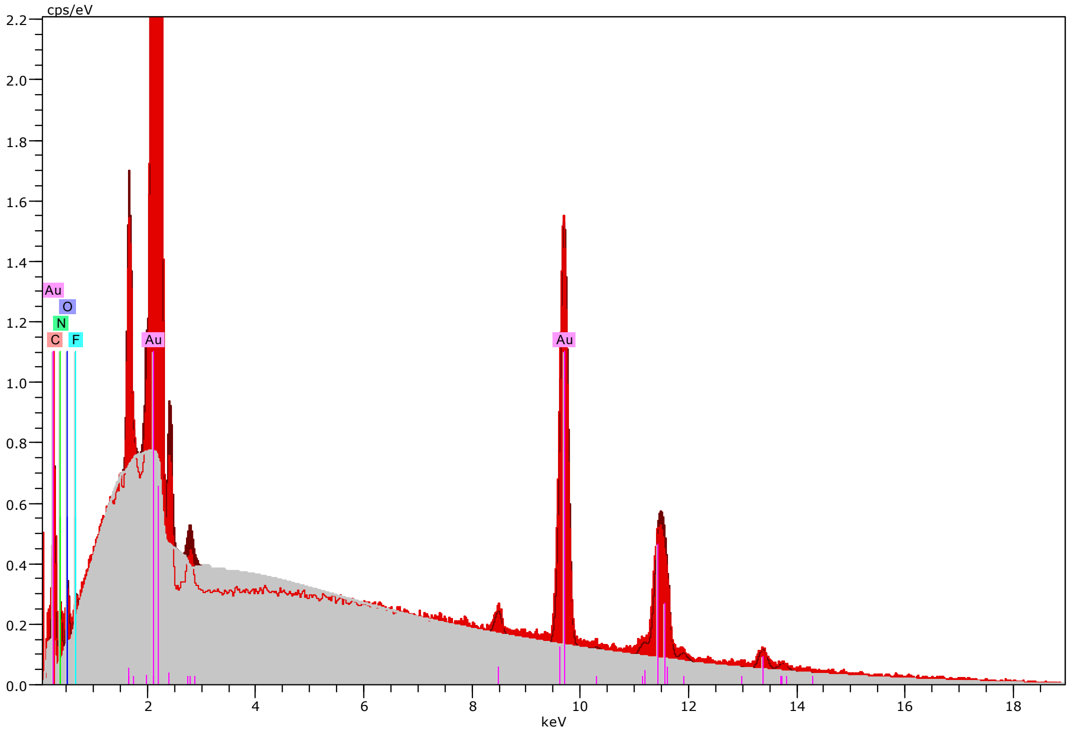

Figure S80. EDX spectrum of a mixed zone.

Element	Series	unn. C [wt.%]	norm. C [wt.%]	Atom. C [at.%]
Carbon	K-series	7.69	6.12	39.78
Nitrogen	K-series	3.35	2.67	14.87
Oxygen	K-series	1.85	1.47	7.18
Fluorine	K-series	0.87	0.69	2.85
Gold	L-series	111.97	89.05	35.32
	Total:	125.73	100.00	100.00

S9. Heterogeneous Au(0) catalysis attempts

Isolated Au(0) nuggets were tested as heterogeneous catalysts in coupling reactions, such as in Sonogashira, A3 and Glaser couplings.

Sonogashira coupling

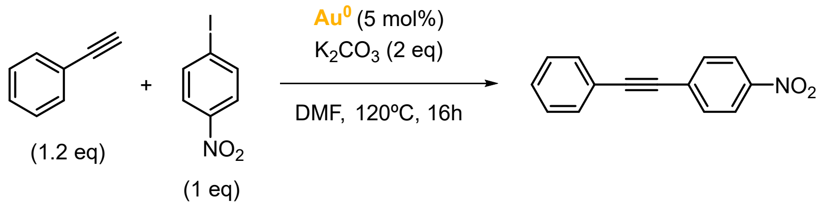

Scheme S6. Sonogashira coupling attempt using Au(0) nuggets as heterogeneous catalysts.

By analyzing the reaction crude by GC-MS, the desired coupling product was not detected.

A3 coupling

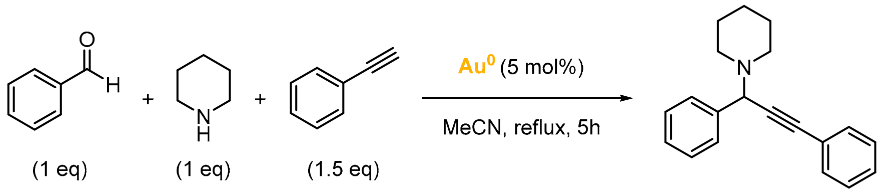

Scheme S7. A3 coupling attempt using Au(0) nuggets as heterogeneous catalysts.

By analyzing the reaction crude by GC-MS, traces of the desired coupling product were detected, as well as in the blank experiment. Thus, no relevant catalytic activity was attributed to gold(0).

Glaser coupling

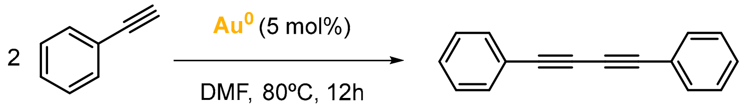

Scheme S8. Glaser coupling attempt using Au(0) nuggets as heterogeneous catalysts.

By analyzing the reaction crude by GC-MS, the desired homocoupling product was not detected.

S10. References

24. Papastavrou, A. T.; Pauze, M.; Gómez-Bengoa, E.; Vougioukalakis, G. C., Unprecedented Multicomponent Organocatalytic Synthesis of Propargylic Esters via CO2 Activation. *ChemCatChem* **2019**, *11*, 5379–5386.
25. Liori, A. A.; Stamatopoulos, I. K.; Papastavrou, A. T.; Pinaka, A.; Vougioukalakis, G. C., A Sustainable, User-Friendly Protocol for the Pd-Free Sonogashira Coupling Reaction. *Eur. J. Org. Chem.* **2018**, *2018*, 6134–6139.
44. Prasad, B.; Gilbertson, S., One-Pot Synthesis of N-Heterocyclic Carbene Ligands From a N-(2-iodoethyl)arylamine salts. *Org. Lett.* **2009**, *11*, 3710–3713.
